# Synthesis and crystal structures of two new tin bis­(carboranylamidinate) complexes

**DOI:** 10.1107/S2056989017012671

**Published:** 2017-09-12

**Authors:** Nicole Harmgarth, Phil Liebing, Philipp Hillebrand, Sabine Busse, Frank T. Edelmann

**Affiliations:** a Chemisches Institut der Otto-von-Guericke-Universität Magdeburg, Universitätsplatz 2, 39106 Magdeburg, Germany; b ETH Zurich, Laboratory for Inorganic Chemistry, Vladimir-Prelog-Weg 1-5/10, 8093, Zurich, Switzerland

**Keywords:** crystal structure, carborane, amidinate, carboranylamidinate, tin

## Abstract

Reaction of 2 equiv. of the lithium carboranylamidinate Li[*o*-(C_2_H_10_B_10_)C(NCy)(NHCy)] with SnCl_2_ in THF afforded the stannylene compound bis(*N*,*N*′-dicyclohexylamidinatocarboranate)tin(II), Sn^II^[*o*-(C_2_H_10_B_10_)C(NCy)(NHCy)]_2_ (**1**). A similar reaction of SnCl_4_ with 2 equiv. of Li[*o*-(C_2_H_10_B_10_)C(N^*i*^Pr)(NH^*i*^Pr)] unexpectedly afforded the known solvated penta­chlorido­stannate(IV) salt [Li(THF)_4_][SnCl_5_(THF)] as the main reaction product. Small amounts of the new chlorido-tin(IV) bis­(carboranylamidinate) bis(*N*,*N*′-diiso­propylamidinatocarboranate)chloridotin(IV), Sn^IV^Cl[*o*-(C_2_H_10_B_10_)C(N^*i*^Pr)(NH^*i*^Pr)][*o*-(C_2_H_10_B_10_)C(N^*i*^Pr)_2_] (**2**), were isolated as a by-product. Single-crystal X-ray structure analysis revealed a *κC*,*κN*-chelating coordination of the carboranylamidinate ligands in both **1** and **2**. The Sn atom in **1** adopts a pseudo-trigonal–bipyramidal coordination under participation of a stereoactive lone pair. In **2**, a trigonal–bipyramidal coordination of Sn is completed by a chlorido ligand.

## Abstract 2

Tin forms mononuclear chelate complexes with *κC*,*κN*-coordinated carboranylamidinate ligands, in which the Sn atom exhibits a trigonal–bipyramidal (Sn^IV^) or pseudo-trigonal–bipyramidal (Sn^II^) coordination.

## Chemical context   

Amidinates of the general formula [*R*C(N*R*′)_2_]^−^ are the nitro­gen analogs of carboxyl­ate anions. These versatile *N*,*N*′-chelating ligands form stable coordination compounds with nearly every metallic element in the Periodic Table. In view of this rich coordination chemistry, amidinate ligands are frequently regarded as ‘steric cyclo­penta­dienyl equivalents’. Metal complexes comprising amidinato ligands are readily available by insertion of the 1,3-diorganocarbodi­imide, *R–*-N=C=N—*R*, into an *M*—C bond of an organometallic precursor compound. Another common synthetic route involves salt metathetical reactions between lithium amidinates and metal halides (Collins, 2011 ▸; Edelmann, 2008 ▸, 2013*a* ▸,*b* ▸). Metal amidinates comprising small alkyl substituents are often surprisingly volatile and may serve as useful precursors for metal oxides and nitrides by means of ALD (atomic layer deposition) or MOVCD (metal–organic chemical vapor deposition) processes (Devi 2013 ▸; Lim *et al.*, 2003 ▸; Li *et al.*, 2005 ▸).

**Figure 1:**
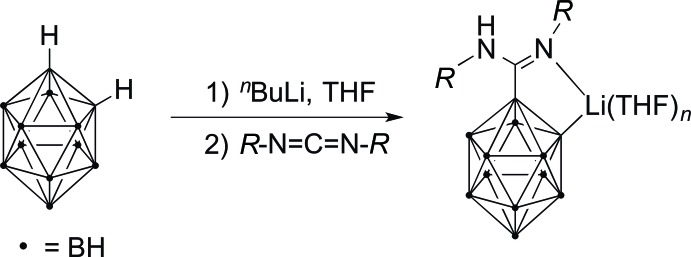
Synthesis of lithium *ortho*-carboranylamidinates Li[H*L*] from the parent *ortho*-carborane.

**Figure 2:**
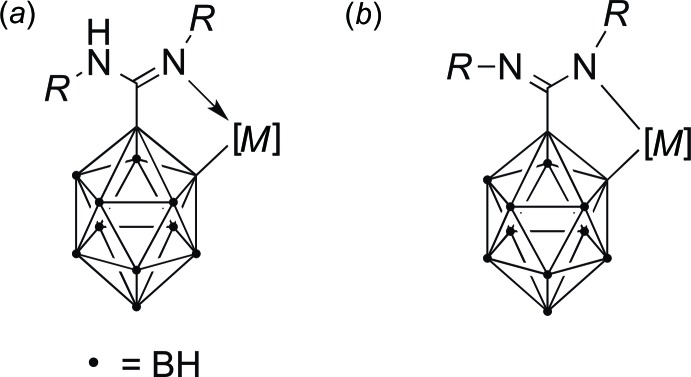
Two types of *ortho*-carboranyl­amidine-derived ligands: monoanionic with protonated amidinate moiety (*a*) and dianionic with a covalent *M*—N bond (*b*).

A key advantage of the amidinate anions [*R*C(N*R*′)_2_]^−^ is the fact that the substituents *R* and *R*′ attached to the N–C–N unit can be varied in many ways. With *R* = *ortho*-C_2_H_11_B_10_ (‘*ortho*-carboran­yl’) we introduced a sterically demanding and chemically versatile moiety in the backbone of the amidinate ligand. Carboranes are of tremendous scientific and technological inter­est due to their various applications ever since their discovery in the 1960′s. These applications include the synthesis of polymers and ceramics, catalysts, radiopharmaceuticals and non-linear optics, as well as the BNCT (= boron neutron capture therapy) technique (Belmont *et al.*, 1989 ▸; Brown *et al.*, 1992 ▸; Felekidis *et al.*, 1997 ▸; Murphy *et al.*, 1993 ▸; Teixidor *et al.*, 1996 ▸; Vaillant *et al.*, 2002 ▸). The first *ortho*-carboranylamidinate ligand [*o*-(C_2_H_10_B_10_)C(N^*i*^Pr)(NH^*i*^Pr)]^−^ (= [H*L^i^* ^Pr^]^−^) was synthesized in our lab in 2010 by *in situ* li­thia­tion of the parent *o*-carborane, *ortho*-C_2_H_12_B_10_ (= *ortho*-dicarba-*closo*-dodeca­borane), followed by treatment with 1 equiv. of *N,N′*-diiso­propyl­carbodi­imide (Dröse *et al.*, 2010 ▸) as shown in Fig. 1 ▸. Subsequent reactions of the so-obtained lithium *ortho*-carboranylamidinate Li[H*L^i^* ^Pr^] or the related Li[H*L* ^Cy^] with various metal and non-metal chlorides have been reported by us and others to yield carboranylamidinates of *e.g.* Sn^II^ and Cr^II^, Rh^I^ and Ir^I^, Fe^II^ and Fe^III^, Ti^IV^, Zr^IV^, Si and P (Harmgarth *et al.*, 2014 ▸; Hillebrand *et al.*, 2014 ▸; Yao *et al.*, 2011 ▸, 2012 ▸, 2013 ▸; Xu *et al.*, 2014 ▸). In all of these compounds, the ligand adopts a specific κ*C*,κ*N*-chelating mode instead of the κ*N*,κ*N′*-chelating mode usually observed for simple amidinate ligands (Collins, 2011 ▸; Edelmann, 2008 ▸, 2013*a* ▸,*b* ▸). In the case of the carboranylamidinates [H*L^R^*]^−^ (*R* = ^*i*^Pr, Cy), a proton is formally shifted from the carborane C atom to the amidinate unit, resulting in an amidine moiety that usually acts as a monodentate donor functionality as shown in Fig. 2 ▸ *a*.

In some cases, subsequent deprotonation of the NH functionality results in formation of a formally dianionic ligand [*L^R^*]^2–^, whose favored coordination mode is still κ*C*,κ*N* (Fig. 2 ▸ *b*). Derivatives of Si, P, Ge, Sn^II^, Sn^IV^, Fe^II^ and Fe^III^, Rh^I^ and Ir^I^ containing this ligand system have been prepared by double li­thia­tion of the parent *ortho*-carboranyl­amidine followed by treatment with appropriate element chloride precursors (Yao *et al.*, 2011 ▸; Harmgarth *et al.*, 2014 ▸, 2017 ▸), or through spontaneous disproportionation of *in situ* formed [H*L^R^*]^−^ to [*L^R^*]^2–^ and free carboranyl­amidine H_2_ *L^R^*. The latter reaction has been found to be favored in the case of strongly Lewis-acidic metal precursors, namely Cp_2_TiCl_2_, Cp_2_ZrCl_2_ and various chloro­silanes (Harmgarth *et al.*, 2014 ▸, 2017 ▸). While di­chloro­silanes *R*_2_SiCl_2_ react with Li[H*L^i^* ^Pr^] readily to form *R*_2_Si[*L^i^* ^Pr^]-type products, we recently found that for the heavy group 14 analogues Sn and Pb the formation of *R*_2_ECl[H*L^i^* ^Pr^]-type products is much more preferred (Harmgarth *et al.*, 2017 ▸).

Among the known carboranylamidinate complexes are only very few compounds with more than one carboranylamidinate ligand per metal atom, and these are exclusively of the type *M*[H*L^R^*]_2_ (*M* = Sn^II^, Cr^II^, Dröse *et al.*, 2010 ▸; *M* = Co^II^, Ni^II^, Cu^II^, Yao *et al.*, 2012 ▸; *M* = Fe^II^, Fe^III^Cl, Hillebrand *et al.*, 2014 ▸). We report here the formation and structural characterization of two new tin bis­(carboranylamidinate) complexes, namely Sn^II^[*o*-(C_2_H_10_B_10_)C(NCy)(NHCy)]_2_ (= Sn[H*L* ^Cy^]_2_; **1**) and Sn^IV^Cl[*o*-(C_2_H_10_B_10_)C(N^*i*^Pr)(NH^*i*^Pr)][*o*-(C_2_H_10_B_10_)C(N^*i*^Pr)_2_] (= SnCl[*L^i^* ^Pr^][H*L^i^* ^Pr^]; **2**). Compound **2** is the first carboranylamidinate complex containing both mono- and dianionic carboranylamidinate ligands in a single mol­ecule.
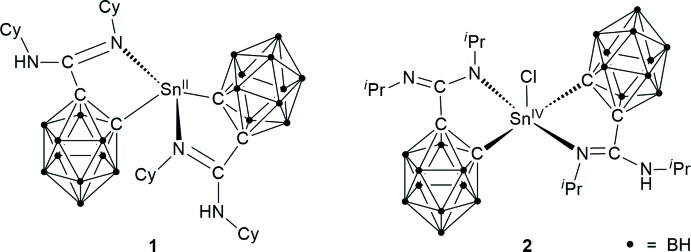


The mono-li­thio-*ortho*-carboranylamidinate precursors Li[H*L* ^Cy^] and Li[H*L^i^* ^Pr^] were readily available following a published procedure by reaction of the mono-li­thia­ted *o*-carborane Li-*o*-C_2_B_10_H_11_ with a stoichiometric amount of the carbodi­imides ^*i*^PrN=C=N^*i*^Pr or CyN=C=NCy, respectively, in THF (*cf*. Fig. 1 ▸) (Harmgarth *et al.*, 2014 ▸). In a first experiment, reaction of Li[H*L* ^Cy^] with 0.5 equiv. of SnCl_2_ in THF afforded the stannylene compound Sn[H*L* ^Cy^]_2_ (**1**) as colorless, block-like single crystals after recrystallization from toluene. The low isolated yield of *ca* 20% can be traced back to the very high solubility of **1** even in non-polar organic solvents. In addition to the X-ray diffraction study, compound **1** was also characterized through elemental analysis and the usual set of spectroscopic methods. In the IR spectrum, a characteristic *ν*(NH) band at 3423 cm^−1^ confirmed the presence of monoanionic [H*L* ^Cy^]^−^ ligands. The NH functionalities were also observed in the ^1^H NMR spectrum through a broad singlet at *δ* 4.50 ppm. A single ^119^Sn NMR resonance at *δ* −46 ppm was in agreement with the formation of a single Sn-containing species. The mass spectrum of **1** showed the mol­ecular ion at *m*/*z* 818 with 27% relative intensity.

A similar reaction of SnCl_4_ with 2 equiv. of Li[H*L^i^* ^Pr^] was carried out with the aim of synthesizing the hitherto unknown tin(IV) bis­(carboranylamidinate) Sn[*L^i^* ^Pr^]_2_. Cooling of the reaction mixture afforded a fairly large amount of well-formed, colorless crystals, which turned out to be the known solvated penta­chlorido­stannate(IV) salt [Li(THF)_4_][SnCl_5_(THF)]. This compound was first prepared and structurally characterized by Junk & Leary (2000 ▸). From the concentrated mother liquid of the penta­chlorido­stannate salt, only a small amount (*ca* 5% isolated yield) of the unexpected tin(IV) carboranylamidinate SnCl[*L^i^* ^Pr^][H*L^i^* ^Pr^] (**2**) could be obtained. The X-ray crystal structure determination of **2** revealed the presence of the first complex containing both a mono- and a dianionic carboranylamidinate ligand in one mol­ecule. As in **1**, the IR spectrum of **2** showed a characteristic *ν*(NH) band at 3410 cm^−1^. Elemental analysis and a single resonance in the ^119^Sn NMR spectrum (*δ* 290 ppm) confirmed the purity of **2**. In the mass spectrum, the mol­ecular ion was observed at *m*/*z* 692 with 47% relative intensity.

## Structural commentary   

**Figure 3:**
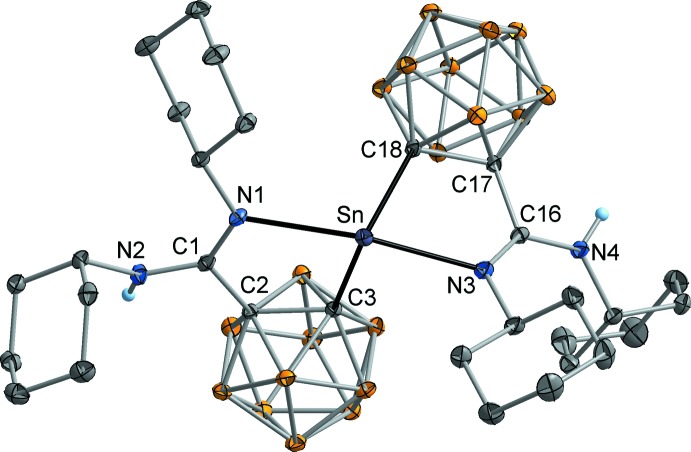
Mol­ecular structure of **1** in the crystal. Displacement ellipsoids are drawn at the 50% level, H atoms attached to C and B atoms omitted for clarity.

The molecular structures of the title compounds **1** and **2** are illustrated in Figs. 3 ▸ and 4 ▸, respectively. In both cases, two carboranylamidinate ligands are attached to one Sn atom in a typical *κC*,*κN*-chelating mode. In the tin(II) complex Sn[H*L* ^Cy^]_2_ (**1**), the coordination of the metal atom can be described as pseudo-trigonal–bipyramidal, with the two N atoms defining the axial positions [N1—Sn—N3 157.76 (4)°]. The two Sn-C bonds are situated in the equatorial plane [C3—Sn—C18 100.12 (5)°], together with the stereoactive lone pair at Sn. The bite angles of the [H*L* ^Cy^]^−^ ligands are 72.01 (5)° (N1—Sn—C3) and 73.27 (5)° (N3—Sn—C18). The coordination geometry of the Sn atom therefore resembles that in the related tin(II) bis­(carboranylamidinate) Sn[HL^*i*Pr^]_2_ [N—Sn—N 161.4 (1), C—Sn—C 97.0 (1), N—Sn—C 73.03 (7)°; Dröse *et al.*, 2010 ▸]. In **1**, the Sn—C bond lengths are very similar at 2.311 (2) Å (Sn—C3) and 2.325 (2) Å (Sn—C18), while the difference in the Sn—N bond lengths is larger [Sn—N1 2.556 (1) and Sn—N3 2.469 (1) Å]. These values are in the same range as those observed in the isopropyl substituted analogue Sn[H*L^i^* ^Pr^]_2_ [Sn—C 2.318 (2), Sn—N 2.497 (2) Å].

The tin(IV) complex SnCl[L^*i*Pr^][H*L^i^* ^Pr^] (**2**) is structurally closely related to **1**, as the Sn atom exhibits a trigonal–bipyramidal coordination with the two amidinate N atoms in the axial positions [N1—Sn—N3 176.3 (1), C2—Sn—C11 124.3 (1), N1—Sn—C2 82.9 (1), N3—Sn—C11 77.7 (1)°]. The lone pair at Sn is formally replaced by a chlorido ligand, which is almost perpendicular to the Sn—N bonds [Cl—Sn—N1 95.78 (8), Cl—Sn—N3 87.93 (7)°]. The Sn—C bond lengths are 2.160 (3) Å (Sn—C2) and 2.154 (3) Å (Sn—C11) and therefore considerably shorter than in compound **1**. This finding can be traced back to the higher oxidation number of the Sn atom. Similar Sn—C bond lengths have been observed in the related tin(IV) derivatives SnCl_3_[H*L^i^* ^Pr^] [Sn—C 2.132 (3) Å] and SnCl_2_[*L^i^* ^Pr^](THF) [2.130 (2) Å; Harmgarth *et al.*, 2017 ▸]. Since the values for protonated [H*L^i^* ^Pr^]^−^ ligands and deprotonated [*L^i^* ^Pr^]^2–^ ligands are virtually equal, the Sn—C(carborane) bond is obviously not noteworthily influenced by the bonding situation within the amidinate moiety. In contrast, in **2** the Sn—N contact to the deprotonated carboranylamidinate ligand [Sn—N1, 2.076 (3) Å] is considerably shorter than that to the protonated ligand [Sn—N3, 2.250 (3) Å]. As has been discussed previously, the *M*—N contact in [*L^i^* ^Pr^]^2–^ complexes can be regarded as a distinct single bond, while the *M*—N contact in [H*L^i^* ^Pr^]^−^ complexes should be described as a secondary coordinative inter­action (*cf*. Fig. 1 ▸, SnCl_3_[H*L^i^* ^Pr^]: Sn—N 2.255 (3) Å, SnCl_2_[*L^i^* ^Pr^](THF): Sn—N 205.0 (2) Å; Harmgarth *et al.*, 2017 ▸).

This bonding model is confirmed by the C—N bond lengths within the amidinate group. In **1**, the C—N bonds to the tin-attached N atoms [C1—N1 1.284 (2), C16—N3 1.293 (2) Å] are clearly in the range of double bonds, while the C—N bonds to the protonated N atoms [C1—N2 1.354 (2), C16—N4 1.347 (2) Å] can be regarded as single bonds. These values are consistent with those observed in other tin complexes with [H*L* ^*R*^]^−^ ligands (*e.g.* Sn[H*L* ^*i*^ ^Pr^]_2_: C=N(Sn) 1.287 (3), C—N(H) 1.347 (4) Å; Dröse *et al.*, 2010 ▸; SnCl_3_[H*L^i^* ^Pr^]: C=N(Sn) 1.288 (4), C—N(H) 1.339 (4) Å; Harmgarth *et al.* 2017 ▸). A comparable bond distribution is given in the protonated amidinate moiety in **2**, however the difference is less pronounced in this case [C12—N3 1.304 (5), C12—N4 1.328 (5) Å]. The π electron density within the amidinate group in the [*L^i^* ^Pr^]^2–^ ligand in **2** is clearly inverted, resulting in a long C—N(Sn) bond [C3—N1 1.372 (4) Å] and a short C=N bond to the non-coordinated N atom [C3—N2 1.269 (5) Å]. Similar values have been obtained in SnCl_2_[*L^i^* ^Pr^](THF) [C—N(Sn) 1.378 (3), C—N(free) 1.260 (3) Å; Harmgarth *et al.*, 2017 ▸). Besides protonation, the bonding situation within the amidinate group might be affected by the other ligands around the Sn atom, while the oxidation state of tin has apparently no influence.

In summarizing the results reported here, in tin carboranylamidinates a trigonal–bipyramidal (Sn^IV^) or pseudo-trigonal–bipyramidal (Sn^II^) coordination is highly preferred. Since the [H*L^R^*]^−^ and [*L^R^*]^2–^ ligands always display a typical *κC*,*κN*-chelating mode, this may explain why the formation of the complex SnCl[*L^i^* ^Pr^][H*L^i^* ^Pr^] (**2**) is preferred over a homoleptic complex Sn[*L^i^* ^Pr^]_2_, where the Sn atom would be tetra­hedrally coordinated. Nonetheless, the example of SnCl_2_[*L^i^* ^Pr^](THF) (Harmgarth *et al.*, 2017 ▸) demonstrates that penta-coordination of the Sn atom can also be completed by a coordinating solvent. Consequently, related bis­(carbor­anyl­amidinate) complexes Sn[*L^i^* ^Pr^]_2_(solv.) (solv. = solvent) might exist.

## Supra­molecular features   

In both **1** and **2**, the mol­ecules are well separated in the crystal and no unusually short inter­molecular contacts have been observed. The shortest intermolecular contacts are found between cyclo­hexyl groups and carborane backbones in **1** [B5⋯C14 3.727 (3) Å] and between isopropyl groups in **2** [C5⋯C15 3.670 (7) Å], respectively. In both compounds, the free N—H groups are not involved in hydrogen bonding.

## Database survey   

For reviews on amidinate complexes, see: Collins (2011 ▸)’ Edelmann (2008 ▸, 2013*a* ▸).

For reviews and articles on carboranes and their various applications, see: Belmont *et al.* (1989 ▸); Brown *et al.* (1992 ▸); Felekidis *et al.* (1997 ▸); Murphy *et al.* (1993 ▸); Teixidor *et al.* (1996 ▸); Vaillant *et al.* (2002 ▸).

For reviews on the chemistry of carboranylamidinates, see: Edelmann (2013*b* ▸); Yao & Jin (2013 ▸).

For other structurally characterized tin carboranylamidinate complexes, see: *e.g.* Cambridge Structural Database (CSD; Groom *et al.*, 2016 ▸) depository numbers 791890 (Dröse *et al.*, 2010 ▸), 963128 (Harmgarth *et al.*, 2014 ▸), 1536241–1536243 and 1536248 (Harmgarth *et al.*, 2017 ▸). For crystal structures of bis­(carboranylamidinate) complexes of other metals, see depository numbers 791889 (Cr; Dröse *et al.*, 2010 ▸), 908987–908990 (Co, Ni and Cu; Yao & Jin, 2012 ▸), 986277 and 986278 (Fe; Hillebrand *et al.*, 2014 ▸).

## Synthesis and crystallization   

All operations were performed under an argon atmosphere using standard Schlenk techniques. THF and toluene were distilled from sodium/benzo­phenone under argon. NMR spectra were recorded on a Bruker DPX400 (^1^H: 400 MHz) spectrometer in THF-*d*_8_ at 295 (2) K. ^1^H and ^13^C NMR shifts are referenced to Si(CH_3_)_4_, ^119^Sn shifts to Sn(CH_3_)_4_ (each *δ* = 0 ppm). IR spectra were measured on a Bruker Vertex V70 spectrometer equipped with a diamond ATR unit, electron impact mass spectra on a MAT95 spectrometer with an ionization energy of 70 eV. Elemental analyses (C, H and N) were performed using a VARIO EL cube apparatus.

*Preparation of Sn[HL^Cy^]_2_ (**1**): o*-Carborane (1.0 g, 7.0 mmol) in THF (50 mL) was deprotonated with *n*-butyl­lithium (7.0 mmol, 4.4 mL of a 1.6 mol l^−1^ solution in hexa­nes), followed by addition of a stoichiometric amount of the carbodi­imide CyN=C=NCy (1.44 g, 7.0 mmol). The resulting solution of Li[H*L* ^Cy^] was treated *in situ* with SnCl_2_ (0.66 g, 3.5 mmol) and then stirred for 24 h. The solvent was subsequently removed *in vacuo*, the solid residue extracted with toluene (40 mL) and the insoluble matter filtered off. The filtrate was concentrated to a total volume of *ca* 10 mL. Cooling to 278 K for 2 d afforded colorless, block-like single-crystals of **1**. Yield: 0.58 g (20%). Analysis calculated for C_30_H_66_B_20_N_4_Sn, *M* = 817.81 g mol^−1^: C 44.06, H 8.13, N 6.85%. Found: 43.66, H 8.00, N 6.25%. IR: *ν* 3423 *w* (*ν* NH), 2935 *m* (*ν* CH_2_), 2854 *m* (*ν* CH_2_), 2560 *m* (*ν* BH), 1623 *m* (*ν* C=N), 1448 *m* (*δ* CH_2_) cm^−1. 1^H NMR (400.1 MHz, THF-*D_8_*, 295 (2) K): *δ* 4.50 (*s br*, 2H, N*H*), 3.24 (*m*, 4H, C*H* Cy), 1.70–2.80 (*m br*, 20H, B*H*), 1.08–1.98 (*m*, 40H, C*H*_2_ Cy) ppm. ^13^C NMR (100.6 MHz, THF-*D_8_*, 295 (2) K): *δ* 143.0 (N*C*N), 80.3 (*C*—Sn), 77.0 (*C*—CN_2_), 54.6 (*C*H Cy), 34.8 (*C*H_2_ Cy), 25.7 (*C*H_2_ Cy), 25.4 (*C*H_2_ Cy), 25.1 (*C*H_2_ Cy), 25.0 (*C*H_2_ Cy) ppm. ^119^Sn NMR (149.2 MHz, THF-*D_8_*, 295 (2) K): *δ* −46 ppm. MS: *m*/*z* (%) 818 (27) [*M*]^+^, 467 (19) [C_15_H_32_B_10_N_2_Sn]^+^, 350 (24) [C_15_H_32_B_10_N_2_]^+^, 269 (100) [C_9_H_23_B_10_N_2_]^+^, 143 (54) [C_2_H_10_B_10_]^+^.

*Preparation of SnCl[L][HL^iPr^] (**2**):* In a similar manner as described for **1**, a solution of *o*-carborane (1.0 g, 7.0 mmol) in THF (50 mL) was treated with *n*-butyl­lithium (7.0 mmol, 4.4 mL of a 1.6 mol l^−1^ solution in hexa­nes) and ^*i*^PrN=C=N^*i*^Pr (0.88 g, 7.0 mmol). Subsequently, SnCl_4_ (0.93 g, 3.5 mmol) was added dropwise and the mixture was stirred for 24 h at r.t. Cooling of the resulting solution to 278 K for several days afforded large, colorless crystals of [Li(THF)_4_][SnCl_5_(THF)]. Yield: 2.13 g. In addition to the IR data reported by Junk & Leary (2000 ▸), the compound was further characterized by its NMR (^7^Li and ^119^Sn) data. ^7^Li NMR (155.5 MHz, THF-*D*_8_, 295 (2) K): *δ* = −0.76 ppm. ^119^Sn NMR (149.1 MHz, THF-*D*_8_, 295 (2) K): *δ* = −641 ppm. Concentration of the mother liquid to *ca.* 10 ml followed by cooling again to 278 K for several days afforded 0.13 g (5%) of **2** as colorless, plate-like single-crystals. Analysis calculated for C_18_H_49_B_20_ClN_4_Sn, *M* = 692.00 g mol^−1^: C 31.24, H 7.14, N 5.12%. Found: 31.02, H 6.88, N 4.98%. IR: *ν* 3410 *w* (*ν* NH), 2964 *m*, 2930 *w* (*ν* CH_3_, CH), 2616 *m*, 2569 *s* (*ν* BH), 1628 *m*, 1596 *vs* (*ν* C=N), 1461 *m*, 1372 *m* (*δ* CH_3_, CH) cm^−1. 1^H NMR (400.1 MHz, THF-*D_8_*, 295 (2) K): *δ* 5.56 (*s br*, 1H, N*H*), 4.33–4.39 (*m*, 2H, C*H ^i^*Pr), 3.30–3.42 (*m*, 2H, C*H ^i^*Pr), 1.7–3.2 (*m br*, 20H, B*H*), 1.23–1.56 (*m*, 14H, C*H*_3_), 0.96–1.05 (*m*, 10H, C*H*_3_) ppm. ^13^C NMR (100.6 MHz, THF-*D_8_*, 295 (2) K): *δ* 140.6 (N*C*N), 139.1 (N*C*N), 74.4 (*C*—CN_2_), 73.2 (*C*—CN_2_), 69.8 (*C*—Sn), 52.0 (*C*H ^*i*^Pr), 48.3 (*C*H ^*i*^Pr), 25.5 (*C*H_3_), 23.1 (*C*H_3_) ppm. ^119^Sn NMR (149.2 MHz, THF-*D_8_*, 295 (2) K): *δ* 290 ppm. MS: *m*/*z* (%) 692 (49) [*M*]^+^, 656 (76) [C_18_H_49_B_20_N_4_Sn]^+^, 423 (48) [C_9_H_24_B_10_ClN_2_Sn]^+^, 388 (82) [C_9_H_24_B_10_N_2_Sn]^+^, 211 (100) [C_5_H_13_B_10_N_2_]^+^, 171 (18) [C_3_H_10_B_10_N]^+^.

## Refinement   

Crystal data, data collection and structure refinement details are summarized in Table 1 ▸. H atoms attached to C atoms were fixed geometrically and refined using a riding model. The CH_3_ groups in **2** were allowed to rotate freely around the C—C vector, the corresponding C—H distances were constrained to 0.98 Å. C—H distances within CH_2_ groups were constrained to 0.99 Å, C—H distances within CH groups to 1.00 Å. H atoms attached to B and N atoms were located in the difference-Fourier map, B—H distances were restrained to 1.12 (2) Å and N—H distances to 0.88 (2) Å. The *U*_iso_(H) values were set at 1.5*U*_eq_(C) for the methyl groups in **2**, and at 1.2*U*_eq_(*X*) (*X* = B, C, N) in all other cases. For **1**, the reflections (100) and (0
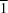
0) disagreed strongly with the structural model and were therefore omitted from the refinement.

## Supplementary Material

Crystal structure: contains datablock(s) compound_1, compound_2. DOI: 10.1107/S2056989017012671/zl2714sup1.cif

Structure factors: contains datablock(s) compound_1. DOI: 10.1107/S2056989017012671/zl2714compound_1sup2.hkl

Structure factors: contains datablock(s) compound_2. DOI: 10.1107/S2056989017012671/zl2714compound_2sup3.hkl

CCDC references: 1565456, 1565455

Additional supporting information:  crystallographic information; 3D view; checkCIF report

## Figures and Tables

**Figure 4:**
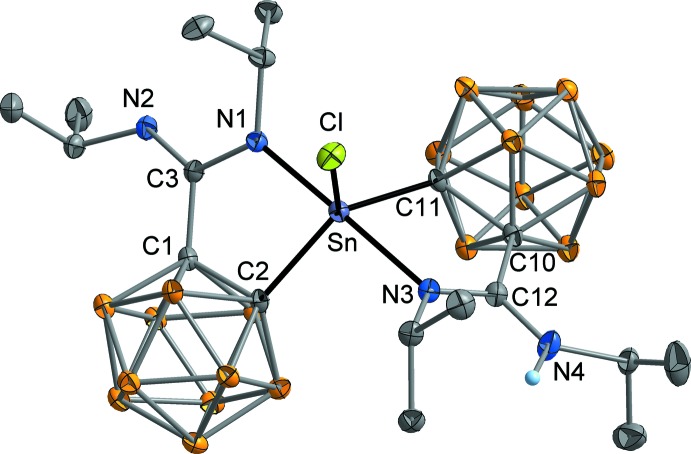
Mol­ecular structure of **2** in the crystal. Displacement ellipsoids are drawn at the 30% level, H atoms attached to C and B atoms omitted for clarity.

**Table 1:** Experimental details

	**1**	**2**
Crystal data		
Chemical formula	[Sn(C_15_H_33_B_10_N_2_)_2_]	[SnCl(C_9_H_24_B_10_N_2_)(C_9_H_25_B_10_N_2_)]
*M*_r_	817.75	691.95
Crystal system, space group	Triclinic, *P* 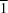	Triclinic, *P* 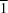
Temperature (K)	133	153
*a*, *b*, *c* (Å)	10.9097 (3), 12.9481 (3), 15.4066 (4)	11.3063 (4), 13.4631 (4), 13.7953 (4)
α, β, γ (°)	97.780 (2), 99.924 (2), 99.003 (2)	97.078 (2), 106.572 (2), 113.377 (2)
*V* (Å^3^)	2087.67 (9)	1779.52 (10)
*Z*	2	2
Radiation type	Mo *K*α	Mo *K*α
μ (mm^−1^)	0.64	0.81
Crystal size (mm)	0.29 × 0.27 × 0.26	0.34 × 0.23 × 0.04

Data collection		
Diffractometer	Stoe *IPDS* 2T	Stoe *IPDS* 2T
Absorption correction	–	Numerical *X-AREA* and *X-RED* (Stoe & Cie, 2002 ▸)
*T*_min_, *T*_max_	–	0.832, 0.964
No. of measured, independent and observed [*I* > 2σ(*I*)] reflections	23428, 9102, 8436	15500, 6943, 5878
*R*_int_	0.031	0.055
(sin θ/λ)_max_ (Å^−1^)	0.639	0.617

Refinement		
*R*[*F* ^2^ > 2σ(*F* ^2^)], *wR*(*F* ^2^), *S*	0.022, 0.058, 1.05	0.039, 0.092, 1.02
No. of reflections	9102	6943
No. of parameters	562	469
No. of restraints	21	21
H-atom treatment	H atoms treated by a mixture of independent and constrained refinement	H atoms treated by a mixture of independent and constrained refinement
Δρ_max_, Δρ_min_ (e Å^−3^)	0.48, −0.62	0.84, −1.17

Computer programs: *X-AREA* and *X-RED* (Stoe & Cie, 2002 ▸), *SIR97* (Altomare *et al.*, 1999 ▸), *SHELXL2016/4* (Sheldrick, 2015 ▸), *DIAMOND* (Brandenburg, 1999 ▸) and *publCIF* (Westrip, 2010 ▸).

## supplementary crystallographic information

### Bis(N,N'-dicyclohexylamidinatocarboranate)tin(II) (compound_1) Crystal data

**Table:**

[Sn(C_15_H_33_B_10_N_2_)_2_]	*Z* = 2
*M**_r_* = 817.75	*F*(000) = 848
Triclinic, *P*1	*D*_x_ = 1.301 Mg m^−^^3^
*a* = 10.9097 (3) Å	Mo *K*α radiation, λ = 0.71073 Å
*b* = 12.9481 (3) Å	Cell parameters from 28532 reflections
*c* = 15.4066 (4) Å	θ = 1.9–29.2°
α = 97.780 (2)°	µ = 0.64 mm^−^^1^
β = 99.924 (2)°	*T* = 133 K
γ = 99.003 (2)°	Block, colorless
*V* = 2087.67 (9) Å^3^	0.29 × 0.27 × 0.26 mm

### Bis(N,N'-dicyclohexylamidinatocarboranate)tin(II) (compound_1) Data collection

**Table:**

Stoe IPDS 2T diffractometer	8436 reflections with *I* > 2σ(*I*)
Radiation source: fine-focus sealed tube	*R*_int_ = 0.031
Detector resolution: 6.67 pixels mm^-1^	θ_max_ = 27.0°, θ_min_ = 2.1°
area detector scans	*h* = −13→13
23428 measured reflections	*k* = −16→16
9102 independent reflections	*l* = −16→19

### Bis(N,N'-dicyclohexylamidinatocarboranate)tin(II) (compound_1) Refinement

**Table:**

Refinement on *F*^2^	Primary atom site location: heavy-atom method
Least-squares matrix: full	Secondary atom site location: difference Fourier map
*R*[*F*^2^ > 2σ(*F*^2^)] = 0.022	Hydrogen site location: mixed
*wR*(*F*^2^) = 0.058	H atoms treated by a mixture of independent and constrained refinement
*S* = 1.05	*w* = 1/[σ^2^(*F*_o_^2^) + (0.0298*P*)^2^ + 0.9924*P*] where *P* = (*F*_o_^2^ + 2*F*_c_^2^)/3
9102 reflections	(Δ/σ)_max_ = 0.002
562 parameters	Δρ_max_ = 0.48 e Å^−^^3^
21 restraints	Δρ_min_ = −0.62 e Å^−^^3^

### Bis(N,N'-dicyclohexylamidinatocarboranate)tin(II) (compound_1) Special details

**Table:**

Geometry. All esds (except the esd in the dihedral angle between two l.s. planes) are estimated using the full covariance matrix. The cell esds are taken into account individually in the estimation of esds in distances, angles and torsion angles; correlations between esds in cell parameters are only used when they are defined by crystal symmetry. An approximate (isotropic) treatment of cell esds is used for estimating esds involving l.s. planes.

### Bis(N,N'-dicyclohexylamidinatocarboranate)tin(II) (compound_1) Fractional atomic coordinates and isotropic or equivalent isotropic displacement parameters (Å^2^)

**Table:**

	*x*	*y*	*z*	*U*_iso_*/*U*_eq_	
C1	−0.11416 (14)	0.11380 (12)	0.72392 (10)	0.0125 (3)	
C2	0.00983 (13)	0.13613 (11)	0.79178 (10)	0.0112 (3)	
C3	0.10797 (14)	0.24815 (12)	0.79304 (10)	0.0125 (3)	
C4	−0.22894 (14)	0.14758 (12)	0.58599 (10)	0.0133 (3)	
H12	−0.310449	0.125827	0.605684	0.016*	
C5	−0.20260 (15)	0.05378 (12)	0.52480 (11)	0.0168 (3)	
H14	−0.115600	0.070897	0.513662	0.020*	
H13	−0.207704	−0.009033	0.555103	0.020*	
C6	−0.29621 (16)	0.02717 (13)	0.43577 (11)	0.0196 (3)	
H16	−0.275133	−0.032624	0.397421	0.024*	
H15	−0.382969	0.005452	0.446031	0.024*	
C7	−0.29039 (16)	0.12377 (14)	0.38940 (11)	0.0215 (3)	
H17	−0.349829	0.106237	0.330952	0.026*	
H18	−0.203871	0.144937	0.378525	0.026*	
C8	−0.32548 (17)	0.21514 (14)	0.44765 (11)	0.0222 (3)	
H19	−0.413889	0.195084	0.455241	0.027*	
H20	−0.320347	0.278056	0.417470	0.027*	
C9	−0.23742 (15)	0.24343 (12)	0.53957 (11)	0.0171 (3)	
H22	−0.151709	0.274929	0.532519	0.020*	
H21	−0.268630	0.297413	0.577592	0.020*	
C10	−0.33271 (14)	−0.00110 (12)	0.71104 (10)	0.0146 (3)	
H23	−0.358930	−0.009969	0.644590	0.018*	
C11	−0.39596 (15)	0.08449 (13)	0.75406 (11)	0.0175 (3)	
H24	−0.360476	0.153926	0.739810	0.021*	
H25	−0.487759	0.068625	0.728160	0.021*	
C12	−0.37712 (16)	0.09195 (14)	0.85542 (12)	0.0213 (3)	
H26	−0.286484	0.118080	0.882330	0.026*	
H27	−0.425951	0.143678	0.879282	0.026*	
C13	−0.41987 (16)	−0.01526 (14)	0.88186 (12)	0.0216 (3)	
H28	−0.401460	−0.008350	0.947806	0.026*	
H29	−0.512506	−0.037551	0.860864	0.026*	
C14	−0.35329 (15)	−0.09960 (13)	0.84209 (11)	0.0182 (3)	
H31	−0.387844	−0.169057	0.856731	0.022*	
H30	−0.261846	−0.081933	0.869049	0.022*	
C15	−0.37045 (15)	−0.10775 (12)	0.74081 (11)	0.0167 (3)	
H32	−0.460260	−0.137147	0.713454	0.020*	
H33	−0.318685	−0.157852	0.718408	0.020*	
C16	0.31859 (14)	0.47432 (11)	0.70647 (10)	0.0130 (3)	
C17	0.28318 (13)	0.41073 (12)	0.61218 (10)	0.0119 (3)	
C18	0.14315 (14)	0.33148 (12)	0.58673 (10)	0.0128 (3)	
C19	0.23568 (15)	0.57196 (12)	0.82159 (10)	0.0152 (3)	
H45	0.307927	0.570908	0.870980	0.018*	
C20	0.25677 (17)	0.67761 (13)	0.78688 (12)	0.0212 (3)	
H46	0.187093	0.677226	0.736231	0.025*	
H47	0.337108	0.685802	0.764696	0.025*	
C21	0.26204 (19)	0.77176 (14)	0.86052 (14)	0.0291 (4)	
H49	0.336564	0.776213	0.908651	0.035*	
H48	0.271895	0.838370	0.835450	0.035*	
C22	0.14225 (19)	0.75993 (14)	0.89946 (14)	0.0285 (4)	
H51	0.151005	0.818350	0.950014	0.034*	
H50	0.069026	0.764782	0.853290	0.034*	
C23	0.11870 (17)	0.65455 (14)	0.93165 (12)	0.0228 (3)	
H52	0.037756	0.646504	0.953041	0.027*	
H53	0.187319	0.653076	0.982465	0.027*	
C24	0.11324 (15)	0.56241 (13)	0.85708 (11)	0.0178 (3)	
H55	0.098409	0.494724	0.880134	0.021*	
H54	0.041503	0.561438	0.807726	0.021*	
C25	0.51973 (14)	0.56385 (13)	0.81933 (10)	0.0161 (3)	
H56	0.482231	0.623151	0.846298	0.019*	
C26	0.65291 (15)	0.60854 (13)	0.80871 (11)	0.0193 (3)	
H58	0.699643	0.652674	0.865881	0.023*	
H57	0.647321	0.655075	0.762754	0.023*	
C27	0.72730 (16)	0.52297 (16)	0.78193 (12)	0.0243 (4)	
H59	0.687323	0.484621	0.721111	0.029*	
H60	0.814776	0.556632	0.780608	0.029*	
C28	0.73118 (18)	0.44483 (17)	0.84694 (14)	0.0308 (4)	
H62	0.778348	0.481726	0.906646	0.037*	
H61	0.775950	0.388121	0.826440	0.037*	
C29	0.59738 (18)	0.39607 (15)	0.85348 (14)	0.0284 (4)	
H64	0.601467	0.347390	0.897785	0.034*	
H63	0.552882	0.354124	0.794849	0.034*	
C30	0.52318 (16)	0.48122 (14)	0.88119 (11)	0.0216 (3)	
H66	0.562167	0.517180	0.942888	0.026*	
H65	0.435406	0.447070	0.881203	0.026*	
B1	0.14297 (16)	0.12971 (14)	0.74892 (12)	0.0145 (3)	
H2	0.1300 (18)	0.1113 (15)	0.6776 (10)	0.017*	
B2	0.08805 (16)	0.03507 (14)	0.81413 (12)	0.0150 (3)	
H3	0.0439 (18)	−0.0445 (12)	0.7803 (13)	0.018*	
B3	0.01701 (16)	0.10089 (14)	0.89549 (12)	0.0151 (3)	
H4	−0.0698 (15)	0.0625 (15)	0.9116 (13)	0.018*	
B4	0.02671 (16)	0.23545 (14)	0.87955 (11)	0.0148 (3)	
H5	−0.0512 (16)	0.2773 (15)	0.8812 (13)	0.018*	
B5	0.17205 (17)	0.07622 (14)	0.92563 (12)	0.0169 (3)	
H6	0.1871 (19)	0.0167 (14)	0.9662 (13)	0.020*	
B6	0.13306 (16)	0.20070 (15)	0.96705 (12)	0.0164 (3)	
H7	0.1257 (19)	0.2231 (16)	1.0363 (10)	0.020*	
B7	0.26059 (16)	0.22856 (14)	0.82147 (12)	0.0157 (3)	
H8	0.3340 (16)	0.2737 (15)	0.7953 (13)	0.019*	
B8	0.25023 (16)	0.09419 (14)	0.83482 (12)	0.0167 (3)	
H9	0.3217 (17)	0.0484 (15)	0.8160 (14)	0.020*	
B9	0.18873 (16)	0.29350 (14)	0.90120 (12)	0.0155 (3)	
H10	0.2141 (18)	0.3779 (12)	0.9235 (13)	0.019*	
B10	0.27885 (16)	0.19592 (14)	0.93032 (12)	0.0161 (3)	
H11	0.3663 (19)	0.2203 (16)	0.9762 (14)	0.019*	
B11	0.27956 (16)	0.27721 (13)	0.59623 (12)	0.0137 (3)	
H35	0.3081 (18)	0.2402 (15)	0.6538 (11)	0.016*	
B12	0.39067 (16)	0.36406 (14)	0.55529 (12)	0.0141 (3)	
H36	0.4881 (14)	0.3792 (15)	0.5892 (12)	0.017*	
B13	0.16204 (16)	0.44953 (14)	0.54558 (12)	0.0152 (3)	
H37	0.1211 (18)	0.5161 (14)	0.5738 (13)	0.018*	
B14	0.14878 (17)	0.22590 (14)	0.50799 (12)	0.0159 (3)	
H38	0.0918 (17)	0.1500 (13)	0.5099 (14)	0.019*	
B15	0.07798 (17)	0.33208 (15)	0.47631 (12)	0.0165 (3)	
H39	−0.0239 (14)	0.3241 (16)	0.4545 (13)	0.020*	
B16	0.30530 (17)	0.24568 (14)	0.48624 (12)	0.0159 (3)	
H40	0.3525 (18)	0.1793 (14)	0.4716 (14)	0.019*	
B17	0.31896 (17)	0.47129 (14)	0.52457 (12)	0.0159 (3)	
H41	0.3735 (17)	0.5515 (12)	0.5392 (13)	0.019*	
B18	0.32931 (17)	0.36563 (15)	0.44173 (12)	0.0166 (3)	
H42	0.3941 (17)	0.3771 (16)	0.3954 (12)	0.020*	
B19	0.18760 (18)	0.41939 (15)	0.43589 (12)	0.0183 (3)	
H43	0.1547 (19)	0.4634 (15)	0.3853 (12)	0.022*	
B20	0.17989 (17)	0.27947 (15)	0.41158 (12)	0.0182 (3)	
H44	0.1438 (19)	0.2332 (15)	0.3446 (11)	0.022*	
N1	−0.12440 (12)	0.17515 (10)	0.66519 (9)	0.0136 (2)	
N2	−0.19351 (12)	0.02767 (11)	0.73572 (9)	0.0149 (3)	
H1	−0.1636 (18)	−0.0031 (15)	0.7740 (12)	0.018*	
N3	0.22665 (12)	0.48252 (10)	0.74832 (9)	0.0138 (2)	
N4	0.44228 (12)	0.51868 (11)	0.73005 (9)	0.0152 (3)	
H34	0.4835 (18)	0.5085 (16)	0.6891 (12)	0.018*	
SN	0.02544 (2)	0.35454 (2)	0.69779 (2)	0.01185 (4)	

### Bis(N,N'-dicyclohexylamidinatocarboranate)tin(II) (compound_1) Atomic displacement parameters (Å^2^)

**Table:**

	*U*^11^	*U*^22^	*U*^33^	*U*^12^	*U*^13^	*U*^23^
C1	0.0101 (6)	0.0134 (7)	0.0127 (7)	0.0025 (5)	0.0011 (5)	−0.0007 (5)
C2	0.0102 (6)	0.0132 (6)	0.0103 (7)	0.0019 (5)	0.0017 (5)	0.0024 (5)
C3	0.0114 (7)	0.0132 (7)	0.0122 (7)	0.0007 (5)	0.0014 (5)	0.0025 (5)
C4	0.0120 (7)	0.0144 (7)	0.0119 (7)	0.0015 (5)	−0.0002 (5)	0.0010 (6)
C5	0.0167 (7)	0.0170 (7)	0.0149 (7)	0.0049 (6)	−0.0007 (6)	−0.0004 (6)
C6	0.0184 (8)	0.0214 (8)	0.0157 (8)	0.0045 (6)	−0.0020 (6)	−0.0030 (6)
C7	0.0210 (8)	0.0284 (9)	0.0130 (7)	0.0044 (7)	−0.0015 (6)	0.0023 (6)
C8	0.0239 (8)	0.0226 (8)	0.0178 (8)	0.0080 (7)	−0.0058 (6)	0.0031 (6)
C9	0.0177 (7)	0.0156 (7)	0.0160 (7)	0.0030 (6)	−0.0022 (6)	0.0033 (6)
C10	0.0103 (7)	0.0174 (7)	0.0142 (7)	0.0002 (5)	−0.0004 (5)	0.0022 (6)
C11	0.0128 (7)	0.0187 (7)	0.0213 (8)	0.0038 (6)	0.0025 (6)	0.0043 (6)
C12	0.0192 (8)	0.0239 (8)	0.0211 (8)	0.0071 (6)	0.0044 (6)	−0.0001 (7)
C13	0.0176 (8)	0.0309 (9)	0.0190 (8)	0.0082 (7)	0.0055 (6)	0.0064 (7)
C14	0.0161 (7)	0.0208 (8)	0.0188 (8)	0.0036 (6)	0.0031 (6)	0.0071 (6)
C15	0.0150 (7)	0.0163 (7)	0.0168 (8)	−0.0002 (6)	0.0008 (6)	0.0017 (6)
C16	0.0143 (7)	0.0116 (6)	0.0126 (7)	0.0030 (5)	0.0015 (5)	0.0010 (5)
C17	0.0097 (6)	0.0134 (7)	0.0125 (7)	0.0018 (5)	0.0031 (5)	0.0010 (5)
C18	0.0100 (6)	0.0140 (7)	0.0133 (7)	0.0010 (5)	0.0022 (5)	0.0003 (5)
C19	0.0150 (7)	0.0155 (7)	0.0149 (7)	0.0037 (6)	0.0048 (6)	−0.0018 (6)
C20	0.0238 (8)	0.0181 (8)	0.0234 (8)	0.0039 (6)	0.0101 (7)	0.0026 (7)
C21	0.0334 (10)	0.0150 (8)	0.0389 (11)	0.0013 (7)	0.0147 (8)	−0.0015 (7)
C22	0.0353 (10)	0.0179 (8)	0.0342 (10)	0.0084 (7)	0.0152 (8)	−0.0037 (7)
C23	0.0265 (9)	0.0219 (8)	0.0218 (8)	0.0071 (7)	0.0113 (7)	−0.0010 (7)
C24	0.0167 (7)	0.0160 (7)	0.0211 (8)	0.0041 (6)	0.0071 (6)	−0.0008 (6)
C25	0.0130 (7)	0.0197 (7)	0.0130 (7)	0.0006 (6)	0.0012 (6)	−0.0025 (6)
C26	0.0141 (7)	0.0230 (8)	0.0173 (8)	−0.0034 (6)	0.0004 (6)	0.0022 (6)
C27	0.0135 (7)	0.0385 (10)	0.0217 (8)	0.0049 (7)	0.0048 (6)	0.0059 (7)
C28	0.0200 (9)	0.0397 (11)	0.0362 (11)	0.0119 (8)	0.0037 (8)	0.0134 (9)
C29	0.0250 (9)	0.0282 (9)	0.0342 (10)	0.0064 (7)	0.0036 (8)	0.0145 (8)
C30	0.0171 (8)	0.0302 (9)	0.0162 (8)	−0.0008 (7)	0.0033 (6)	0.0057 (7)
B1	0.0117 (7)	0.0170 (8)	0.0149 (8)	0.0029 (6)	0.0032 (6)	0.0022 (6)
B2	0.0134 (8)	0.0157 (8)	0.0165 (8)	0.0042 (6)	0.0019 (6)	0.0040 (6)
B3	0.0135 (8)	0.0197 (8)	0.0121 (8)	0.0015 (6)	0.0016 (6)	0.0056 (6)
B4	0.0135 (8)	0.0191 (8)	0.0109 (8)	0.0033 (6)	0.0018 (6)	0.0001 (6)
B5	0.0145 (8)	0.0191 (8)	0.0171 (8)	0.0029 (6)	0.0001 (6)	0.0071 (7)
B6	0.0134 (8)	0.0229 (9)	0.0119 (8)	0.0028 (7)	0.0005 (6)	0.0024 (7)
B7	0.0113 (7)	0.0199 (8)	0.0152 (8)	0.0012 (6)	0.0014 (6)	0.0035 (7)
B8	0.0125 (8)	0.0199 (8)	0.0180 (8)	0.0051 (6)	0.0017 (6)	0.0037 (7)
B9	0.0139 (8)	0.0173 (8)	0.0131 (8)	0.0018 (6)	−0.0012 (6)	0.0009 (6)
B10	0.0115 (8)	0.0203 (8)	0.0152 (8)	0.0018 (6)	−0.0005 (6)	0.0037 (7)
B11	0.0136 (8)	0.0129 (7)	0.0154 (8)	0.0029 (6)	0.0050 (6)	0.0013 (6)
B12	0.0128 (8)	0.0160 (8)	0.0142 (8)	0.0028 (6)	0.0060 (6)	0.0005 (6)
B13	0.0135 (8)	0.0175 (8)	0.0153 (8)	0.0042 (6)	0.0026 (6)	0.0046 (6)
B14	0.0154 (8)	0.0156 (8)	0.0156 (8)	0.0009 (6)	0.0054 (6)	−0.0021 (6)
B15	0.0137 (8)	0.0219 (8)	0.0128 (8)	0.0029 (7)	0.0016 (6)	0.0002 (7)
B16	0.0155 (8)	0.0165 (8)	0.0154 (8)	0.0018 (6)	0.0064 (6)	−0.0016 (6)
B17	0.0158 (8)	0.0169 (8)	0.0164 (8)	0.0025 (6)	0.0063 (6)	0.0045 (7)
B18	0.0161 (8)	0.0199 (8)	0.0136 (8)	0.0016 (7)	0.0052 (6)	0.0017 (7)
B19	0.0181 (8)	0.0240 (9)	0.0138 (8)	0.0042 (7)	0.0042 (7)	0.0055 (7)
B20	0.0157 (8)	0.0245 (9)	0.0127 (8)	0.0016 (7)	0.0036 (6)	−0.0010 (7)
N1	0.0122 (6)	0.0147 (6)	0.0120 (6)	0.0021 (5)	−0.0012 (5)	0.0007 (5)
N2	0.0098 (6)	0.0178 (6)	0.0164 (6)	0.0012 (5)	−0.0007 (5)	0.0058 (5)
N3	0.0135 (6)	0.0137 (6)	0.0140 (6)	0.0032 (5)	0.0036 (5)	−0.0004 (5)
N4	0.0111 (6)	0.0209 (6)	0.0120 (6)	0.0008 (5)	0.0025 (5)	−0.0006 (5)
SN	0.01086 (5)	0.01295 (5)	0.01233 (5)	0.00372 (4)	0.00290 (4)	0.00161 (4)

### Bis(N,N'-dicyclohexylamidinatocarboranate)tin(II) (compound_1) Geometric parameters (Å, º)

**Table:**

C1—N1	1.284 (2)	C25—C30	1.527 (2)
C1—N2	1.354 (2)	C25—H56	1.0000
C1—C2	1.521 (2)	C26—C27	1.526 (2)
C2—C3	1.658 (2)	C26—H58	0.9900
C2—B4	1.699 (2)	C26—H57	0.9900
C2—B1	1.705 (2)	C27—C28	1.517 (3)
C2—B2	1.711 (2)	C27—H59	0.9900
C2—B3	1.712 (2)	C27—H60	0.9900
C3—B7	1.715 (2)	C28—C29	1.523 (3)
C3—B1	1.718 (2)	C28—H62	0.9900
C3—B9	1.722 (2)	C28—H61	0.9900
C3—B4	1.735 (2)	C29—C30	1.524 (3)
C3—SN	2.3112 (15)	C29—H64	0.9900
C4—N1	1.4805 (19)	C29—H63	0.9900
C4—C9	1.521 (2)	C30—H66	0.9900
C4—C5	1.529 (2)	C30—H65	0.9900
C4—H12	1.0000	B1—B8	1.770 (2)
C5—C6	1.524 (2)	B1—B7	1.772 (3)
C5—H14	0.9900	B1—B2	1.784 (3)
C5—H13	0.9900	B1—H2	1.072 (14)
C6—C7	1.520 (2)	B2—B5	1.770 (3)
C6—H16	0.9900	B2—B8	1.770 (2)
C6—H15	0.9900	B2—B3	1.775 (2)
C7—C8	1.525 (2)	B2—H3	1.085 (14)
C7—H17	0.9900	B3—B6	1.763 (3)
C7—H18	0.9900	B3—B5	1.766 (2)
C8—C9	1.531 (2)	B3—B4	1.782 (3)
C8—H19	0.9900	B3—H4	1.083 (14)
C8—H20	0.9900	B4—B9	1.765 (2)
C9—H22	0.9900	B4—B6	1.776 (2)
C9—H21	0.9900	B4—H5	1.079 (14)
C10—N2	1.4749 (19)	B5—B10	1.772 (3)
C10—C11	1.530 (2)	B5—B8	1.782 (3)
C10—C15	1.531 (2)	B5—B6	1.793 (3)
C10—H23	1.0000	B5—H6	1.070 (15)
C11—C12	1.527 (2)	B6—B9	1.776 (3)
C11—H24	0.9900	B6—B10	1.785 (2)
C11—H25	0.9900	B6—H7	1.087 (15)
C12—C13	1.525 (2)	B7—B9	1.758 (3)
C12—H26	0.9900	B7—B8	1.768 (3)
C12—H27	0.9900	B7—B10	1.770 (3)
C13—C14	1.522 (2)	B7—H8	1.083 (15)
C13—H28	0.9900	B8—B10	1.785 (3)
C13—H29	0.9900	B8—H9	1.105 (14)
C14—C15	1.526 (2)	B9—B10	1.776 (3)
C14—H31	0.9900	B9—H10	1.077 (15)
C14—H30	0.9900	B10—H11	1.06 (2)
C15—H32	0.9900	B11—B14	1.765 (2)
C15—H33	0.9900	B11—B16	1.769 (2)
C16—N3	1.293 (2)	B11—B12	1.778 (2)
C16—N4	1.347 (2)	B11—H35	1.087 (14)
C16—C17	1.523 (2)	B12—B16	1.766 (2)
C17—C18	1.654 (2)	B12—B18	1.766 (3)
C17—B11	1.706 (2)	B12—B17	1.776 (2)
C17—B13	1.712 (2)	B12—H36	1.075 (14)
C17—B12	1.715 (2)	B13—B15	1.755 (3)
C17—B17	1.721 (2)	B13—B19	1.762 (3)
C18—B14	1.717 (2)	B13—B17	1.783 (2)
C18—B15	1.729 (2)	B13—H37	1.103 (14)
C18—B13	1.729 (2)	B14—B15	1.763 (3)
C18—B11	1.735 (2)	B14—B16	1.783 (2)
C18—SN	2.3249 (15)	B14—B20	1.784 (3)
C19—N3	1.4822 (19)	B14—H38	1.084 (15)
C19—C24	1.522 (2)	B15—B19	1.766 (3)
C19—C20	1.534 (2)	B15—B20	1.769 (3)
C19—H45	1.0000	B15—H39	1.088 (15)
C20—C21	1.536 (2)	B16—B20	1.780 (3)
C20—H46	0.9900	B16—B18	1.782 (3)
C20—H47	0.9900	B16—H40	1.087 (14)
C21—C22	1.524 (3)	B17—B18	1.772 (3)
C21—H49	0.9900	B17—B19	1.774 (3)
C21—H48	0.9900	B17—H41	1.086 (15)
C22—C23	1.514 (3)	B18—B20	1.774 (3)
C22—H51	0.9900	B18—B19	1.786 (3)
C22—H50	0.9900	B18—H42	1.096 (15)
C23—C24	1.526 (2)	B19—B20	1.785 (3)
C23—H52	0.9900	B19—H43	1.072 (15)
C23—H53	0.9900	B20—H44	1.094 (15)
C24—H55	0.9900	N1—SN	2.5560 (13)
C24—H54	0.9900	N2—H1	0.807 (15)
C25—N4	1.4747 (19)	N3—SN	2.4686 (13)
C25—C26	1.522 (2)	N4—H34	0.843 (15)
			
N1—C1—N2	131.54 (14)	B6—B4—B3	59.43 (10)
N1—C1—C2	116.65 (13)	C2—B4—H5	116.7 (11)
N2—C1—C2	111.77 (13)	C3—B4—H5	117.1 (11)
C1—C2—C3	116.58 (12)	B9—B4—H5	126.1 (11)
C1—C2—B4	116.93 (12)	B6—B4—H5	131.1 (11)
C3—C2—B4	62.23 (9)	B3—B4—H5	123.0 (11)
C1—C2—B1	115.88 (12)	B3—B5—B2	60.27 (10)
C3—C2—B1	61.41 (9)	B3—B5—B10	107.65 (12)
B4—C2—B1	114.69 (12)	B2—B5—B10	108.14 (12)
C1—C2—B2	120.26 (12)	B3—B5—B8	107.84 (12)
C3—C2—B2	112.43 (11)	B2—B5—B8	59.79 (10)
B4—C2—B2	114.90 (12)	B10—B5—B8	60.29 (10)
B1—C2—B2	62.94 (10)	B3—B5—B6	59.40 (10)
C1—C2—B3	121.28 (12)	B2—B5—B6	107.87 (12)
C3—C2—B3	112.70 (11)	B10—B5—B6	60.07 (10)
B4—C2—B3	62.97 (10)	B8—B5—B6	108.07 (12)
B1—C2—B3	114.54 (12)	B3—B5—H6	118.6 (11)
B2—C2—B3	62.45 (10)	B2—B5—H6	118.3 (11)
C2—C3—B7	108.79 (11)	B10—B5—H6	125.8 (11)
C2—C3—B1	60.63 (9)	B8—B5—H6	122.9 (11)
B7—C3—B1	62.15 (10)	B6—B5—H6	123.1 (11)
C2—C3—B9	108.15 (11)	B3—B6—B4	60.44 (10)
B7—C3—B9	61.52 (10)	B3—B6—B9	107.58 (12)
B1—C3—B9	112.39 (12)	B4—B6—B9	59.56 (10)
C2—C3—B4	60.03 (9)	B3—B6—B10	107.19 (12)
B7—C3—B4	111.76 (11)	B4—B6—B10	107.52 (12)
B1—C3—B4	112.18 (12)	B9—B6—B10	59.83 (10)
B9—C3—B4	61.38 (10)	B3—B6—B5	59.52 (10)
C2—C3—SN	113.65 (9)	B4—B6—B5	107.64 (12)
B7—C3—SN	129.66 (10)	B9—B6—B5	107.05 (12)
B1—C3—SN	119.32 (10)	B10—B6—B5	59.38 (10)
B9—C3—SN	124.42 (10)	B3—B6—H7	120.1 (11)
B4—C3—SN	112.20 (9)	B4—B6—H7	120.3 (11)
N1—C4—C9	109.78 (12)	B9—B6—H7	123.1 (11)
N1—C4—C5	107.60 (12)	B10—B6—H7	124.2 (11)
C9—C4—C5	112.01 (13)	B5—B6—H7	122.6 (11)
N1—C4—H12	109.1	C3—B7—B9	59.45 (9)
C9—C4—H12	109.1	C3—B7—B8	106.32 (12)
C5—C4—H12	109.1	B9—B7—B8	108.83 (13)
C6—C5—C4	111.97 (13)	C3—B7—B10	106.82 (12)
C6—C5—H14	109.2	B9—B7—B10	60.46 (10)
C4—C5—H14	109.2	B8—B7—B10	60.60 (10)
C6—C5—H13	109.2	C3—B7—B1	59.01 (9)
C4—C5—H13	109.2	B9—B7—B1	108.16 (12)
H14—C5—H13	107.9	B8—B7—B1	60.00 (10)
C7—C6—C5	109.56 (14)	B10—B7—B1	108.57 (12)
C7—C6—H16	109.8	C3—B7—H8	118.8 (11)
C5—C6—H16	109.8	B9—B7—H8	119.6 (11)
C7—C6—H15	109.8	B8—B7—H8	125.4 (11)
C5—C6—H15	109.8	B10—B7—H8	125.1 (11)
H16—C6—H15	108.2	B1—B7—H8	120.1 (11)
C6—C7—C8	109.74 (14)	B7—B8—B1	60.11 (10)
C6—C7—H17	109.7	B7—B8—B2	108.13 (12)
C8—C7—H17	109.7	B1—B8—B2	60.51 (10)
C6—C7—H18	109.7	B7—B8—B5	107.47 (12)
C8—C7—H18	109.7	B1—B8—B5	108.16 (12)
H17—C7—H18	108.2	B2—B8—B5	59.75 (10)
C7—C8—C9	111.47 (13)	B7—B8—B10	59.77 (10)
C7—C8—H19	109.3	B1—B8—B10	108.00 (12)
C9—C8—H19	109.3	B2—B8—B10	107.54 (12)
C7—C8—H20	109.3	B5—B8—B10	59.57 (10)
C9—C8—H20	109.3	B7—B8—H9	120.8 (11)
H19—C8—H20	108.0	B1—B8—H9	118.6 (11)
C4—C9—C8	112.47 (13)	B2—B8—H9	120.9 (11)
C4—C9—H22	109.1	B5—B8—H9	124.4 (11)
C8—C9—H22	109.1	B10—B8—H9	124.2 (11)
C4—C9—H21	109.1	C3—B9—B7	59.03 (9)
C8—C9—H21	109.1	C3—B9—B4	59.67 (9)
H22—C9—H21	107.8	B7—B9—B4	108.35 (12)
N2—C10—C11	111.43 (13)	C3—B9—B10	106.23 (12)
N2—C10—C15	106.94 (12)	B7—B9—B10	60.12 (10)
C11—C10—C15	111.62 (13)	B4—B9—B10	108.41 (13)
N2—C10—H23	108.9	C3—B9—B6	106.77 (12)
C11—C10—H23	108.9	B7—B9—B6	108.57 (12)
C15—C10—H23	108.9	B4—B9—B6	60.21 (10)
C12—C11—C10	112.50 (13)	B10—B9—B6	60.32 (10)
C12—C11—H24	109.1	C3—B9—H10	118.2 (11)
C10—C11—H24	109.1	B7—B9—H10	121.5 (11)
C12—C11—H25	109.1	B4—B9—H10	117.7 (11)
C10—C11—H25	109.1	B10—B9—H10	127.2 (11)
H24—C11—H25	107.8	B6—B9—H10	124.5 (11)
C13—C12—C11	111.59 (14)	B7—B10—B5	107.81 (12)
C13—C12—H26	109.3	B7—B10—B9	59.42 (10)
C11—C12—H26	109.3	B5—B10—B9	107.99 (12)
C13—C12—H27	109.3	B7—B10—B6	107.64 (12)
C11—C12—H27	109.3	B5—B10—B6	60.55 (10)
H26—C12—H27	108.0	B9—B10—B6	59.85 (10)
C14—C13—C12	111.49 (14)	B7—B10—B8	59.63 (10)
C14—C13—H28	109.3	B5—B10—B8	60.14 (10)
C12—C13—H28	109.3	B9—B10—B8	107.24 (12)
C14—C13—H29	109.3	B6—B10—B8	108.33 (12)
C12—C13—H29	109.3	B7—B10—H11	121.0 (11)
H28—C13—H29	108.0	B5—B10—H11	124.3 (11)
C13—C14—C15	111.51 (13)	B9—B10—H11	118.8 (11)
C13—C14—H31	109.3	B6—B10—H11	120.4 (11)
C15—C14—H31	109.3	B8—B10—H11	124.5 (11)
C13—C14—H30	109.3	C17—B11—C18	57.43 (9)
C15—C14—H30	109.3	C17—B11—B14	104.20 (12)
H31—C14—H30	108.0	C18—B11—B14	58.74 (9)
C14—C15—C10	113.21 (13)	C17—B11—B16	104.87 (12)
C14—C15—H32	108.9	C18—B11—B16	106.21 (12)
C10—C15—H32	108.9	B14—B11—B16	60.58 (10)
C14—C15—H33	108.9	C17—B11—B12	58.93 (9)
C10—C15—H33	108.9	C18—B11—B12	105.83 (12)
H32—C15—H33	107.7	B14—B11—B12	107.87 (12)
N3—C16—N4	130.59 (14)	B16—B11—B12	59.72 (10)
N3—C16—C17	116.38 (13)	C17—B11—H35	118.7 (10)
N4—C16—C17	112.89 (13)	C18—B11—H35	119.7 (10)
C16—C17—C18	115.68 (12)	B14—B11—H35	125.9 (11)
C16—C17—B11	119.82 (12)	B16—B11—H35	128.4 (10)
C18—C17—B11	62.16 (9)	B12—B11—H35	121.6 (10)
C16—C17—B13	113.33 (12)	C17—B12—B16	104.63 (11)
C18—C17—B13	61.79 (9)	C17—B12—B18	104.94 (12)
B11—C17—B13	114.36 (12)	B16—B12—B18	60.58 (10)
C16—C17—B12	123.74 (12)	C17—B12—B17	59.04 (9)
C18—C17—B12	112.59 (11)	B16—B12—B17	108.57 (13)
B11—C17—B12	62.62 (9)	B18—B12—B17	60.02 (10)
B13—C17—B12	113.86 (12)	C17—B12—B11	58.45 (9)
C16—C17—B17	119.07 (12)	B16—B12—B11	59.89 (10)
C18—C17—B17	112.66 (12)	B18—B12—B11	108.37 (12)
B11—C17—B17	114.37 (12)	B17—B12—B11	108.28 (12)
B13—C17—B17	62.59 (10)	C17—B12—H36	117.6 (10)
B12—C17—B17	62.25 (10)	B16—B12—H36	127.2 (10)
C17—C18—B14	108.73 (11)	B18—B12—H36	128.4 (11)
C17—C18—B15	108.38 (12)	B17—B12—H36	119.6 (11)
B14—C18—B15	61.56 (10)	B11—B12—H36	118.0 (11)
C17—C18—B13	60.77 (9)	C17—B13—C18	57.44 (9)
B14—C18—B13	111.65 (12)	C17—B13—B15	104.59 (12)
B15—C18—B13	61.00 (10)	C18—B13—B15	59.49 (10)
C17—C18—B11	60.41 (9)	C17—B13—B19	105.22 (12)
B14—C18—B11	61.51 (10)	C18—B13—B19	106.88 (12)
B15—C18—B11	111.52 (11)	B15—B13—B19	60.30 (10)
B13—C18—B11	112.07 (11)	C17—B13—B17	58.94 (9)
C17—C18—SN	112.43 (9)	C18—B13—B17	106.19 (11)
B14—C18—SN	133.77 (10)	B15—B13—B17	108.01 (12)
B15—C18—SN	120.85 (9)	B19—B13—B17	60.05 (10)
B13—C18—SN	106.25 (9)	C17—B13—H37	118.5 (11)
B11—C18—SN	125.30 (10)	C18—B13—H37	119.8 (11)
N3—C19—C24	109.96 (13)	B15—B13—H37	126.3 (11)
N3—C19—C20	110.05 (13)	B19—B13—H37	127.8 (11)
C24—C19—C20	108.32 (13)	B17—B13—H37	120.8 (11)
N3—C19—H45	109.5	C18—B14—B15	59.55 (9)
C24—C19—H45	109.5	C18—B14—B11	59.75 (9)
C20—C19—H45	109.5	B15—B14—B11	108.49 (12)
C19—C20—C21	111.32 (14)	C18—B14—B16	106.41 (12)
C19—C20—H46	109.4	B15—B14—B16	107.81 (13)
C21—C20—H46	109.4	B11—B14—B16	59.82 (10)
C19—C20—H47	109.4	C18—B14—B20	106.43 (12)
C21—C20—H47	109.4	B15—B14—B20	59.84 (10)
H46—C20—H47	108.0	B11—B14—B20	108.00 (12)
C22—C21—C20	111.13 (15)	B16—B14—B20	59.87 (10)
C22—C21—H49	109.4	C18—B14—H38	117.5 (11)
C20—C21—H49	109.4	B15—B14—H38	121.2 (11)
C22—C21—H48	109.4	B11—B14—H38	118.0 (11)
C20—C21—H48	109.4	B16—B14—H38	125.6 (10)
H49—C21—H48	108.0	B20—B14—H38	127.5 (11)
C23—C22—C21	110.72 (15)	C18—B15—B13	59.51 (10)
C23—C22—H51	109.5	C18—B15—B14	58.89 (10)
C21—C22—H51	109.5	B13—B15—B14	108.25 (12)
C23—C22—H50	109.5	C18—B15—B19	106.70 (12)
C21—C22—H50	109.5	B13—B15—B19	60.04 (10)
H51—C22—H50	108.1	B14—B15—B19	109.16 (12)
C22—C23—C24	111.18 (15)	C18—B15—B20	106.56 (12)
C22—C23—H52	109.4	B13—B15—B20	108.61 (13)
C24—C23—H52	109.4	B14—B15—B20	60.66 (10)
C22—C23—H53	109.4	B19—B15—B20	60.66 (11)
C24—C23—H53	109.4	C18—B15—H39	120.7 (11)
H52—C23—H53	108.0	B13—B15—H39	118.6 (11)
C19—C24—C23	111.23 (14)	B14—B15—H39	122.3 (11)
C19—C24—H55	109.4	B19—B15—H39	122.3 (11)
C23—C24—H55	109.4	B20—B15—H39	124.9 (11)
C19—C24—H54	109.4	B12—B16—B11	60.39 (10)
C23—C24—H54	109.4	B12—B16—B20	107.63 (12)
H55—C24—H54	108.0	B11—B16—B20	108.00 (12)
N4—C25—C26	108.61 (13)	B12—B16—B18	59.72 (10)
N4—C25—C30	111.66 (13)	B11—B16—B18	108.08 (12)
C26—C25—C30	110.56 (13)	B20—B16—B18	59.75 (10)
N4—C25—H56	108.7	B12—B16—B14	107.63 (12)
C26—C25—H56	108.7	B11—B16—B14	59.60 (10)
C30—C25—H56	108.7	B20—B16—B14	60.10 (10)
C25—C26—C27	113.27 (14)	B18—B16—B14	107.61 (12)
C25—C26—H58	108.9	B12—B16—H40	120.1 (11)
C27—C26—H58	108.9	B11—B16—H40	117.9 (11)
C25—C26—H57	108.9	B20—B16—H40	125.1 (11)
C27—C26—H57	108.9	B18—B16—H40	124.5 (11)
H58—C26—H57	107.7	B14—B16—H40	121.4 (11)
C28—C27—C26	111.13 (15)	C17—B17—B18	104.46 (12)
C28—C27—H59	109.4	C17—B17—B19	104.33 (12)
C26—C27—H59	109.4	B18—B17—B19	60.48 (10)
C28—C27—H60	109.4	C17—B17—B12	58.72 (9)
C26—C27—H60	109.4	B18—B17—B12	59.72 (10)
H59—C27—H60	108.0	B19—B17—B12	108.06 (13)
C27—C28—C29	110.30 (15)	C17—B17—B13	58.47 (9)
C27—C28—H62	109.6	B18—B17—B13	107.58 (12)
C29—C28—H62	109.6	B19—B17—B13	59.38 (10)
C27—C28—H61	109.6	B12—B17—B13	107.60 (12)
C29—C28—H61	109.6	C17—B17—H41	118.6 (11)
H62—C28—H61	108.1	B18—B17—H41	128.0 (11)
C28—C29—C30	111.30 (16)	B19—B17—H41	126.8 (11)
C28—C29—H64	109.4	B12—B17—H41	120.2 (11)
C30—C29—H64	109.4	B13—B17—H41	118.9 (10)
C28—C29—H63	109.4	B12—B18—B17	60.26 (10)
C30—C29—H63	109.4	B12—B18—B20	107.87 (12)
H64—C29—H63	108.0	B17—B18—B20	108.12 (12)
C29—C30—C25	112.63 (14)	B12—B18—B16	59.70 (10)
C29—C30—H66	109.1	B17—B18—B16	108.07 (12)
C25—C30—H66	109.1	B20—B18—B16	60.07 (10)
C29—C30—H65	109.1	B12—B18—B19	107.97 (12)
C25—C30—H65	109.1	B17—B18—B19	59.83 (10)
H66—C30—H65	107.8	B20—B18—B19	60.20 (10)
C2—B1—C3	57.96 (9)	B16—B18—B19	108.13 (12)
C2—B1—B8	104.58 (12)	B12—B18—H42	119.7 (11)
C3—B1—B8	106.11 (12)	B17—B18—H42	121.0 (11)
C2—B1—B7	104.15 (12)	B20—B18—H42	123.3 (11)
C3—B1—B7	58.84 (9)	B16—B18—H42	121.0 (11)
B8—B1—B7	59.89 (10)	B19—B18—H42	123.4 (11)
C2—B1—B2	58.71 (9)	B13—B19—B15	59.65 (10)
C3—B1—B2	106.24 (12)	B13—B19—B17	60.57 (10)
B8—B1—B2	59.76 (10)	B15—B19—B17	107.90 (12)
B7—B1—B2	107.37 (12)	B13—B19—B20	107.57 (13)
C2—B1—H2	116.6 (11)	B15—B19—B20	59.75 (10)
C3—B1—H2	117.3 (10)	B17—B19—B20	107.51 (13)
B8—B1—H2	131.2 (10)	B13—B19—B18	107.90 (12)
B7—B1—H2	127.0 (11)	B15—B19—B18	107.29 (13)
B2—B1—H2	122.1 (11)	B17—B19—B18	59.69 (10)
C2—B2—B5	104.64 (12)	B20—B19—B18	59.57 (10)
C2—B2—B8	104.27 (12)	B13—B19—H43	120.9 (11)
B5—B2—B8	60.46 (10)	B15—B19—H43	120.0 (11)
C2—B2—B3	58.81 (9)	B17—B19—H43	123.3 (12)
B5—B2—B3	59.75 (10)	B20—B19—H43	121.5 (11)
B8—B2—B3	107.96 (13)	B18—B19—H43	123.9 (11)
C2—B2—B1	58.34 (9)	B15—B20—B18	107.68 (13)
B5—B2—B1	108.11 (12)	B15—B20—B16	107.67 (12)
B8—B2—B1	59.73 (10)	B18—B20—B16	60.17 (10)
B3—B2—B1	107.77 (12)	B15—B20—B14	59.51 (10)
C2—B2—H3	117.5 (11)	B18—B20—B14	107.89 (13)
B5—B2—H3	128.5 (11)	B16—B20—B14	60.03 (10)
B8—B2—H3	127.9 (10)	B15—B20—B19	59.59 (10)
B3—B2—H3	119.5 (10)	B18—B20—B19	60.22 (10)
B1—B2—H3	118.5 (11)	B16—B20—B19	108.22 (13)
C2—B3—B6	104.79 (12)	B14—B20—B19	107.40 (12)
C2—B3—B5	104.76 (12)	B15—B20—H44	121.6 (11)
B6—B3—B5	61.08 (10)	B18—B20—H44	122.9 (11)
C2—B3—B2	58.75 (9)	B16—B20—H44	121.2 (11)
B6—B3—B2	108.97 (12)	B14—B20—H44	120.6 (11)
B5—B3—B2	59.97 (10)	B19—B20—H44	122.9 (11)
C2—B3—B4	58.14 (9)	C1—N1—C4	120.66 (13)
B6—B3—B4	60.13 (10)	C1—N1—SN	117.98 (10)
B5—B3—B4	108.62 (12)	C4—N1—SN	121.02 (9)
B2—B3—B4	107.85 (12)	C1—N2—C10	130.17 (13)
C2—B3—H4	117.9 (11)	C1—N2—H1	114.2 (15)
B6—B3—H4	126.0 (11)	C10—N2—H1	112.9 (15)
B5—B3—H4	128.8 (11)	C16—N3—C19	121.43 (13)
B2—B3—H4	120.7 (11)	C16—N3—SN	119.37 (10)
B4—B3—H4	117.2 (11)	C19—N3—SN	119.02 (9)
C2—B4—C3	57.74 (9)	C16—N4—C25	129.66 (13)
C2—B4—B9	104.45 (11)	C16—N4—H34	114.1 (14)
C3—B4—B9	58.94 (9)	C25—N4—H34	115.1 (14)
C2—B4—B6	104.83 (12)	C3—SN—C18	100.12 (5)
C3—B4—B6	106.21 (12)	C3—SN—N3	87.83 (5)
B9—B4—B6	60.23 (10)	C18—SN—N3	73.27 (5)
C2—B4—B3	58.90 (9)	C3—SN—N1	72.01 (5)
C3—B4—B3	105.87 (12)	C18—SN—N1	100.52 (5)
B9—B4—B3	107.31 (12)	N3—SN—N1	157.76 (4)
			
N1—C1—C2—C3	9.78 (19)	B4—B9—B10—B5	0.57 (17)
N2—C1—C2—C3	−172.44 (12)	B6—B9—B10—B5	38.14 (12)
N1—C1—C2—B4	80.50 (17)	C3—B9—B10—B6	−100.36 (13)
N2—C1—C2—B4	−101.72 (15)	B7—B9—B10—B6	−138.61 (13)
N1—C1—C2—B1	−59.61 (17)	B4—B9—B10—B6	−37.58 (12)
N2—C1—C2—B1	118.17 (14)	C3—B9—B10—B8	1.21 (16)
N1—C1—C2—B2	−132.02 (14)	B7—B9—B10—B8	−37.04 (12)
N2—C1—C2—B2	45.76 (18)	B4—B9—B10—B8	64.00 (15)
N1—C1—C2—B3	153.77 (14)	B6—B9—B10—B8	101.57 (13)
N2—C1—C2—B3	−28.44 (19)	B3—B6—B10—B7	64.04 (15)
C1—C2—C3—B7	−147.19 (12)	B4—B6—B10—B7	0.39 (17)
B4—C2—C3—B7	104.82 (12)	B9—B6—B10—B7	−36.68 (12)
B1—C2—C3—B7	−40.73 (11)	B5—B6—B10—B7	100.90 (13)
B2—C2—C3—B7	−2.49 (16)	B3—B6—B10—B5	−36.86 (12)
B3—C2—C3—B7	65.81 (15)	B4—B6—B10—B5	−100.51 (13)
C1—C2—C3—B1	−106.45 (14)	B9—B6—B10—B5	−137.58 (13)
B4—C2—C3—B1	145.55 (12)	B3—B6—B10—B9	100.72 (13)
B2—C2—C3—B1	38.25 (12)	B4—B6—B10—B9	37.07 (12)
B3—C2—C3—B1	106.55 (13)	B5—B6—B10—B9	137.58 (13)
C1—C2—C3—B9	147.55 (12)	B3—B6—B10—B8	1.01 (16)
B4—C2—C3—B9	39.56 (11)	B4—B6—B10—B8	−62.64 (16)
B1—C2—C3—B9	−105.99 (12)	B9—B6—B10—B8	−99.71 (13)
B2—C2—C3—B9	−67.75 (14)	B5—B6—B10—B8	37.87 (12)
B3—C2—C3—B9	0.55 (15)	B1—B8—B10—B7	−37.27 (12)
C1—C2—C3—B4	107.99 (14)	B2—B8—B10—B7	−101.16 (13)
B1—C2—C3—B4	−145.55 (12)	B5—B8—B10—B7	−138.18 (12)
B2—C2—C3—B4	−107.30 (13)	B7—B8—B10—B5	138.18 (12)
B3—C2—C3—B4	−39.01 (11)	B1—B8—B10—B5	100.91 (13)
C1—C2—C3—SN	5.08 (15)	B2—B8—B10—B5	37.02 (12)
B4—C2—C3—SN	−102.92 (10)	B7—B8—B10—B9	36.95 (12)
B1—C2—C3—SN	111.53 (11)	B1—B8—B10—B9	−0.33 (16)
B2—C2—C3—SN	149.78 (10)	B2—B8—B10—B9	−64.21 (15)
B3—C2—C3—SN	−141.92 (10)	B5—B8—B10—B9	−101.23 (13)
N1—C4—C5—C6	−173.15 (13)	B7—B8—B10—B6	100.13 (13)
C9—C4—C5—C6	−52.41 (18)	B1—B8—B10—B6	62.86 (16)
C4—C5—C6—C7	58.06 (18)	B2—B8—B10—B6	−1.03 (16)
C5—C6—C7—C8	−60.56 (18)	B5—B8—B10—B6	−38.05 (12)
C6—C7—C8—C9	58.22 (19)	C16—C17—B11—C18	105.20 (14)
N1—C4—C9—C8	168.77 (13)	B13—C17—B11—C18	−34.30 (12)
C5—C4—C9—C8	49.31 (18)	B12—C17—B11—C18	−139.71 (12)
C7—C8—C9—C4	−52.70 (19)	B17—C17—B11—C18	−103.83 (13)
N2—C10—C11—C12	−68.30 (17)	C16—C17—B11—B14	142.37 (13)
C15—C10—C11—C12	51.19 (17)	C18—C17—B11—B14	37.17 (11)
C10—C11—C12—C13	−53.98 (18)	B13—C17—B11—B14	2.87 (16)
C11—C12—C13—C14	55.56 (18)	B12—C17—B11—B14	−102.53 (12)
C12—C13—C14—C15	−54.77 (18)	B17—C17—B11—B14	−66.66 (15)
C13—C14—C15—C10	52.85 (18)	C16—C17—B11—B16	−154.82 (13)
N2—C10—C15—C14	71.29 (16)	C18—C17—B11—B16	99.97 (12)
C11—C10—C15—C14	−50.83 (17)	B13—C17—B11—B16	65.67 (15)
N3—C16—C17—C18	−17.75 (19)	B12—C17—B11—B16	−39.73 (11)
N4—C16—C17—C18	166.19 (12)	B17—C17—B11—B16	−3.86 (16)
N3—C16—C17—B11	−88.99 (17)	C16—C17—B11—B12	−115.09 (14)
N4—C16—C17—B11	94.96 (16)	C18—C17—B11—B12	139.71 (12)
N3—C16—C17—B13	50.91 (18)	B13—C17—B11—B12	105.40 (13)
N4—C16—C17—B13	−125.15 (14)	B17—C17—B11—B12	35.87 (12)
N3—C16—C17—B12	−164.23 (13)	B14—C18—B11—C17	136.75 (12)
N4—C16—C17—B12	19.7 (2)	B15—C18—B11—C17	99.55 (13)
N3—C16—C17—B17	121.40 (15)	B13—C18—B11—C17	33.27 (11)
N4—C16—C17—B17	−54.66 (18)	SN—C18—B11—C17	−97.79 (12)
C16—C17—C18—B14	−151.20 (13)	C17—C18—B11—B14	−136.75 (12)
B11—C17—C18—B14	−39.49 (12)	B15—C18—B11—B14	−37.20 (12)
B13—C17—C18—B14	104.88 (13)	B13—C18—B11—B14	−103.47 (13)
B12—C17—C18—B14	−1.03 (16)	SN—C18—B11—B14	125.46 (13)
B17—C17—C18—B14	67.08 (15)	C17—C18—B11—B16	−97.55 (12)
C16—C17—C18—B15	143.46 (12)	B14—C18—B11—B16	39.20 (11)
B11—C17—C18—B15	−104.83 (12)	B15—C18—B11—B16	2.00 (16)
B13—C17—C18—B15	39.54 (11)	B13—C18—B11—B16	−64.28 (15)
B12—C17—C18—B15	−66.37 (15)	SN—C18—B11—B16	164.66 (10)
B17—C17—C18—B15	1.74 (15)	C17—C18—B11—B12	−35.15 (11)
C16—C17—C18—B13	103.92 (14)	B14—C18—B11—B12	101.59 (13)
B11—C17—C18—B13	−144.37 (12)	B15—C18—B11—B12	64.39 (15)
B12—C17—C18—B13	−105.91 (13)	B13—C18—B11—B12	−1.88 (16)
B17—C17—C18—B13	−37.80 (12)	SN—C18—B11—B12	−132.95 (11)
C16—C17—C18—B11	−111.71 (14)	C16—C17—B12—B16	148.88 (14)
B13—C17—C18—B11	144.37 (12)	C18—C17—B12—B16	1.50 (16)
B12—C17—C18—B11	38.46 (12)	B11—C17—B12—B16	39.76 (12)
B17—C17—C18—B11	106.57 (13)	B13—C17—B12—B16	−66.43 (15)
C16—C17—C18—SN	7.26 (15)	B17—C17—B12—B16	−103.14 (13)
B11—C17—C18—SN	118.98 (11)	C16—C17—B12—B18	−148.24 (14)
B13—C17—C18—SN	−96.66 (10)	C18—C17—B12—B18	64.38 (15)
B12—C17—C18—SN	157.43 (10)	B11—C17—B12—B18	102.65 (13)
B17—C17—C18—SN	−134.46 (10)	B13—C17—B12—B18	−3.54 (16)
N3—C19—C20—C21	178.08 (14)	B17—C17—B12—B18	−40.25 (11)
C24—C19—C20—C21	57.83 (18)	C16—C17—B12—B17	−107.99 (15)
C19—C20—C21—C22	−56.6 (2)	C18—C17—B12—B17	104.63 (13)
C20—C21—C22—C23	54.5 (2)	B11—C17—B12—B17	142.90 (13)
C21—C22—C23—C24	−55.4 (2)	B13—C17—B12—B17	36.71 (12)
N3—C19—C24—C23	−179.02 (13)	C16—C17—B12—B11	109.11 (15)
C20—C19—C24—C23	−58.72 (18)	C18—C17—B12—B11	−38.27 (12)
C22—C23—C24—C19	58.64 (19)	B13—C17—B12—B11	−106.19 (13)
N4—C25—C26—C27	−71.10 (17)	B17—C17—B12—B11	−142.90 (13)
C30—C25—C26—C27	51.73 (18)	C18—B11—B12—C17	34.51 (10)
C25—C26—C27—C28	−54.8 (2)	B14—B11—B12—C17	96.13 (12)
C26—C27—C28—C29	56.3 (2)	B16—B11—B12—C17	134.32 (13)
C27—C28—C29—C30	−56.9 (2)	C17—B11—B12—B16	−134.32 (13)
C28—C29—C30—C25	55.4 (2)	C18—B11—B12—B16	−99.82 (13)
N4—C25—C30—C29	69.15 (18)	B14—B11—B12—B16	−38.20 (11)
C26—C25—C30—C29	−51.88 (19)	C17—B11—B12—B18	−96.59 (13)
C1—C2—B1—C3	107.58 (14)	C18—B11—B12—B18	−62.08 (14)
B4—C2—B1—C3	−33.43 (12)	B14—B11—B12—B18	−0.46 (15)
B2—C2—B1—C3	−140.02 (12)	B16—B11—B12—B18	37.73 (12)
B3—C2—B1—C3	−103.55 (13)	C17—B11—B12—B17	−33.01 (11)
C1—C2—B1—B8	−152.32 (12)	C18—B11—B12—B17	1.50 (15)
C3—C2—B1—B8	100.10 (12)	B14—B11—B12—B17	63.12 (15)
B4—C2—B1—B8	66.67 (15)	B16—B11—B12—B17	101.32 (13)
B2—C2—B1—B8	−39.92 (11)	C16—C17—B13—C18	−107.71 (13)
B3—C2—B1—B8	−3.45 (16)	B11—C17—B13—C18	34.44 (11)
C1—C2—B1—B7	145.65 (13)	B12—C17—B13—C18	103.84 (13)
C3—C2—B1—B7	38.07 (11)	B17—C17—B13—C18	140.42 (12)
B4—C2—B1—B7	4.64 (16)	C16—C17—B13—B15	−145.65 (13)
B2—C2—B1—B7	−101.95 (13)	C18—C17—B13—B15	−37.94 (11)
B3—C2—B1—B7	−65.48 (15)	B11—C17—B13—B15	−3.50 (16)
C1—C2—B1—B2	−112.40 (14)	B12—C17—B13—B15	65.90 (15)
C3—C2—B1—B2	140.02 (12)	B17—C17—B13—B15	102.48 (13)
B4—C2—B1—B2	106.59 (13)	C16—C17—B13—B19	151.72 (13)
B3—C2—B1—B2	36.47 (12)	C18—C17—B13—B19	−100.57 (13)
B7—C3—B1—C2	135.68 (12)	B11—C17—B13—B19	−66.13 (15)
B9—C3—B1—C2	98.90 (13)	B12—C17—B13—B19	3.27 (16)
B4—C3—B1—C2	31.95 (11)	B17—C17—B13—B19	39.85 (12)
SN—C3—B1—C2	−102.23 (11)	C16—C17—B13—B17	111.87 (14)
C2—C3—B1—B8	−97.38 (13)	C18—C17—B13—B17	−140.42 (12)
B7—C3—B1—B8	38.30 (11)	B11—C17—B13—B17	−105.98 (13)
B9—C3—B1—B8	1.52 (16)	B12—C17—B13—B17	−36.58 (12)
B4—C3—B1—B8	−65.43 (15)	B14—C18—B13—C17	−100.00 (12)
SN—C3—B1—B8	160.39 (10)	B15—C18—B13—C17	−136.32 (12)
C2—C3—B1—B7	−135.68 (12)	B11—C18—B13—C17	−33.14 (11)
B9—C3—B1—B7	−36.78 (12)	SN—C18—B13—C17	107.00 (9)
B4—C3—B1—B7	−103.73 (13)	C17—C18—B13—B15	136.32 (12)
SN—C3—B1—B7	122.09 (12)	B14—C18—B13—B15	36.32 (12)
C2—C3—B1—B2	−34.88 (11)	B11—C18—B13—B15	103.18 (13)
B7—C3—B1—B2	100.80 (13)	SN—C18—B13—B15	−116.69 (10)
B9—C3—B1—B2	64.02 (15)	C17—C18—B13—B19	97.56 (12)
B4—C3—B1—B2	−2.94 (15)	B14—C18—B13—B19	−2.44 (16)
SN—C3—B1—B2	−137.11 (10)	B15—C18—B13—B19	−38.75 (12)
C1—C2—B2—B5	−151.87 (13)	B11—C18—B13—B19	64.42 (15)
C3—C2—B2—B5	64.87 (15)	SN—C18—B13—B19	−155.44 (10)
B4—C2—B2—B5	−3.77 (16)	C17—C18—B13—B17	34.64 (11)
B1—C2—B2—B5	102.49 (13)	B14—C18—B13—B17	−65.37 (15)
B3—C2—B2—B5	−39.93 (11)	B15—C18—B13—B17	−101.68 (13)
C1—C2—B2—B8	145.47 (13)	B11—C18—B13—B17	1.50 (16)
C3—C2—B2—B8	2.22 (16)	SN—C18—B13—B17	141.63 (10)
B4—C2—B2—B8	−66.43 (15)	C17—C18—B14—B15	−101.25 (13)
B1—C2—B2—B8	39.83 (11)	B13—C18—B14—B15	−36.09 (12)
B3—C2—B2—B8	−102.59 (13)	B11—C18—B14—B15	−140.24 (12)
C1—C2—B2—B3	−111.94 (15)	SN—C18—B14—B15	106.78 (14)
C3—C2—B2—B3	104.81 (13)	C17—C18—B14—B11	38.99 (11)
B4—C2—B2—B3	36.16 (12)	B15—C18—B14—B11	140.24 (12)
B1—C2—B2—B3	142.42 (13)	B13—C18—B14—B11	104.15 (13)
C1—C2—B2—B1	105.64 (15)	SN—C18—B14—B11	−112.98 (14)
C3—C2—B2—B1	−37.62 (11)	C17—C18—B14—B16	0.10 (16)
B4—C2—B2—B1	−106.26 (14)	B15—C18—B14—B16	101.35 (13)
B3—C2—B2—B1	−142.42 (13)	B13—C18—B14—B16	65.26 (15)
C3—B1—B2—C2	34.56 (10)	B11—C18—B14—B16	−38.89 (12)
B8—B1—B2—C2	134.05 (13)	SN—C18—B14—B16	−151.87 (11)
B7—B1—B2—C2	96.29 (12)	C17—C18—B14—B20	−62.60 (15)
C2—B1—B2—B5	−96.36 (13)	B15—C18—B14—B20	38.65 (12)
C3—B1—B2—B5	−61.80 (14)	B13—C18—B14—B20	2.55 (16)
B8—B1—B2—B5	37.68 (12)	B11—C18—B14—B20	−101.59 (13)
B7—B1—B2—B5	−0.07 (15)	SN—C18—B14—B20	145.43 (12)
C2—B1—B2—B8	−134.05 (13)	C17—B11—B14—C18	−36.56 (10)
C3—B1—B2—B8	−99.48 (13)	B16—B11—B14—C18	−135.84 (12)
B7—B1—B2—B8	−37.75 (11)	B12—B11—B14—C18	−98.03 (12)
C2—B1—B2—B3	−33.22 (11)	C17—B11—B14—B15	−1.01 (15)
C3—B1—B2—B3	1.35 (15)	C18—B11—B14—B15	35.55 (11)
B8—B1—B2—B3	100.83 (13)	B16—B11—B14—B15	−100.29 (13)
B7—B1—B2—B3	63.08 (15)	B12—B11—B14—B15	−62.48 (15)
C1—C2—B3—B6	−146.16 (13)	C17—B11—B14—B16	99.28 (12)
C3—C2—B3—B6	−0.90 (15)	C18—B11—B14—B16	135.84 (12)
B4—C2—B3—B6	−39.60 (11)	B12—B11—B14—B16	37.81 (11)
B1—C2—B3—B6	66.81 (15)	C17—B11—B14—B20	62.34 (14)
B2—C2—B3—B6	103.47 (13)	C18—B11—B14—B20	98.91 (13)
C1—C2—B3—B5	150.44 (13)	B16—B11—B14—B20	−36.93 (12)
C3—C2—B3—B5	−64.31 (15)	B12—B11—B14—B20	0.88 (15)
B4—C2—B3—B5	−103.00 (13)	C17—C18—B15—B13	−39.43 (11)
B1—C2—B3—B5	3.41 (16)	B14—C18—B15—B13	−141.25 (12)
B2—C2—B3—B5	40.07 (11)	B11—C18—B15—B13	−104.07 (13)
C1—C2—B3—B2	110.37 (15)	SN—C18—B15—B13	92.39 (11)
C3—C2—B3—B2	−104.37 (13)	C17—C18—B15—B14	101.82 (12)
B4—C2—B3—B2	−143.07 (12)	B13—C18—B15—B14	141.25 (12)
B1—C2—B3—B2	−36.66 (12)	B11—C18—B15—B14	37.18 (12)
C1—C2—B3—B4	−106.56 (15)	SN—C18—B15—B14	−126.36 (12)
C3—C2—B3—B4	38.70 (11)	C17—C18—B15—B19	−0.84 (15)
B1—C2—B3—B4	106.41 (13)	B14—C18—B15—B19	−102.66 (13)
B2—C2—B3—B4	143.07 (12)	B13—C18—B15—B19	38.59 (11)
B5—B2—B3—C2	134.04 (13)	B11—C18—B15—B19	−65.48 (15)
B8—B2—B3—C2	96.12 (13)	SN—C18—B15—B19	130.98 (11)
B1—B2—B3—C2	33.03 (11)	C17—C18—B15—B20	62.76 (15)
C2—B2—B3—B6	−96.15 (13)	B14—C18—B15—B20	−39.05 (12)
B5—B2—B3—B6	37.88 (12)	B13—C18—B15—B20	102.20 (13)
B8—B2—B3—B6	−0.03 (16)	B11—C18—B15—B20	−1.88 (16)
B1—B2—B3—B6	−63.12 (15)	SN—C18—B15—B20	−165.42 (10)
C2—B2—B3—B5	−134.04 (13)	C17—B13—B15—C18	36.98 (10)
B8—B2—B3—B5	−37.91 (12)	B19—B13—B15—C18	136.41 (12)
B1—B2—B3—B5	−101.00 (13)	B17—B13—B15—C18	98.56 (12)
C2—B2—B3—B4	−32.42 (11)	C17—B13—B15—B14	2.62 (16)
B5—B2—B3—B4	101.62 (13)	C18—B13—B15—B14	−34.36 (11)
B8—B2—B3—B4	63.71 (15)	B19—B13—B15—B14	102.05 (13)
B1—B2—B3—B4	0.61 (16)	B17—B13—B15—B14	64.20 (15)
C1—C2—B4—C3	−107.44 (14)	C17—B13—B15—B19	−99.43 (13)
B1—C2—B4—C3	33.14 (11)	C18—B13—B15—B19	−136.41 (12)
B2—C2—B4—C3	103.35 (13)	B17—B13—B15—B19	−37.85 (12)
B3—C2—B4—C3	139.32 (12)	C17—B13—B15—B20	−61.69 (15)
C1—C2—B4—B9	−145.02 (13)	C18—B13—B15—B20	−98.67 (13)
C3—C2—B4—B9	−37.58 (11)	B19—B13—B15—B20	37.74 (12)
B1—C2—B4—B9	−4.44 (16)	B17—B13—B15—B20	−0.11 (17)
B2—C2—B4—B9	65.77 (15)	B11—B14—B15—C18	−35.64 (11)
B3—C2—B4—B9	101.74 (13)	B16—B14—B15—C18	−98.93 (13)
C1—C2—B4—B6	152.51 (13)	B20—B14—B15—C18	−136.15 (12)
C3—C2—B4—B6	−100.05 (12)	C18—B14—B15—B13	34.61 (11)
B1—C2—B4—B6	−66.91 (15)	B11—B14—B15—B13	−1.03 (16)
B2—C2—B4—B6	3.30 (16)	B16—B14—B15—B13	−64.33 (15)
B3—C2—B4—B6	39.27 (11)	B20—B14—B15—B13	−101.54 (14)
C1—C2—B4—B3	113.24 (14)	C18—B14—B15—B19	98.37 (13)
C3—C2—B4—B3	−139.32 (12)	B11—B14—B15—B19	62.74 (15)
B1—C2—B4—B3	−106.18 (13)	B16—B14—B15—B19	−0.56 (16)
B2—C2—B4—B3	−35.97 (12)	B20—B14—B15—B19	−37.77 (12)
B7—C3—B4—C2	−99.80 (13)	C18—B14—B15—B20	136.15 (12)
B1—C3—B4—C2	−32.16 (11)	B11—B14—B15—B20	100.51 (13)
B9—C3—B4—C2	−136.42 (12)	B16—B14—B15—B20	37.21 (12)
SN—C3—B4—C2	105.36 (10)	C17—B12—B16—B11	−39.06 (11)
C2—C3—B4—B9	136.42 (12)	B18—B12—B16—B11	−138.18 (12)
B7—C3—B4—B9	36.62 (12)	B17—B12—B16—B11	−100.81 (13)
B1—C3—B4—B9	104.26 (13)	C17—B12—B16—B20	62.03 (15)
SN—C3—B4—B9	−118.22 (11)	B18—B12—B16—B20	−37.10 (11)
C2—C3—B4—B6	97.56 (13)	B17—B12—B16—B20	0.27 (16)
B7—C3—B4—B6	−2.24 (16)	B11—B12—B16—B20	101.09 (13)
B1—C3—B4—B6	65.40 (15)	C17—B12—B16—B18	99.13 (12)
B9—C3—B4—B6	−38.86 (11)	B17—B12—B16—B18	37.37 (11)
SN—C3—B4—B6	−157.08 (10)	B11—B12—B16—B18	138.18 (12)
C2—C3—B4—B3	35.47 (10)	C17—B12—B16—B14	−1.36 (16)
B7—C3—B4—B3	−64.33 (15)	B18—B12—B16—B14	−100.49 (13)
B1—C3—B4—B3	3.31 (15)	B17—B12—B16—B14	−63.12 (15)
B9—C3—B4—B3	−100.95 (13)	B11—B12—B16—B14	37.70 (12)
SN—C3—B4—B3	140.83 (10)	C17—B11—B16—B12	39.35 (11)
B6—B3—B4—C2	134.71 (12)	C18—B11—B16—B12	99.16 (12)
B5—B3—B4—C2	96.15 (13)	B14—B11—B16—B12	137.49 (13)
B2—B3—B4—C2	32.66 (11)	C17—B11—B16—B20	−61.12 (15)
C2—B3—B4—C3	−34.97 (10)	C18—B11—B16—B20	−1.30 (16)
B6—B3—B4—C3	99.74 (12)	B14—B11—B16—B20	37.03 (12)
B5—B3—B4—C3	61.19 (15)	B12—B11—B16—B20	−100.47 (13)
B2—B3—B4—C3	−2.31 (15)	C17—B11—B16—B18	2.07 (15)
C2—B3—B4—B9	−96.73 (12)	C18—B11—B16—B18	61.88 (15)
B6—B3—B4—B9	37.99 (12)	B14—B11—B16—B18	100.22 (13)
B5—B3—B4—B9	−0.57 (16)	B12—B11—B16—B18	−37.28 (11)
B2—B3—B4—B9	−64.07 (15)	C17—B11—B16—B14	−98.15 (12)
C2—B3—B4—B6	−134.71 (12)	C18—B11—B16—B14	−38.33 (11)
B5—B3—B4—B6	−38.56 (12)	B12—B11—B16—B14	−137.49 (13)
B2—B3—B4—B6	−102.05 (13)	C18—B14—B16—B12	0.81 (16)
C2—B3—B5—B2	−39.46 (11)	B15—B14—B16—B12	63.40 (15)
B6—B3—B5—B2	−138.44 (13)	B11—B14—B16—B12	−38.05 (12)
B4—B3—B5—B2	−100.30 (13)	B20—B14—B16—B12	100.60 (14)
C2—B3—B5—B10	61.76 (15)	C18—B14—B16—B11	38.86 (11)
B6—B3—B5—B10	−37.21 (12)	B15—B14—B16—B11	101.45 (13)
B2—B3—B5—B10	101.22 (14)	B20—B14—B16—B11	138.65 (13)
B4—B3—B5—B10	0.92 (16)	C18—B14—B16—B20	−99.79 (13)
C2—B3—B5—B8	−1.88 (16)	B15—B14—B16—B20	−37.20 (12)
B6—B3—B5—B8	−100.85 (14)	B11—B14—B16—B20	−138.65 (13)
B2—B3—B5—B8	37.58 (12)	C18—B14—B16—B18	−62.17 (15)
B4—B3—B5—B8	−62.72 (15)	B15—B14—B16—B18	0.42 (16)
C2—B3—B5—B6	98.98 (13)	B11—B14—B16—B18	−101.03 (13)
B2—B3—B5—B6	138.44 (13)	B20—B14—B16—B18	37.61 (12)
B4—B3—B5—B6	38.14 (12)	C16—C17—B17—B18	155.18 (13)
C2—B2—B5—B3	39.46 (11)	C18—C17—B17—B18	−64.51 (14)
B8—B2—B5—B3	137.79 (13)	B11—C17—B17—B18	3.99 (16)
B1—B2—B5—B3	100.44 (13)	B13—C17—B17—B18	−101.99 (13)
C2—B2—B5—B10	−60.92 (15)	B12—C17—B17—B18	40.00 (11)
B8—B2—B5—B10	37.41 (12)	C16—C17—B17—B19	−142.16 (13)
B3—B2—B5—B10	−100.39 (13)	C18—C17—B17—B19	−1.86 (15)
B1—B2—B5—B10	0.05 (16)	B11—C17—B17—B19	66.64 (15)
C2—B2—B5—B8	−98.33 (13)	B13—C17—B17—B19	−39.33 (11)
B3—B2—B5—B8	−137.79 (13)	B12—C17—B17—B19	102.66 (13)
B1—B2—B5—B8	−37.36 (11)	C16—C17—B17—B12	115.18 (14)
C2—B2—B5—B6	2.59 (16)	C18—C17—B17—B12	−104.51 (13)
B8—B2—B5—B6	100.92 (13)	B11—C17—B17—B12	−36.02 (12)
B3—B2—B5—B6	−36.87 (12)	B13—C17—B17—B12	−141.99 (12)
B1—B2—B5—B6	63.56 (15)	C16—C17—B17—B13	−102.83 (14)
C2—B3—B6—B4	38.63 (11)	C18—C17—B17—B13	37.47 (11)
B5—B3—B6—B4	137.56 (13)	B11—C17—B17—B13	105.97 (13)
B2—B3—B6—B4	100.16 (13)	B12—C17—B17—B13	141.99 (12)
C2—B3—B6—B9	0.88 (15)	B16—B12—B17—C17	96.27 (12)
B5—B3—B6—B9	99.81 (13)	B18—B12—B17—C17	133.88 (12)
B2—B3—B6—B9	62.41 (15)	B11—B12—B17—C17	32.78 (11)
B4—B3—B6—B9	−37.75 (11)	C17—B12—B17—B18	−133.88 (12)
C2—B3—B6—B10	−62.14 (14)	B16—B12—B17—B18	−37.61 (12)
B5—B3—B6—B10	36.80 (12)	B11—B12—B17—B18	−101.11 (13)
B2—B3—B6—B10	−0.60 (16)	C17—B12—B17—B19	−96.11 (13)
B4—B3—B6—B10	−100.76 (13)	B16—B12—B17—B19	0.16 (16)
C2—B3—B6—B5	−98.93 (13)	B18—B12—B17—B19	37.77 (12)
B2—B3—B6—B5	−37.40 (12)	B11—B12—B17—B19	−63.34 (15)
B4—B3—B6—B5	−137.56 (13)	C17—B12—B17—B13	−33.41 (11)
C2—B4—B6—B3	−39.01 (11)	B16—B12—B17—B13	62.85 (15)
C3—B4—B6—B3	−99.14 (13)	B18—B12—B17—B13	100.47 (13)
B9—B4—B6—B3	−137.40 (13)	B11—B12—B17—B13	−0.64 (16)
C2—B4—B6—B9	98.39 (12)	C18—B13—B17—C17	−34.00 (11)
C3—B4—B6—B9	38.25 (11)	B15—B13—B17—C17	−96.51 (13)
B3—B4—B6—B9	137.40 (13)	B19—B13—B17—C17	−134.47 (13)
C2—B4—B6—B10	61.20 (15)	C17—B13—B17—B18	96.49 (13)
C3—B4—B6—B10	1.07 (16)	C18—B13—B17—B18	62.49 (15)
B9—B4—B6—B10	−37.19 (12)	B15—B13—B17—B18	−0.02 (16)
B3—B4—B6—B10	100.21 (13)	B19—B13—B17—B18	−37.99 (12)
C2—B4—B6—B5	−1.40 (16)	C17—B13—B17—B19	134.47 (13)
C3—B4—B6—B5	−61.53 (15)	C18—B13—B17—B19	100.47 (13)
B9—B4—B6—B5	−99.79 (13)	B15—B13—B17—B19	37.96 (12)
B3—B4—B6—B5	37.61 (12)	C17—B13—B17—B12	33.51 (11)
B2—B5—B6—B3	37.25 (12)	C18—B13—B17—B12	−0.49 (16)
B10—B5—B6—B3	138.32 (13)	B15—B13—B17—B12	−63.00 (15)
B8—B5—B6—B3	100.45 (13)	B19—B13—B17—B12	−100.96 (14)
B3—B5—B6—B4	−38.02 (12)	C17—B12—B18—B17	39.77 (11)
B2—B5—B6—B4	−0.77 (16)	B16—B12—B18—B17	138.38 (12)
B10—B5—B6—B4	100.30 (13)	B11—B12—B18—B17	100.95 (13)
B8—B5—B6—B4	62.43 (15)	C17—B12—B18—B20	−61.31 (15)
B3—B5—B6—B9	−100.73 (13)	B16—B12—B18—B20	37.30 (11)
B2—B5—B6—B9	−63.47 (15)	B17—B12—B18—B20	−101.08 (13)
B10—B5—B6—B9	37.59 (12)	B11—B12—B18—B20	−0.13 (16)
B8—B5—B6—B9	−0.27 (16)	C17—B12—B18—B16	−98.61 (12)
B3—B5—B6—B10	−138.32 (13)	B17—B12—B18—B16	−138.38 (12)
B2—B5—B6—B10	−101.07 (13)	B11—B12—B18—B16	−37.43 (11)
B8—B5—B6—B10	−37.87 (12)	C17—B12—B18—B19	2.31 (16)
C2—C3—B7—B9	−100.94 (13)	B16—B12—B18—B19	100.92 (13)
B1—C3—B7—B9	−140.97 (13)	B17—B12—B18—B19	−37.46 (12)
B4—C3—B7—B9	−36.56 (12)	B11—B12—B18—B19	63.49 (15)
SN—C3—B7—B9	112.68 (13)	C17—B17—B18—B12	−39.50 (11)
C2—C3—B7—B8	1.63 (15)	B19—B17—B18—B12	−137.99 (13)
B1—C3—B7—B8	−38.40 (12)	B13—B17—B18—B12	−100.50 (13)
B9—C3—B7—B8	102.57 (13)	C17—B17—B18—B20	61.15 (15)
B4—C3—B7—B8	66.01 (15)	B19—B17—B18—B20	−37.34 (12)
SN—C3—B7—B8	−144.74 (11)	B12—B17—B18—B20	100.65 (14)
C2—C3—B7—B10	−61.89 (15)	B13—B17—B18—B20	0.15 (17)
B1—C3—B7—B10	−101.92 (13)	C17—B17—B18—B16	−2.40 (16)
B9—C3—B7—B10	39.05 (12)	B19—B17—B18—B16	−100.89 (13)
B4—C3—B7—B10	2.49 (16)	B12—B17—B18—B16	37.10 (12)
SN—C3—B7—B10	151.74 (11)	B13—B17—B18—B16	−63.40 (15)
C2—C3—B7—B1	40.03 (11)	C17—B17—B18—B19	98.49 (13)
B9—C3—B7—B1	140.97 (13)	B12—B17—B18—B19	137.99 (13)
B4—C3—B7—B1	104.41 (13)	B13—B17—B18—B19	37.49 (12)
SN—C3—B7—B1	−106.35 (13)	B11—B16—B18—B12	37.58 (11)
C2—B1—B7—C3	−37.65 (10)	B20—B16—B18—B12	138.29 (12)
B8—B1—B7—C3	−136.50 (12)	B14—B16—B18—B12	100.52 (13)
B2—B1—B7—C3	−98.81 (12)	B12—B16—B18—B17	−37.35 (11)
C2—B1—B7—B9	−2.85 (15)	B11—B16—B18—B17	0.23 (16)
C3—B1—B7—B9	34.80 (11)	B20—B16—B18—B17	100.94 (13)
B8—B1—B7—B9	−101.70 (13)	B14—B16—B18—B17	63.17 (15)
B2—B1—B7—B9	−64.01 (15)	B12—B16—B18—B20	−138.29 (12)
C2—B1—B7—B8	98.86 (13)	B11—B16—B18—B20	−100.71 (13)
C3—B1—B7—B8	136.50 (12)	B14—B16—B18—B20	−37.77 (12)
B2—B1—B7—B8	37.69 (11)	B12—B16—B18—B19	−100.64 (13)
C2—B1—B7—B10	61.23 (14)	B11—B16—B18—B19	−63.07 (15)
C3—B1—B7—B10	98.87 (13)	B20—B16—B18—B19	37.64 (12)
B8—B1—B7—B10	−37.63 (12)	B14—B16—B18—B19	−0.12 (16)
B2—B1—B7—B10	0.06 (16)	C17—B13—B19—B15	98.36 (13)
C3—B7—B8—B1	37.94 (11)	C18—B13—B19—B15	38.38 (11)
B9—B7—B8—B1	100.57 (13)	B17—B13—B19—B15	137.67 (13)
B10—B7—B8—B1	138.37 (12)	C17—B13—B19—B17	−39.31 (11)
C3—B7—B8—B2	−0.27 (16)	C18—B13—B19—B17	−99.29 (13)
B9—B7—B8—B2	62.35 (16)	B15—B13—B19—B17	−137.67 (13)
B10—B7—B8—B2	100.15 (13)	C17—B13—B19—B20	61.28 (15)
B1—B7—B8—B2	−38.22 (12)	C18—B13—B19—B20	1.30 (16)
C3—B7—B8—B5	−63.36 (15)	B15—B13—B19—B20	−37.08 (12)
B9—B7—B8—B5	−0.73 (16)	B17—B13—B19—B20	100.59 (13)
B10—B7—B8—B5	37.07 (11)	C17—B13—B19—B18	−1.60 (16)
B1—B7—B8—B5	−101.30 (13)	C18—B13—B19—B18	−61.58 (15)
C3—B7—B8—B10	−100.43 (13)	B15—B13—B19—B18	−99.95 (14)
B9—B7—B8—B10	−37.80 (12)	B17—B13—B19—B18	37.71 (12)
B1—B7—B8—B10	−138.37 (12)	C18—B15—B19—B13	−38.34 (11)
C2—B1—B8—B7	−98.12 (13)	B14—B15—B19—B13	−100.51 (13)
C3—B1—B8—B7	−37.81 (11)	B20—B15—B19—B13	−138.29 (13)
B2—B1—B8—B7	−137.51 (13)	C18—B15—B19—B17	−0.29 (16)
C2—B1—B8—B2	39.40 (11)	B13—B15—B19—B17	38.05 (12)
C3—B1—B8—B2	99.70 (13)	B14—B15—B19—B17	−62.46 (16)
B7—B1—B8—B2	137.51 (13)	B20—B15—B19—B17	−100.23 (13)
C2—B1—B8—B5	2.02 (16)	C18—B15—B19—B20	99.94 (13)
C3—B1—B8—B5	62.32 (15)	B13—B15—B19—B20	138.29 (13)
B7—B1—B8—B5	100.13 (13)	B14—B15—B19—B20	37.77 (12)
B2—B1—B8—B5	−37.38 (12)	C18—B15—B19—B18	62.65 (15)
C2—B1—B8—B10	−60.99 (15)	B13—B15—B19—B18	100.99 (13)
C3—B1—B8—B10	−0.69 (16)	B14—B15—B19—B18	0.48 (17)
B7—B1—B8—B10	37.12 (12)	B20—B15—B19—B18	−37.29 (12)
B2—B1—B8—B10	−100.39 (13)	C17—B17—B19—B13	38.89 (11)
C2—B2—B8—B7	−1.11 (16)	B18—B17—B19—B13	137.60 (13)
B5—B2—B8—B7	−100.07 (13)	B12—B17—B19—B13	100.17 (13)
B3—B2—B8—B7	−62.47 (15)	C17—B17—B19—B15	1.24 (16)
B1—B2—B8—B7	38.04 (12)	B18—B17—B19—B15	99.96 (14)
C2—B2—B8—B1	−39.15 (11)	B12—B17—B19—B15	62.53 (15)
B5—B2—B8—B1	−138.10 (13)	B13—B17—B19—B15	−37.64 (12)
B3—B2—B8—B1	−100.51 (13)	C17—B17—B19—B20	−61.80 (14)
C2—B2—B8—B5	98.95 (12)	B18—B17—B19—B20	36.91 (12)
B3—B2—B8—B5	37.60 (12)	B12—B17—B19—B20	−0.52 (16)
B1—B2—B8—B5	138.10 (13)	B13—B17—B19—B20	−100.69 (13)
C2—B2—B8—B10	62.01 (15)	C17—B17—B19—B18	−98.72 (12)
B5—B2—B8—B10	−36.95 (12)	B12—B17—B19—B18	−37.43 (11)
B3—B2—B8—B10	0.65 (16)	B13—B17—B19—B18	−137.60 (13)
B1—B2—B8—B10	101.16 (13)	B12—B18—B19—B13	−0.45 (17)
B3—B5—B8—B7	63.39 (15)	B17—B18—B19—B13	−38.11 (12)
B2—B5—B8—B7	101.19 (13)	B20—B18—B19—B13	100.27 (14)
B10—B5—B8—B7	−37.16 (11)	B16—B18—B19—B13	62.68 (16)
B6—B5—B8—B7	0.61 (16)	B12—B18—B19—B15	−63.35 (16)
B3—B5—B8—B1	−0.08 (17)	B17—B18—B19—B15	−101.01 (13)
B2—B5—B8—B1	37.71 (12)	B20—B18—B19—B15	37.37 (12)
B10—B5—B8—B1	−100.63 (13)	B16—B18—B19—B15	−0.22 (16)
B6—B5—B8—B1	−62.86 (15)	B12—B18—B19—B17	37.65 (12)
B3—B5—B8—B2	−37.80 (12)	B20—B18—B19—B17	138.37 (13)
B10—B5—B8—B2	−138.35 (12)	B16—B18—B19—B17	100.79 (13)
B6—B5—B8—B2	−100.57 (13)	B12—B18—B19—B20	−100.72 (13)
B3—B5—B8—B10	100.55 (13)	B17—B18—B19—B20	−138.37 (13)
B2—B5—B8—B10	138.35 (12)	B16—B18—B19—B20	−37.59 (12)
B6—B5—B8—B10	37.77 (11)	C18—B15—B20—B18	−62.51 (16)
C2—C3—B9—B7	101.99 (12)	B13—B15—B20—B18	0.20 (17)
B1—C3—B9—B7	37.03 (12)	B14—B15—B20—B18	−100.74 (14)
B4—C3—B9—B7	140.93 (13)	B19—B15—B20—B18	37.67 (12)
SN—C3—B9—B7	−120.57 (12)	C18—B15—B20—B16	0.98 (16)
C2—C3—B9—B4	−38.94 (11)	B13—B15—B20—B16	63.70 (16)
B7—C3—B9—B4	−140.93 (13)	B14—B15—B20—B16	−37.25 (12)
B1—C3—B9—B4	−103.90 (13)	B19—B15—B20—B16	101.17 (14)
SN—C3—B9—B4	98.50 (12)	C18—B15—B20—B14	38.23 (11)
C2—C3—B9—B10	63.23 (14)	B13—B15—B20—B14	100.94 (14)
B7—C3—B9—B10	−38.76 (12)	B19—B15—B20—B14	138.41 (13)
B1—C3—B9—B10	−1.73 (16)	C18—B15—B20—B19	−100.18 (13)
B4—C3—B9—B10	102.17 (13)	B13—B15—B20—B19	−37.47 (12)
SN—C3—B9—B10	−159.33 (10)	B14—B15—B20—B19	−138.41 (13)
C2—C3—B9—B6	0.04 (15)	B12—B18—B20—B15	63.50 (16)
B7—C3—B9—B6	−101.95 (13)	B17—B18—B20—B15	−0.22 (17)
B1—C3—B9—B6	−64.92 (15)	B16—B18—B20—B15	100.63 (14)
B4—C3—B9—B6	38.98 (11)	B19—B18—B20—B15	−37.39 (12)
SN—C3—B9—B6	137.48 (11)	B12—B18—B20—B16	−37.13 (11)
B8—B7—B9—C3	−98.25 (13)	B17—B18—B20—B16	−100.85 (13)
B10—B7—B9—C3	−136.11 (13)	B19—B18—B20—B16	−138.02 (13)
B1—B7—B9—C3	−34.62 (11)	B12—B18—B20—B14	0.67 (16)
C3—B7—B9—B4	34.97 (11)	B17—B18—B20—B14	−63.04 (16)
B8—B7—B9—B4	−63.28 (15)	B16—B18—B20—B14	37.81 (12)
B10—B7—B9—B4	−101.14 (13)	B19—B18—B20—B14	−100.22 (13)
B1—B7—B9—B4	0.35 (16)	B12—B18—B20—B19	100.89 (13)
C3—B7—B9—B10	136.11 (13)	B17—B18—B20—B19	37.18 (12)
B8—B7—B9—B10	37.86 (12)	B16—B18—B20—B19	138.02 (13)
B1—B7—B9—B10	101.49 (13)	B12—B16—B20—B15	−63.57 (15)
C3—B7—B9—B6	98.82 (13)	B11—B16—B20—B15	0.20 (17)
B8—B7—B9—B6	0.56 (16)	B18—B16—B20—B15	−100.65 (14)
B10—B7—B9—B6	−37.30 (12)	B14—B16—B20—B15	37.02 (12)
B1—B7—B9—B6	64.19 (15)	B12—B16—B20—B18	37.08 (11)
C2—B4—B9—C3	37.02 (10)	B11—B16—B20—B18	100.86 (13)
B6—B4—B9—C3	136.05 (12)	B14—B16—B20—B18	137.67 (13)
B3—B4—B9—C3	98.43 (12)	B12—B16—B20—B14	−100.59 (13)
C2—B4—B9—B7	2.31 (16)	B11—B16—B20—B14	−36.81 (12)
C3—B4—B9—B7	−34.71 (11)	B18—B16—B20—B14	−137.67 (13)
B6—B4—B9—B7	101.35 (13)	B12—B16—B20—B19	−0.59 (16)
B3—B4—B9—B7	63.72 (15)	B11—B16—B20—B19	63.18 (15)
C2—B4—B9—B10	−61.41 (15)	B18—B16—B20—B19	−37.67 (12)
C3—B4—B9—B10	−98.43 (13)	B14—B16—B20—B19	99.99 (13)
B6—B4—B9—B10	37.62 (12)	C18—B14—B20—B15	−38.51 (11)
B3—B4—B9—B10	0.00 (16)	B11—B14—B20—B15	−101.36 (13)
C2—B4—B9—B6	−99.04 (13)	B16—B14—B20—B15	−138.26 (13)
C3—B4—B9—B6	−136.05 (12)	C18—B14—B20—B18	61.88 (15)
B3—B4—B9—B6	−37.62 (11)	B15—B14—B20—B18	100.39 (14)
B3—B6—B9—C3	−0.58 (16)	B11—B14—B20—B18	−0.96 (16)
B4—B6—B9—C3	−38.73 (11)	B16—B14—B20—B18	−37.87 (12)
B10—B6—B9—C3	99.46 (13)	C18—B14—B20—B16	99.75 (13)
B5—B6—B9—C3	62.07 (14)	B15—B14—B20—B16	138.26 (13)
B3—B6—B9—B7	−62.83 (15)	B11—B14—B20—B16	36.91 (11)
B4—B6—B9—B7	−100.97 (13)	C18—B14—B20—B19	−1.64 (16)
B10—B6—B9—B7	37.21 (12)	B15—B14—B20—B19	36.86 (12)
B5—B6—B9—B7	−0.17 (16)	B11—B14—B20—B19	−64.49 (15)
B3—B6—B9—B4	38.15 (11)	B16—B14—B20—B19	−101.40 (13)
B10—B6—B9—B4	138.19 (13)	B13—B19—B20—B15	37.04 (12)
B5—B6—B9—B4	100.80 (13)	B17—B19—B20—B15	100.90 (13)
B3—B6—B9—B10	−100.04 (13)	B18—B19—B20—B15	137.87 (13)
B4—B6—B9—B10	−138.19 (13)	B13—B19—B20—B18	−100.83 (13)
B5—B6—B9—B10	−37.39 (11)	B15—B19—B20—B18	−137.87 (13)
C3—B7—B10—B5	62.20 (15)	B17—B19—B20—B18	−36.97 (12)
B9—B7—B10—B5	100.78 (13)	B13—B19—B20—B16	−63.17 (15)
B8—B7—B10—B5	−37.40 (12)	B15—B19—B20—B16	−100.21 (13)
B1—B7—B10—B5	−0.04 (16)	B17—B19—B20—B16	0.69 (16)
C3—B7—B10—B9	−38.58 (11)	B18—B19—B20—B16	37.65 (12)
B8—B7—B10—B9	−138.18 (13)	B13—B19—B20—B14	0.21 (16)
B1—B7—B10—B9	−100.81 (13)	B15—B19—B20—B14	−36.83 (12)
C3—B7—B10—B6	−1.72 (16)	B17—B19—B20—B14	64.07 (15)
B9—B7—B10—B6	36.87 (12)	B18—B19—B20—B14	101.04 (13)
B8—B7—B10—B6	−101.32 (13)	N2—C1—N1—C4	−9.7 (2)
B1—B7—B10—B6	−63.95 (15)	C2—C1—N1—C4	167.54 (12)
C3—B7—B10—B8	99.60 (13)	N2—C1—N1—SN	163.67 (13)
B9—B7—B10—B8	138.18 (13)	C2—C1—N1—SN	−19.08 (17)
B1—B7—B10—B8	37.37 (11)	C9—C4—N1—C1	166.86 (13)
B3—B5—B10—B7	−63.69 (15)	C5—C4—N1—C1	−71.01 (17)
B2—B5—B10—B7	−0.01 (16)	C9—C4—N1—SN	−6.32 (16)
B8—B5—B10—B7	37.18 (11)	C5—C4—N1—SN	115.81 (11)
B6—B5—B10—B7	−100.61 (13)	N1—C1—N2—C10	−27.5 (3)
B3—B5—B10—B9	−0.92 (17)	C2—C1—N2—C10	155.19 (15)
B2—B5—B10—B9	62.77 (15)	C11—C10—N2—C1	−57.9 (2)
B8—B5—B10—B9	99.96 (13)	C15—C10—N2—C1	179.89 (15)
B6—B5—B10—B9	−37.83 (12)	N4—C16—N3—C19	19.6 (3)
B3—B5—B10—B6	36.92 (12)	C17—C16—N3—C19	−155.61 (13)
B2—B5—B10—B6	100.60 (13)	N4—C16—N3—SN	−165.20 (13)
B8—B5—B10—B6	137.79 (12)	C17—C16—N3—SN	19.58 (17)
B3—B5—B10—B8	−100.87 (13)	C24—C19—N3—C16	178.86 (14)
B2—B5—B10—B8	−37.19 (11)	C20—C19—N3—C16	59.61 (19)
B6—B5—B10—B8	−137.79 (12)	C24—C19—N3—SN	3.65 (16)
C3—B9—B10—B7	38.25 (11)	C20—C19—N3—SN	−115.60 (12)
B4—B9—B10—B7	101.04 (13)	N3—C16—N4—C25	20.5 (3)
B6—B9—B10—B7	138.61 (13)	C17—C16—N4—C25	−164.17 (14)
C3—B9—B10—B5	−62.22 (15)	C26—C25—N4—C16	−177.34 (15)
B7—B9—B10—B5	−100.47 (14)	C30—C25—N4—C16	60.5 (2)

### Bis(N,N'-diisopropylamidinatocarboranate)chloridotin(IV) (compound_2) Crystal data

**Table:**

[SnCl(C_9_H_24_B_10_N_2_)(C_9_H_25_B_10_N_2_)]	*Z* = 2
*M**_r_* = 691.95	*F*(000) = 704
Triclinic, *P*1	*D*_x_ = 1.291 Mg m^−^^3^
*a* = 11.3063 (4) Å	Mo *K*α radiation, λ = 0.71073 Å
*b* = 13.4631 (4) Å	Cell parameters from 20939 reflections
*c* = 13.7953 (4) Å	θ = 2.0–29.2°
α = 97.078 (2)°	µ = 0.81 mm^−^^1^
β = 106.572 (2)°	*T* = 153 K
γ = 113.377 (2)°	Plate, colorless
*V* = 1779.52 (10) Å^3^	0.34 × 0.23 × 0.04 mm

### Bis(N,N'-diisopropylamidinatocarboranate)chloridotin(IV) (compound_2) Data collection

**Table:**

Stoe IPDS 2T diffractometer	6943 independent reflections
Radiation source: fine-focus sealed tube	5878 reflections with *I* > 2σ(*I*)
Detector resolution: 6.67 pixels mm^-1^	*R*_int_ = 0.055
area detector scans	θ_max_ = 26.0°, θ_min_ = 2.0°
Absorption correction: numerical X-Area and X-Red (Stoe & Cie, 2002)	*h* = −13→13
*T*_min_ = 0.832, *T*_max_ = 0.964	*k* = −16→16
15500 measured reflections	*l* = −17→15

### Bis(N,N'-diisopropylamidinatocarboranate)chloridotin(IV) (compound_2) Refinement

**Table:**

Refinement on *F*^2^	Secondary atom site location: difference Fourier map
Least-squares matrix: full	Hydrogen site location: mixed
*R*[*F*^2^ > 2σ(*F*^2^)] = 0.039	H atoms treated by a mixture of independent and constrained refinement
*wR*(*F*^2^) = 0.092	*w* = 1/[σ^2^(*F*_o_^2^) + (0.0535*P*)^2^] where *P* = (*F*_o_^2^ + 2*F*_c_^2^)/3
*S* = 1.02	(Δ/σ)_max_ = 0.001
6943 reflections	Δρ_max_ = 0.84 e Å^−^^3^
469 parameters	Δρ_min_ = −1.17 e Å^−^^3^
21 restraints	Extinction correction: SHELXL-2016/4 (Sheldrick 2015), Fc^*^=kFc[1+0.001xFc^2^λ^3^/sin(2θ)]^-1/4^
Primary atom site location: heavy-atom method	Extinction coefficient: 0.0050 (6)

### Bis(N,N'-diisopropylamidinatocarboranate)chloridotin(IV) (compound_2) Special details

**Table:**

Geometry. All esds (except the esd in the dihedral angle between two l.s. planes) are estimated using the full covariance matrix. The cell esds are taken into account individually in the estimation of esds in distances, angles and torsion angles; correlations between esds in cell parameters are only used when they are defined by crystal symmetry. An approximate (isotropic) treatment of cell esds is used for estimating esds involving l.s. planes.

### Bis(N,N'-diisopropylamidinatocarboranate)chloridotin(IV) (compound_2) Fractional atomic coordinates and isotropic or equivalent isotropic displacement parameters (Å^2^)

**Table:**

	*x*	*y*	*z*	*U*_iso_*/*U*_eq_	
C1	0.4718 (3)	0.2167 (3)	0.4620 (2)	0.0238 (6)	
C2	0.3778 (3)	0.2181 (3)	0.5359 (2)	0.0242 (6)	
C3	0.6284 (3)	0.2915 (3)	0.5162 (3)	0.0248 (6)	
C4	0.8084 (4)	0.4440 (3)	0.6712 (3)	0.0310 (7)	
H22	0.812609	0.477758	0.741619	0.037*	
C5	0.9204 (4)	0.4057 (3)	0.6924 (3)	0.0355 (8)	
H24	1.005836	0.464911	0.746877	0.053*	
H23	0.937553	0.390043	0.627811	0.053*	
H25	0.890266	0.337335	0.716220	0.053*	
C6	0.8373 (4)	0.5375 (3)	0.6164 (4)	0.0419 (9)	
H28	0.924229	0.603104	0.661454	0.063*	
H26	0.761306	0.558143	0.602364	0.063*	
H27	0.844900	0.511477	0.549905	0.063*	
C7	0.7023 (4)	0.2535 (3)	0.3694 (3)	0.0323 (7)	
H29	0.603301	0.198658	0.328106	0.039*	
C8	0.7936 (5)	0.1945 (4)	0.3786 (4)	0.0486 (10)	
H30	0.785224	0.160707	0.308352	0.073*	
H32	0.764233	0.135636	0.414269	0.073*	
H31	0.890297	0.249131	0.419247	0.073*	
C9	0.7485 (5)	0.3492 (4)	0.3194 (3)	0.0459 (10)	
H34	0.748453	0.320463	0.250622	0.069*	
H35	0.842142	0.406309	0.364613	0.069*	
H33	0.684654	0.382679	0.310810	0.069*	
C10	0.5044 (3)	0.2298 (3)	0.8852 (3)	0.0256 (7)	
C11	0.6076 (3)	0.2712 (3)	0.8189 (2)	0.0247 (6)	
C12	0.3731 (4)	0.2420 (3)	0.8419 (3)	0.0273 (7)	
C13	0.2568 (4)	0.3295 (3)	0.7313 (3)	0.0291 (7)	
H36	0.274735	0.365353	0.674250	0.035*	
C14	0.2794 (4)	0.4235 (3)	0.8202 (3)	0.0392 (9)	
H39	0.219748	0.458103	0.793065	0.059*	
H37	0.376574	0.480443	0.847693	0.059*	
H38	0.256447	0.392041	0.876511	0.059*	
C15	0.1072 (4)	0.2377 (3)	0.6831 (3)	0.0339 (8)	
H41	0.050831	0.263178	0.634482	0.051*	
H40	0.070925	0.221167	0.738863	0.051*	
H42	0.103814	0.169596	0.644833	0.051*	
C16	0.2594 (4)	0.1551 (3)	0.9691 (3)	0.0370 (8)	
H43	0.348126	0.154768	1.009300	0.044*	
C17	0.2248 (7)	0.2243 (6)	1.0410 (4)	0.0724 (16)	
H45	0.210605	0.189638	1.097852	0.109*	
H46	0.140135	0.227397	1.000966	0.109*	
H44	0.301368	0.300545	1.070826	0.109*	
C18	0.1461 (5)	0.0360 (4)	0.9228 (4)	0.0625 (13)	
H47	0.131227	0.001299	0.979444	0.094*	
H49	0.173087	−0.006882	0.878351	0.094*	
H48	0.060313	0.036040	0.880327	0.094*	
B1	0.3731 (4)	0.2867 (3)	0.4397 (3)	0.0265 (7)	
H2	0.429 (4)	0.3794 (16)	0.467 (3)	0.032*	
B2	0.4020 (4)	0.1018 (3)	0.5079 (3)	0.0260 (7)	
H3	0.474 (3)	0.091 (3)	0.577 (2)	0.031*	
B3	0.2402 (4)	0.0921 (3)	0.5063 (3)	0.0285 (8)	
H4	0.209 (4)	0.064 (3)	0.571 (2)	0.034*	
B4	0.3972 (4)	0.0860 (3)	0.3771 (3)	0.0272 (8)	
H5	0.469 (3)	0.058 (3)	0.357 (3)	0.033*	
B5	0.3790 (4)	0.2005 (3)	0.3340 (3)	0.0280 (8)	
H6	0.436 (4)	0.248 (3)	0.286 (3)	0.034*	
B6	0.2221 (4)	0.2067 (3)	0.4637 (3)	0.0283 (8)	
H7	0.174 (4)	0.252 (3)	0.499 (3)	0.034*	
B7	0.2181 (4)	0.1916 (3)	0.3333 (3)	0.0316 (8)	
H8	0.159 (4)	0.224 (3)	0.278 (3)	0.038*	
B8	0.2472 (4)	0.0058 (3)	0.4028 (3)	0.0307 (8)	
H9	0.212 (4)	−0.0855 (17)	0.398 (3)	0.037*	
B9	0.1356 (4)	0.0704 (3)	0.3748 (3)	0.0315 (8)	
H10	0.021 (2)	0.021 (3)	0.346 (3)	0.038*	
B10	0.2337 (4)	0.0672 (3)	0.2956 (3)	0.0309 (8)	
H11	0.187 (4)	0.016 (3)	0.2130 (18)	0.037*	
B11	0.5200 (4)	0.1300 (3)	0.8068 (3)	0.0275 (8)	
H12	0.435 (3)	0.077 (3)	0.732 (2)	0.033*	
B12	0.5164 (4)	0.1222 (3)	0.9336 (3)	0.0310 (8)	
H13	0.422 (3)	0.056 (3)	0.940 (3)	0.037*	
B13	0.6962 (4)	0.1939 (3)	0.8184 (3)	0.0306 (8)	
H14	0.720 (4)	0.179 (3)	0.747 (2)	0.037*	
B14	0.6413 (5)	0.0989 (3)	0.8933 (3)	0.0334 (9)	
H15	0.632 (4)	0.012 (2)	0.879 (3)	0.040*	
B15	0.5982 (4)	0.2619 (3)	1.0164 (3)	0.0291 (8)	
H16	0.555 (4)	0.282 (3)	1.074 (3)	0.035*	
B16	0.7777 (4)	0.3327 (3)	0.9014 (3)	0.0312 (8)	
H17	0.851 (4)	0.403 (3)	0.880 (3)	0.037*	
B17	0.6538 (4)	0.3567 (3)	0.9409 (3)	0.0285 (8)	
H18	0.640 (4)	0.434 (2)	0.943 (3)	0.034*	
B18	0.8002 (4)	0.2237 (4)	0.9522 (3)	0.0352 (9)	
H19	0.904 (3)	0.226 (3)	0.975 (3)	0.042*	
B19	0.6896 (5)	0.1808 (4)	1.0235 (3)	0.0345 (9)	
H20	0.711 (4)	0.149 (3)	1.094 (2)	0.041*	
B20	0.7732 (4)	0.3252 (4)	1.0270 (3)	0.0331 (8)	
H21	0.856 (3)	0.390 (3)	1.100 (2)	0.040*	
CL	0.56514 (10)	0.51455 (6)	0.72974 (8)	0.0363 (2)	
N1	0.6659 (3)	0.3514 (2)	0.6170 (2)	0.0258 (6)	
N2	0.7189 (3)	0.3026 (2)	0.4756 (2)	0.0305 (6)	
N3	0.3641 (3)	0.2901 (2)	0.7655 (2)	0.0252 (6)	
N4	0.2758 (4)	0.2065 (3)	0.8829 (3)	0.0413 (8)	
H1	0.200 (3)	0.209 (4)	0.847 (3)	0.050*	
SN	0.52251 (2)	0.32768 (2)	0.68914 (2)	0.02208 (9)	

### Bis(N,N'-diisopropylamidinatocarboranate)chloridotin(IV) (compound_2) Atomic displacement parameters (Å^2^)

**Table:**

	*U*^11^	*U*^22^	*U*^33^	*U*^12^	*U*^13^	*U*^23^
C1	0.0300 (16)	0.0240 (14)	0.0200 (15)	0.0146 (13)	0.0090 (13)	0.0065 (12)
C2	0.0287 (16)	0.0261 (15)	0.0222 (15)	0.0158 (13)	0.0100 (13)	0.0078 (12)
C3	0.0279 (16)	0.0237 (15)	0.0259 (16)	0.0135 (13)	0.0104 (13)	0.0093 (12)
C4	0.0270 (17)	0.0264 (16)	0.0330 (18)	0.0103 (14)	0.0077 (14)	0.0002 (13)
C5	0.0307 (18)	0.0399 (19)	0.036 (2)	0.0185 (16)	0.0101 (16)	0.0064 (15)
C6	0.041 (2)	0.0261 (17)	0.062 (3)	0.0133 (16)	0.026 (2)	0.0138 (17)
C7	0.0315 (18)	0.0377 (18)	0.0273 (17)	0.0144 (15)	0.0135 (14)	0.0060 (14)
C8	0.059 (3)	0.061 (3)	0.042 (2)	0.038 (2)	0.027 (2)	0.0101 (19)
C9	0.049 (2)	0.054 (2)	0.032 (2)	0.018 (2)	0.0170 (18)	0.0153 (17)
C10	0.0306 (17)	0.0251 (15)	0.0218 (15)	0.0150 (13)	0.0071 (13)	0.0066 (12)
C11	0.0290 (16)	0.0256 (15)	0.0203 (15)	0.0151 (13)	0.0073 (13)	0.0036 (12)
C12	0.0316 (17)	0.0265 (15)	0.0283 (17)	0.0161 (14)	0.0125 (14)	0.0080 (13)
C13	0.0324 (18)	0.0298 (16)	0.0335 (18)	0.0205 (14)	0.0134 (15)	0.0102 (14)
C14	0.047 (2)	0.0360 (19)	0.044 (2)	0.0267 (17)	0.0197 (18)	0.0067 (16)
C15	0.0299 (18)	0.0364 (18)	0.039 (2)	0.0182 (15)	0.0130 (16)	0.0119 (15)
C16	0.037 (2)	0.051 (2)	0.0326 (19)	0.0227 (17)	0.0181 (16)	0.0203 (16)
C17	0.095 (4)	0.116 (5)	0.047 (3)	0.073 (4)	0.041 (3)	0.029 (3)
C18	0.043 (3)	0.064 (3)	0.068 (3)	0.015 (2)	0.010 (2)	0.034 (3)
B1	0.0302 (19)	0.0272 (17)	0.0268 (19)	0.0174 (15)	0.0097 (15)	0.0099 (14)
B2	0.0302 (18)	0.0210 (16)	0.0265 (18)	0.0113 (14)	0.0108 (15)	0.0055 (13)
B3	0.0295 (19)	0.0253 (17)	0.031 (2)	0.0120 (15)	0.0120 (16)	0.0093 (14)
B4	0.0309 (19)	0.0240 (17)	0.0256 (18)	0.0135 (15)	0.0083 (15)	0.0044 (14)
B5	0.034 (2)	0.0276 (17)	0.0227 (18)	0.0157 (15)	0.0083 (15)	0.0065 (14)
B6	0.0275 (18)	0.0299 (18)	0.0281 (19)	0.0154 (15)	0.0078 (15)	0.0073 (15)
B7	0.032 (2)	0.0331 (19)	0.0274 (19)	0.0152 (16)	0.0071 (16)	0.0089 (15)
B8	0.032 (2)	0.0250 (17)	0.033 (2)	0.0123 (15)	0.0127 (17)	0.0035 (15)
B9	0.0281 (19)	0.0283 (18)	0.029 (2)	0.0094 (15)	0.0056 (16)	0.0023 (15)
B10	0.033 (2)	0.0297 (18)	0.0243 (19)	0.0130 (16)	0.0061 (16)	0.0020 (14)
B11	0.034 (2)	0.0257 (17)	0.0257 (18)	0.0166 (15)	0.0111 (16)	0.0074 (14)
B12	0.039 (2)	0.0276 (18)	0.030 (2)	0.0177 (17)	0.0119 (17)	0.0125 (15)
B13	0.032 (2)	0.0329 (19)	0.033 (2)	0.0206 (17)	0.0126 (17)	0.0091 (16)
B14	0.043 (2)	0.0325 (19)	0.031 (2)	0.0238 (18)	0.0110 (18)	0.0104 (16)
B15	0.041 (2)	0.0298 (18)	0.0212 (17)	0.0188 (16)	0.0127 (16)	0.0074 (14)
B16	0.0299 (19)	0.0345 (19)	0.0267 (19)	0.0170 (17)	0.0049 (16)	0.0043 (15)
B17	0.0319 (19)	0.0255 (17)	0.0223 (18)	0.0115 (15)	0.0063 (15)	0.0012 (14)
B18	0.034 (2)	0.047 (2)	0.030 (2)	0.0251 (19)	0.0084 (17)	0.0131 (17)
B19	0.038 (2)	0.040 (2)	0.028 (2)	0.0238 (18)	0.0068 (17)	0.0108 (16)
B20	0.033 (2)	0.039 (2)	0.0230 (19)	0.0160 (17)	0.0056 (16)	0.0059 (15)
CL	0.0470 (5)	0.0213 (4)	0.0479 (5)	0.0175 (4)	0.0242 (4)	0.0096 (3)
N1	0.0268 (14)	0.0237 (13)	0.0247 (14)	0.0102 (11)	0.0089 (11)	0.0047 (10)
N2	0.0318 (15)	0.0360 (15)	0.0253 (14)	0.0156 (13)	0.0126 (12)	0.0076 (11)
N3	0.0295 (14)	0.0257 (13)	0.0276 (14)	0.0173 (11)	0.0125 (12)	0.0099 (11)
N4	0.0421 (18)	0.064 (2)	0.0430 (19)	0.0352 (17)	0.0263 (16)	0.0331 (16)
SN	0.02621 (12)	0.01939 (11)	0.02173 (12)	0.01194 (8)	0.00828 (8)	0.00486 (7)

### Bis(N,N'-diisopropylamidinatocarboranate)chloridotin(IV) (compound_2) Geometric parameters (Å, º)

**Table:**

C1—C3	1.533 (4)	B1—B6	1.771 (6)
C1—C2	1.673 (4)	B1—B7	1.776 (6)
C1—B4	1.712 (4)	B1—B5	1.783 (5)
C1—B5	1.713 (5)	B1—H2	1.106 (18)
C1—B1	1.714 (5)	B2—B8	1.770 (5)
C1—B2	1.724 (5)	B2—B4	1.774 (5)
C2—B3	1.685 (5)	B2—B3	1.775 (5)
C2—B6	1.692 (5)	B2—H3	1.129 (18)
C2—B1	1.708 (5)	B3—B8	1.767 (5)
C2—B2	1.710 (4)	B3—B9	1.774 (6)
C2—SN	2.160 (3)	B3—B6	1.781 (5)
C3—N2	1.269 (5)	B3—H4	1.108 (19)
C3—N1	1.372 (4)	B4—B10	1.765 (6)
C4—N1	1.482 (4)	B4—B8	1.776 (6)
C4—C5	1.517 (5)	B4—B5	1.783 (5)
C4—C6	1.521 (5)	B4—H5	1.108 (18)
C4—H22	1.0000	B5—B10	1.770 (6)
C5—H24	0.9800	B5—B7	1.773 (6)
C5—H23	0.9800	B5—H6	1.129 (19)
C5—H25	0.9800	B6—B7	1.770 (6)
C6—H28	0.9800	B6—B9	1.779 (5)
C6—H26	0.9800	B6—H7	1.118 (18)
C6—H27	0.9800	B7—B10	1.784 (5)
C7—N2	1.458 (4)	B7—B9	1.785 (6)
C7—C9	1.515 (6)	B7—H8	1.118 (19)
C7—C8	1.521 (5)	B8—B10	1.779 (6)
C7—H29	1.0000	B8—B9	1.781 (6)
C8—H30	0.9800	B8—H9	1.118 (18)
C8—H32	0.9800	B9—B10	1.773 (6)
C8—H31	0.9800	B9—H10	1.113 (19)
C9—H34	0.9800	B10—H11	1.106 (19)
C9—H35	0.9800	B11—B14	1.768 (6)
C9—H33	0.9800	B11—B13	1.777 (6)
C10—C12	1.515 (5)	B11—B12	1.778 (6)
C10—C11	1.651 (5)	B11—H12	1.098 (19)
C10—B12	1.706 (5)	B12—B19	1.772 (6)
C10—B15	1.714 (5)	B12—B14	1.777 (6)
C10—B11	1.717 (5)	B12—B15	1.785 (5)
C10—B17	1.735 (5)	B12—H13	1.121 (19)
C11—B13	1.708 (5)	B13—B14	1.775 (6)
C11—B16	1.714 (5)	B13—B16	1.775 (5)
C11—B11	1.719 (5)	B13—B18	1.776 (6)
C11—B17	1.726 (5)	B13—H14	1.108 (19)
C11—SN	2.154 (3)	B14—B18	1.774 (6)
C12—N3	1.304 (4)	B14—B19	1.793 (6)
C12—N4	1.328 (5)	B14—H15	1.116 (19)
C13—N3	1.491 (4)	B15—B20	1.768 (6)
C13—C15	1.526 (5)	B15—B19	1.770 (6)
C13—C14	1.533 (5)	B15—B17	1.781 (6)
C13—H36	1.0000	B15—H16	1.114 (19)
C14—H39	0.9800	B16—B20	1.762 (6)
C14—H37	0.9800	B16—B17	1.767 (6)
C14—H38	0.9800	B16—B18	1.777 (6)
C15—H41	0.9800	B16—H17	1.120 (19)
C15—H40	0.9800	B17—B20	1.755 (6)
C15—H42	0.9800	B17—H18	1.107 (18)
C16—N4	1.467 (5)	B18—B19	1.770 (6)
C16—C18	1.508 (6)	B18—B20	1.785 (6)
C16—C17	1.512 (6)	B18—H19	1.114 (19)
C16—H43	1.0000	B19—B20	1.780 (6)
C17—H45	0.9800	B19—H20	1.106 (19)
C17—H46	0.9800	B20—H21	1.116 (19)
C17—H44	0.9800	CL—SN	2.3264 (8)
C18—H47	0.9800	N1—SN	2.076 (3)
C18—H49	0.9800	N3—SN	2.250 (3)
C18—H48	0.9800	N4—H1	0.876 (19)
			
C3—C1—C2	115.5 (2)	B6—B7—B1	59.9 (2)
C3—C1—B4	124.5 (3)	B5—B7—B1	60.3 (2)
C2—C1—B4	109.4 (3)	B6—B7—B10	107.5 (3)
C3—C1—B5	124.0 (3)	B5—B7—B10	59.7 (2)
C2—C1—B5	109.8 (3)	B1—B7—B10	107.5 (3)
B4—C1—B5	62.7 (2)	B6—B7—B9	60.1 (2)
C3—C1—B1	115.4 (2)	B5—B7—B9	107.8 (3)
C2—C1—B1	60.5 (2)	B1—B7—B9	107.8 (3)
B4—C1—B1	114.1 (3)	B10—B7—B9	59.6 (2)
B5—C1—B1	62.7 (2)	B6—B7—H8	120 (2)
C3—C1—B2	116.2 (3)	B5—B7—H8	122 (2)
C2—C1—B2	60.44 (19)	B1—B7—H8	120 (2)
B4—C1—B2	62.2 (2)	B10—B7—H8	124 (2)
B5—C1—B2	113.8 (3)	B9—B7—H8	123 (2)
B1—C1—B2	113.1 (3)	B3—B8—B2	60.2 (2)
C1—C2—B3	111.7 (2)	B3—B8—B4	108.1 (3)
C1—C2—B6	111.4 (3)	B2—B8—B4	60.0 (2)
B3—C2—B6	63.7 (2)	B3—B8—B10	107.6 (3)
C1—C2—B1	60.9 (2)	B2—B8—B10	107.5 (3)
B3—C2—B1	115.4 (3)	B4—B8—B10	59.5 (2)
B6—C2—B1	62.8 (2)	B3—B8—B9	60.0 (2)
C1—C2—B2	61.27 (19)	B2—B8—B9	108.1 (3)
B3—C2—B2	63.0 (2)	B4—B8—B9	107.7 (3)
B6—C2—B2	115.5 (3)	B10—B8—B9	59.7 (2)
B1—C2—B2	114.1 (2)	B3—B8—H9	119 (2)
C1—C2—SN	106.55 (19)	B2—B8—H9	117 (2)
B3—C2—SN	128.3 (2)	B4—B8—H9	121 (2)
B6—C2—SN	129.7 (2)	B10—B8—H9	127 (2)
B1—C2—SN	113.1 (2)	B9—B8—H9	125 (2)
B2—C2—SN	111.1 (2)	B10—B9—B3	107.5 (3)
N2—C3—N1	120.5 (3)	B10—B9—B6	107.6 (3)
N2—C3—C1	126.3 (3)	B3—B9—B6	60.2 (2)
N1—C3—C1	113.2 (3)	B10—B9—B8	60.1 (2)
N1—C4—C5	114.1 (3)	B3—B9—B8	59.6 (2)
N1—C4—C6	111.9 (3)	B6—B9—B8	107.9 (3)
C5—C4—C6	112.2 (3)	B10—B9—B7	60.2 (2)
N1—C4—H22	106.0	B3—B9—B7	107.7 (3)
C5—C4—H22	106.0	B6—B9—B7	59.5 (2)
C6—C4—H22	106.0	B8—B9—B7	108.2 (3)
C4—C5—H24	109.5	B10—B9—H10	123 (2)
C4—C5—H23	109.5	B3—B9—H10	121 (2)
H24—C5—H23	109.5	B6—B9—H10	121 (2)
C4—C5—H25	109.5	B8—B9—H10	122 (2)
H24—C5—H25	109.5	B7—B9—H10	122 (2)
H23—C5—H25	109.5	B4—B10—B5	60.6 (2)
C4—C6—H28	109.5	B4—B10—B9	108.5 (3)
C4—C6—H26	109.5	B5—B10—B9	108.5 (3)
H28—C6—H26	109.5	B4—B10—B8	60.1 (2)
C4—C6—H27	109.5	B5—B10—B8	108.7 (3)
H28—C6—H27	109.5	B9—B10—B8	60.2 (2)
H26—C6—H27	109.5	B4—B10—B7	108.4 (3)
N2—C7—C9	107.1 (3)	B5—B10—B7	59.9 (2)
N2—C7—C8	107.4 (3)	B9—B10—B7	60.3 (2)
C9—C7—C8	111.4 (3)	B8—B10—B7	108.4 (3)
N2—C7—H29	110.3	B4—B10—H11	120 (2)
C9—C7—H29	110.3	B5—B10—H11	120 (2)
C8—C7—H29	110.3	B9—B10—H11	123 (2)
C7—C8—H30	109.5	B8—B10—H11	122 (2)
C7—C8—H32	109.5	B7—B10—H11	122 (2)
H30—C8—H32	109.5	C10—B11—C11	57.45 (19)
C7—C8—H31	109.5	C10—B11—B14	104.9 (3)
H30—C8—H31	109.5	C11—B11—B14	104.9 (3)
H32—C8—H31	109.5	C10—B11—B13	104.6 (3)
C7—C9—H34	109.5	C11—B11—B13	58.4 (2)
C7—C9—H35	109.5	B14—B11—B13	60.1 (2)
H34—C9—H35	109.5	C10—B11—B12	58.4 (2)
C7—C9—H33	109.5	C11—B11—B12	104.7 (2)
H34—C9—H33	109.5	B14—B11—B12	60.2 (2)
H35—C9—H33	109.5	B13—B11—B12	107.9 (3)
C12—C10—C11	112.9 (3)	C10—B11—H12	120 (2)
C12—C10—B12	125.8 (3)	C11—B11—H12	118 (2)
C11—C10—B12	111.1 (3)	B14—B11—H12	130 (2)
C12—C10—B15	124.1 (3)	B13—B11—H12	122 (2)
C11—C10—B15	111.0 (3)	B12—B11—H12	126 (2)
B12—C10—B15	62.9 (2)	C10—B12—B19	104.8 (3)
C12—C10—B11	116.1 (3)	C10—B12—B14	104.9 (3)
C11—C10—B11	61.3 (2)	B19—B12—B14	60.7 (2)
B12—C10—B11	62.6 (2)	C10—B12—B11	59.0 (2)
B15—C10—B11	114.5 (3)	B19—B12—B11	108.2 (3)
C12—C10—B17	113.9 (3)	B14—B12—B11	59.6 (2)
C11—C10—B17	61.2 (2)	C10—B12—B15	58.7 (2)
B12—C10—B17	114.1 (3)	B19—B12—B15	59.7 (2)
B15—C10—B17	62.2 (2)	B14—B12—B15	108.3 (3)
B11—C10—B17	114.0 (3)	B11—B12—B15	108.2 (3)
C10—C11—B13	110.8 (3)	C10—B12—H13	119 (2)
C10—C11—B16	110.6 (3)	B19—B12—H13	127 (2)
B13—C11—B16	62.5 (2)	B14—B12—H13	126 (2)
C10—C11—B11	61.2 (2)	B11—B12—H13	119 (2)
B13—C11—B11	62.5 (2)	B15—B12—H13	120 (2)
B16—C11—B11	113.9 (3)	C11—B13—B14	105.1 (3)
C10—C11—B17	61.8 (2)	C11—B13—B16	58.9 (2)
B13—C11—B17	113.8 (3)	B14—B13—B16	108.1 (3)
B16—C11—B17	61.8 (2)	C11—B13—B18	105.1 (3)
B11—C11—B17	114.4 (3)	B14—B13—B18	59.9 (2)
C10—C11—SN	112.3 (2)	B16—B13—B18	60.0 (2)
B13—C11—SN	126.0 (2)	C11—B13—B11	59.1 (2)
B16—C11—SN	125.7 (2)	B14—B13—B11	59.7 (2)
B11—C11—SN	115.2 (2)	B16—B13—B11	108.2 (3)
B17—C11—SN	114.3 (2)	B18—B13—B11	107.7 (3)
N3—C12—N4	123.3 (3)	C11—B13—H14	116 (2)
N3—C12—C10	116.4 (3)	B14—B13—H14	129 (2)
N4—C12—C10	120.2 (3)	B16—B13—H14	119 (2)
N3—C13—C15	115.6 (3)	B18—B13—H14	129 (2)
N3—C13—C14	110.2 (3)	B11—B13—H14	118 (2)
C15—C13—C14	112.9 (3)	B11—B14—B18	108.2 (3)
N3—C13—H36	105.8	B11—B14—B13	60.2 (2)
C15—C13—H36	105.8	B18—B14—B13	60.1 (2)
C14—C13—H36	105.8	B11—B14—B12	60.2 (2)
C13—C14—H39	109.5	B18—B14—B12	107.4 (3)
C13—C14—H37	109.5	B13—B14—B12	108.0 (3)
H39—C14—H37	109.5	B11—B14—B19	107.7 (3)
C13—C14—H38	109.5	B18—B14—B19	59.5 (2)
H39—C14—H38	109.5	B13—B14—B19	107.4 (3)
H37—C14—H38	109.5	B12—B14—B19	59.5 (2)
C13—C15—H41	109.5	B11—B14—H15	121 (2)
C13—C15—H40	109.5	B18—B14—H15	124 (2)
H41—C15—H40	109.5	B13—B14—H15	125 (2)
C13—C15—H42	109.5	B12—B14—H15	118 (2)
H41—C15—H42	109.5	B19—B14—H15	121 (2)
H40—C15—H42	109.5	C10—B15—B20	104.7 (3)
N4—C16—C18	108.4 (4)	C10—B15—B19	104.5 (3)
N4—C16—C17	108.5 (4)	B20—B15—B19	60.4 (2)
C18—C16—C17	111.7 (4)	C10—B15—B17	59.5 (2)
N4—C16—H43	109.4	B20—B15—B17	59.3 (2)
C18—C16—H43	109.4	B19—B15—B17	107.9 (3)
C17—C16—H43	109.4	C10—B15—B12	58.3 (2)
C16—C17—H45	109.5	B20—B15—B12	108.0 (3)
C16—C17—H46	109.5	B19—B15—B12	59.8 (2)
H45—C17—H46	109.5	B17—B15—B12	108.2 (3)
C16—C17—H44	109.5	C10—B15—H16	119 (2)
H45—C17—H44	109.5	B20—B15—H16	127 (2)
H46—C17—H44	109.5	B19—B15—H16	127 (2)
C16—C18—H47	109.5	B17—B15—H16	120 (2)
C16—C18—H49	109.5	B12—B15—H16	119 (2)
H47—C18—H49	109.5	C11—B16—B20	105.1 (3)
C16—C18—H48	109.5	C11—B16—B17	59.4 (2)
H47—C18—H48	109.5	B20—B16—B17	59.6 (2)
H49—C18—H48	109.5	C11—B16—B13	58.6 (2)
C2—B1—C1	58.53 (19)	B20—B16—B13	108.6 (3)
C2—B1—B6	58.2 (2)	B17—B16—B13	108.6 (3)
C1—B1—B6	105.8 (3)	C11—B16—B18	104.8 (3)
C2—B1—B7	104.2 (3)	B20—B16—B18	60.6 (2)
C1—B1—B7	105.1 (3)	B17—B16—B18	108.3 (3)
B6—B1—B7	59.9 (2)	B13—B16—B18	60.0 (2)
C2—B1—B5	105.1 (2)	C11—B16—H17	116 (2)
C1—B1—B5	58.6 (2)	B20—B16—H17	128 (2)
B6—B1—B5	107.8 (3)	B17—B16—H17	117 (2)
B7—B1—B5	59.8 (2)	B13—B16—H17	119 (2)
C2—B1—H2	115 (2)	B18—B16—H17	130 (2)
C1—B1—H2	116 (2)	C11—B17—C10	56.99 (19)
B6—B1—H2	123 (2)	C11—B17—B20	104.9 (3)
B7—B1—H2	133 (2)	C10—B17—B20	104.4 (3)
B5—B1—H2	126 (2)	C11—B17—B16	58.8 (2)
C2—B2—C1	58.29 (19)	C10—B17—B16	104.4 (2)
C2—B2—B8	104.1 (3)	B20—B17—B16	60.0 (2)
C1—B2—B8	105.2 (3)	C11—B17—B15	104.5 (3)
C2—B2—B4	104.9 (3)	C10—B17—B15	58.3 (2)
C1—B2—B4	58.6 (2)	B20—B17—B15	60.0 (2)
B8—B2—B4	60.2 (2)	B16—B17—B15	107.8 (3)
C2—B2—B3	57.8 (2)	C11—B17—H18	117 (2)
C1—B2—B3	105.2 (3)	C10—B17—H18	117 (2)
B8—B2—B3	59.8 (2)	B20—B17—H18	132 (2)
B4—B2—B3	107.8 (3)	B16—B17—H18	125 (2)
C2—B2—H3	114.4 (19)	B15—B17—H18	124 (2)
C1—B2—H3	117 (2)	B19—B18—B14	60.8 (2)
B8—B2—H3	133.0 (19)	B19—B18—B13	108.4 (3)
B4—B2—H3	128 (2)	B14—B18—B13	60.0 (2)
B3—B2—H3	121 (2)	B19—B18—B16	107.7 (3)
C2—B3—B8	105.3 (3)	B14—B18—B16	108.1 (3)
C2—B3—B9	104.8 (3)	B13—B18—B16	60.0 (2)
B8—B3—B9	60.4 (2)	B19—B18—B20	60.1 (2)
C2—B3—B2	59.2 (2)	B14—B18—B20	108.6 (3)
B8—B3—B2	59.9 (2)	B13—B18—B20	107.5 (3)
B9—B3—B2	108.2 (3)	B16—B18—B20	59.3 (2)
C2—B3—B6	58.4 (2)	B19—B18—H19	124 (2)
B8—B3—B6	108.4 (3)	B14—B18—H19	124 (2)
B9—B3—B6	60.1 (2)	B13—B18—H19	121 (2)
B2—B3—B6	108.1 (3)	B16—B18—H19	119 (2)
C2—B3—H4	118 (2)	B20—B18—H19	120 (2)
B8—B3—H4	125 (2)	B18—B19—B15	108.3 (3)
B9—B3—H4	130 (2)	B18—B19—B12	107.8 (3)
B2—B3—H4	116 (2)	B15—B19—B12	60.5 (2)
B6—B3—H4	122 (2)	B18—B19—B20	60.4 (2)
C1—B4—B10	104.9 (2)	B15—B19—B20	59.7 (2)
C1—B4—B2	59.26 (19)	B12—B19—B20	108.0 (3)
B10—B4—B2	108.0 (3)	B18—B19—B14	59.7 (2)
C1—B4—B8	105.4 (3)	B15—B19—B14	108.3 (3)
B10—B4—B8	60.3 (2)	B12—B19—B14	59.8 (2)
B2—B4—B8	59.8 (2)	B20—B19—B14	107.9 (3)
C1—B4—B5	58.66 (19)	B18—B19—H20	126 (2)
B10—B4—B5	59.8 (2)	B15—B19—H20	118 (2)
B2—B4—B5	108.1 (2)	B12—B19—H20	119 (2)
B8—B4—B5	108.2 (3)	B20—B19—H20	123 (2)
C1—B4—H5	116 (2)	B14—B19—H20	123 (2)
B10—B4—H5	129 (2)	B17—B20—B16	60.3 (2)
B2—B4—H5	118 (2)	B17—B20—B15	60.7 (2)
B8—B4—H5	128 (2)	B16—B20—B15	108.6 (3)
B5—B4—H5	119 (2)	B17—B20—B19	108.6 (3)
C1—B5—B10	104.7 (3)	B16—B20—B19	107.9 (3)
C1—B5—B7	105.2 (3)	B15—B20—B19	59.9 (2)
B10—B5—B7	60.5 (2)	B17—B20—B18	108.5 (3)
C1—B5—B4	58.6 (2)	B16—B20—B18	60.1 (2)
B10—B5—B4	59.6 (2)	B15—B20—B18	107.7 (3)
B7—B5—B4	108.1 (3)	B19—B20—B18	59.5 (2)
C1—B5—B1	58.7 (2)	B17—B20—H21	121 (2)
B10—B5—B1	107.8 (3)	B16—B20—H21	122 (2)
B7—B5—B1	59.9 (2)	B15—B20—H21	121 (2)
B4—B5—B1	107.4 (3)	B19—B20—H21	122 (2)
C1—B5—H6	119 (2)	B18—B20—H21	123 (2)
B10—B5—H6	130 (2)	C3—N1—C4	119.1 (3)
B7—B5—H6	123 (2)	C3—N1—SN	121.5 (2)
B4—B5—H6	125 (2)	C4—N1—SN	119.0 (2)
B1—B5—H6	115 (2)	C3—N2—C7	130.0 (3)
C2—B6—B7	105.2 (3)	C12—N3—C13	121.9 (3)
C2—B6—B1	59.0 (2)	C12—N3—SN	120.1 (2)
B7—B6—B1	60.2 (2)	C13—N3—SN	117.8 (2)
C2—B6—B9	104.3 (3)	C12—N4—C16	135.6 (3)
B7—B6—B9	60.4 (2)	C12—N4—H1	113 (3)
B1—B6—B9	108.3 (3)	C16—N4—H1	111 (3)
C2—B6—B3	58.0 (2)	N1—SN—C11	100.52 (12)
B7—B6—B3	108.1 (3)	N1—SN—C2	82.93 (11)
B1—B6—B3	107.7 (3)	C11—SN—C2	124.32 (12)
B9—B6—B3	59.8 (2)	N1—SN—N3	176.28 (10)
C2—B6—H7	120 (2)	C11—SN—N3	77.66 (11)
B7—B6—H7	125 (2)	C2—SN—N3	95.39 (11)
B1—B6—H7	119 (2)	N1—SN—CL	95.78 (8)
B9—B6—H7	127 (2)	C11—SN—CL	115.59 (8)
B3—B6—H7	121 (2)	C2—SN—CL	119.30 (9)
B6—B7—B5	108.3 (3)	N3—SN—CL	87.93 (7)
			
C3—C1—C2—B3	−146.1 (3)	B3—B8—B10—B9	−37.6 (3)
B4—C1—C2—B3	0.5 (3)	B2—B8—B10—B9	−101.1 (3)
B5—C1—C2—B3	67.6 (3)	B4—B8—B10—B9	−138.6 (3)
B1—C1—C2—B3	107.8 (3)	B3—B8—B10—B7	−0.1 (4)
B2—C1—C2—B3	−39.1 (3)	B2—B8—B10—B7	−63.6 (3)
C3—C1—C2—B6	144.8 (3)	B4—B8—B10—B7	−101.1 (3)
B4—C1—C2—B6	−68.6 (3)	B9—B8—B10—B7	37.5 (3)
B5—C1—C2—B6	−1.5 (3)	B6—B7—B10—B4	−63.7 (4)
B1—C1—C2—B6	38.7 (2)	B5—B7—B10—B4	37.7 (3)
B2—C1—C2—B6	−108.2 (3)	B1—B7—B10—B4	−0.5 (4)
C3—C1—C2—B1	106.1 (3)	B9—B7—B10—B4	−101.2 (3)
B4—C1—C2—B1	−107.3 (3)	B6—B7—B10—B5	−101.4 (3)
B5—C1—C2—B1	−40.2 (2)	B1—B7—B10—B5	−38.2 (3)
B2—C1—C2—B1	−147.0 (3)	B9—B7—B10—B5	−138.9 (3)
C3—C1—C2—B2	−107.0 (3)	B6—B7—B10—B9	37.6 (3)
B4—C1—C2—B2	39.7 (2)	B5—B7—B10—B9	138.9 (3)
B5—C1—C2—B2	106.8 (3)	B1—B7—B10—B9	100.7 (3)
B1—C1—C2—B2	147.0 (3)	B6—B7—B10—B8	0.0 (4)
C3—C1—C2—SN	−1.6 (3)	B5—B7—B10—B8	101.4 (3)
B4—C1—C2—SN	145.1 (2)	B1—B7—B10—B8	63.2 (4)
B5—C1—C2—SN	−147.8 (2)	B9—B7—B10—B8	−37.5 (3)
B1—C1—C2—SN	−107.6 (2)	C12—C10—B11—C11	103.0 (3)
B2—C1—C2—SN	105.4 (2)	B12—C10—B11—C11	−138.3 (3)
C2—C1—C3—N2	179.5 (3)	B15—C10—B11—C11	−101.4 (3)
B4—C1—C3—N2	38.5 (5)	B17—C10—B11—C11	−32.4 (3)
B5—C1—C3—N2	−39.6 (5)	C12—C10—B11—B14	−158.6 (3)
B1—C1—C3—N2	−112.6 (4)	C11—C10—B11—B14	98.4 (3)
B2—C1—C3—N2	111.4 (4)	B12—C10—B11—B14	−39.8 (3)
C2—C1—C3—N1	−2.2 (4)	B15—C10—B11—B14	−3.0 (4)
B4—C1—C3—N1	−143.2 (3)	B17—C10—B11—B14	66.0 (3)
B5—C1—C3—N1	138.7 (3)	C12—C10—B11—B13	139.1 (3)
B1—C1—C3—N1	65.7 (4)	C11—C10—B11—B13	36.1 (2)
B2—C1—C3—N1	−70.3 (3)	B12—C10—B11—B13	−102.2 (3)
C12—C10—C11—B13	−147.7 (3)	B15—C10—B11—B13	−65.4 (3)
B12—C10—C11—B13	0.0 (3)	B17—C10—B11—B13	3.6 (4)
B15—C10—C11—B13	68.0 (3)	C12—C10—B11—B12	−118.7 (3)
B11—C10—C11—B13	−39.3 (3)	C11—C10—B11—B12	138.3 (3)
B17—C10—C11—B13	106.7 (3)	B15—C10—B11—B12	36.8 (3)
C12—C10—C11—B16	145.0 (3)	B17—C10—B11—B12	105.8 (3)
B12—C10—C11—B16	−67.4 (3)	B13—C11—B11—C10	138.1 (3)
B15—C10—C11—B16	0.6 (3)	B16—C11—B11—C10	101.2 (3)
B11—C10—C11—B16	−106.7 (3)	B17—C11—B11—C10	32.7 (3)
B17—C10—C11—B16	39.3 (3)	SN—C11—B11—C10	−102.8 (2)
C12—C10—C11—B11	−108.3 (3)	C10—C11—B11—B14	−98.4 (3)
B12—C10—C11—B11	39.3 (3)	B13—C11—B11—B14	39.7 (3)
B15—C10—C11—B11	107.3 (3)	B16—C11—B11—B14	2.9 (4)
B17—C10—C11—B11	146.0 (3)	B17—C11—B11—B14	−65.6 (3)
C12—C10—C11—B17	105.7 (3)	SN—C11—B11—B14	158.9 (2)
B12—C10—C11—B17	−106.7 (3)	C10—C11—B11—B13	−138.1 (3)
B15—C10—C11—B17	−38.7 (2)	B16—C11—B11—B13	−36.8 (3)
B11—C10—C11—B17	−146.0 (3)	B17—C11—B11—B13	−105.3 (3)
C12—C10—C11—SN	−0.9 (3)	SN—C11—B11—B13	119.2 (3)
B12—C10—C11—SN	146.7 (2)	C10—C11—B11—B12	−35.9 (2)
B15—C10—C11—SN	−145.3 (2)	B13—C11—B11—B12	102.2 (3)
B11—C10—C11—SN	107.4 (2)	B16—C11—B11—B12	65.4 (3)
B17—C10—C11—SN	−106.6 (2)	B17—C11—B11—B12	−3.1 (4)
C11—C10—C12—N3	−5.0 (4)	SN—C11—B11—B12	−138.6 (2)
B12—C10—C12—N3	−146.9 (3)	C12—C10—B12—B19	−153.7 (3)
B15—C10—C12—N3	133.9 (3)	C11—C10—B12—B19	63.8 (3)
B11—C10—C12—N3	−73.1 (4)	B15—C10—B12—B19	−39.6 (3)
B17—C10—C12—N3	62.4 (4)	B11—C10—B12—B19	102.6 (3)
C11—C10—C12—N4	176.2 (3)	B17—C10—B12—B19	−3.0 (4)
B12—C10—C12—N4	34.3 (5)	C12—C10—B12—B14	143.3 (3)
B15—C10—C12—N4	−44.8 (5)	C11—C10—B12—B14	0.8 (3)
B11—C10—C12—N4	108.1 (4)	B15—C10—B12—B14	−102.6 (3)
B17—C10—C12—N4	−116.4 (4)	B11—C10—B12—B14	39.6 (3)
B3—C2—B1—C1	−101.8 (3)	B17—C10—B12—B14	−66.1 (3)
B6—C2—B1—C1	−139.1 (3)	C12—C10—B12—B11	103.7 (3)
B2—C2—B1—C1	−31.6 (3)	C11—C10—B12—B11	−38.8 (3)
SN—C2—B1—C1	96.7 (2)	B15—C10—B12—B11	−142.2 (3)
C1—C2—B1—B6	139.1 (3)	B17—C10—B12—B11	−105.7 (3)
B3—C2—B1—B6	37.2 (3)	C12—C10—B12—B15	−114.0 (4)
B2—C2—B1—B6	107.5 (3)	C11—C10—B12—B15	103.5 (3)
SN—C2—B1—B6	−124.2 (2)	B11—C10—B12—B15	142.2 (3)
C1—C2—B1—B7	99.1 (3)	B17—C10—B12—B15	36.6 (3)
B3—C2—B1—B7	−2.7 (3)	C11—B11—B12—C10	35.5 (2)
B6—C2—B1—B7	−39.9 (2)	B14—B11—B12—C10	134.5 (3)
B2—C2—B1—B7	67.6 (3)	B13—B11—B12—C10	96.5 (3)
SN—C2—B1—B7	−164.1 (2)	C10—B11—B12—B19	−96.6 (3)
C1—C2—B1—B5	37.2 (2)	C11—B11—B12—B19	−61.2 (3)
B3—C2—B1—B5	−64.7 (3)	B14—B11—B12—B19	37.8 (3)
B6—C2—B1—B5	−101.9 (3)	B13—B11—B12—B19	−0.1 (3)
B2—C2—B1—B5	5.6 (4)	C10—B11—B12—B14	−134.5 (3)
SN—C2—B1—B5	133.9 (2)	C11—B11—B12—B14	−99.0 (3)
C3—C1—B1—C2	−106.3 (3)	B13—B11—B12—B14	−37.9 (3)
B4—C1—B1—C2	99.6 (3)	C10—B11—B12—B15	−33.4 (3)
B5—C1—B1—C2	136.9 (3)	C11—B11—B12—B15	2.0 (4)
B2—C1—B1—C2	31.0 (2)	B14—B11—B12—B15	101.0 (3)
C3—C1—B1—B6	−141.6 (3)	B13—B11—B12—B15	63.1 (3)
C2—C1—B1—B6	−35.3 (2)	C10—C11—B13—B14	−0.8 (3)
B4—C1—B1—B6	64.2 (3)	B16—C11—B13—B14	102.3 (3)
B5—C1—B1—B6	101.5 (3)	B11—C11—B13—B14	−39.6 (3)
B2—C1—B1—B6	−4.3 (3)	B17—C11—B13—B14	66.6 (3)
C3—C1—B1—B7	156.0 (3)	SN—C11—B13—B14	−142.0 (3)
C2—C1—B1—B7	−97.7 (3)	C10—C11—B13—B16	−103.1 (3)
B4—C1—B1—B7	1.9 (4)	B11—C11—B13—B16	−141.9 (3)
B5—C1—B1—B7	39.2 (3)	B17—C11—B13—B16	−35.7 (3)
B2—C1—B1—B7	−66.7 (3)	SN—C11—B13—B16	115.7 (3)
C3—C1—B1—B5	116.8 (3)	C10—C11—B13—B18	−63.1 (3)
C2—C1—B1—B5	−136.9 (3)	B16—C11—B13—B18	40.0 (3)
B4—C1—B1—B5	−37.3 (3)	B11—C11—B13—B18	−101.9 (3)
B2—C1—B1—B5	−105.9 (3)	B17—C11—B13—B18	4.3 (4)
B3—C2—B2—C1	138.9 (3)	SN—C11—B13—B18	155.7 (2)
B6—C2—B2—C1	101.5 (3)	C10—C11—B13—B11	38.8 (2)
B1—C2—B2—C1	31.5 (3)	B16—C11—B13—B11	141.9 (3)
SN—C2—B2—C1	−97.9 (2)	B17—C11—B13—B11	106.2 (3)
C1—C2—B2—B8	−99.3 (3)	SN—C11—B13—B11	−102.4 (3)
B3—C2—B2—B8	39.5 (3)	C10—B11—B13—C11	−35.6 (2)
B6—C2—B2—B8	2.2 (4)	B14—B11—B13—C11	−134.6 (3)
B1—C2—B2—B8	−67.9 (3)	B12—B11—B13—C11	−96.6 (3)
SN—C2—B2—B8	162.8 (2)	C10—B11—B13—B14	99.0 (3)
C1—C2—B2—B4	−37.0 (2)	C11—B11—B13—B14	134.6 (3)
B3—C2—B2—B4	101.9 (3)	B12—B11—B13—B14	38.0 (2)
B6—C2—B2—B4	64.5 (3)	C10—B11—B13—B16	−1.8 (4)
B1—C2—B2—B4	−5.5 (4)	C11—B11—B13—B16	33.8 (3)
SN—C2—B2—B4	−134.8 (2)	B14—B11—B13—B16	−100.7 (3)
C1—C2—B2—B3	−138.9 (3)	B12—B11—B13—B16	−62.8 (3)
B6—C2—B2—B3	−37.4 (3)	C10—B11—B13—B18	61.7 (3)
B1—C2—B2—B3	−107.4 (3)	C11—B11—B13—B18	97.3 (3)
SN—C2—B2—B3	123.3 (2)	B14—B11—B13—B18	−37.3 (3)
C3—C1—B2—C2	105.9 (3)	B12—B11—B13—B18	0.7 (3)
B4—C1—B2—C2	−137.1 (3)	C10—B11—B14—B18	−61.1 (4)
B5—C1—B2—C2	−100.2 (3)	C11—B11—B14—B18	−1.4 (4)
B1—C1—B2—C2	−31.1 (2)	B13—B11—B14—B18	37.5 (3)
C3—C1—B2—B8	−156.7 (3)	B12—B11—B14—B18	−100.1 (3)
C2—C1—B2—B8	97.5 (3)	C10—B11—B14—B13	−98.6 (3)
B4—C1—B2—B8	−39.6 (2)	C11—B11—B14—B13	−38.9 (2)
B5—C1—B2—B8	−2.7 (3)	B12—B11—B14—B13	−137.6 (3)
B1—C1—B2—B8	66.4 (3)	C10—B11—B14—B12	39.0 (3)
C3—C1—B2—B4	−117.0 (3)	C11—B11—B14—B12	98.6 (3)
C2—C1—B2—B4	137.1 (3)	B13—B11—B14—B12	137.6 (3)
B5—C1—B2—B4	36.9 (3)	C10—B11—B14—B19	1.8 (4)
B1—C1—B2—B4	106.0 (3)	C11—B11—B14—B19	61.4 (4)
C3—C1—B2—B3	141.1 (3)	B13—B11—B14—B19	100.4 (3)
C2—C1—B2—B3	35.2 (2)	B12—B11—B14—B19	−37.2 (3)
B4—C1—B2—B3	−101.9 (3)	C11—B13—B14—B11	39.3 (2)
B5—C1—B2—B3	−64.9 (3)	B16—B13—B14—B11	100.9 (3)
B1—C1—B2—B3	4.2 (3)	B18—B13—B14—B11	138.2 (3)
C1—C2—B3—B8	−1.5 (3)	C11—B13—B14—B18	−98.9 (3)
B6—C2—B3—B8	102.5 (3)	B16—B13—B14—B18	−37.2 (3)
B1—C2—B3—B8	65.6 (3)	B11—B13—B14—B18	−138.2 (3)
B2—C2—B3—B8	−39.8 (3)	C11—B13—B14—B12	1.3 (4)
SN—C2—B3—B8	−136.3 (3)	B16—B13—B14—B12	62.9 (4)
C1—C2—B3—B9	−64.2 (3)	B18—B13—B14—B12	100.2 (3)
B6—C2—B3—B9	39.7 (3)	B11—B13—B14—B12	−38.0 (3)
B1—C2—B3—B9	2.8 (4)	C11—B13—B14—B19	−61.5 (3)
B2—C2—B3—B9	−102.6 (3)	B16—B13—B14—B19	0.1 (4)
SN—C2—B3—B9	160.9 (2)	B18—B13—B14—B19	37.4 (3)
C1—C2—B3—B2	38.4 (2)	B11—B13—B14—B19	−100.8 (3)
B6—C2—B3—B2	142.3 (3)	C10—B12—B14—B11	−39.3 (2)
B1—C2—B3—B2	105.5 (3)	B19—B12—B14—B11	−138.1 (3)
SN—C2—B3—B2	−96.5 (3)	B15—B12—B14—B11	−100.8 (3)
C1—C2—B3—B6	−103.9 (3)	C10—B12—B14—B18	62.1 (3)
B1—C2—B3—B6	−36.9 (3)	B19—B12—B14—B18	−36.7 (3)
B2—C2—B3—B6	−142.3 (3)	B11—B12—B14—B18	101.4 (3)
SN—C2—B3—B6	121.2 (3)	B15—B12—B14—B18	0.6 (4)
C1—B2—B3—C2	−35.4 (2)	C10—B12—B14—B13	−1.3 (4)
B8—B2—B3—C2	−134.4 (3)	B19—B12—B14—B13	−100.1 (3)
B4—B2—B3—C2	−96.8 (3)	B11—B12—B14—B13	38.0 (3)
C2—B2—B3—B8	134.4 (3)	B15—B12—B14—B13	−62.8 (4)
C1—B2—B3—B8	99.0 (3)	C10—B12—B14—B19	98.8 (3)
B4—B2—B3—B8	37.7 (3)	B11—B12—B14—B19	138.1 (3)
C2—B2—B3—B9	96.7 (3)	B15—B12—B14—B19	37.3 (3)
C1—B2—B3—B9	61.3 (3)	C12—C10—B15—B20	−141.1 (3)
B8—B2—B3—B9	−37.7 (3)	C11—C10—B15—B20	−1.5 (3)
B4—B2—B3—B9	0.0 (3)	B12—C10—B15—B20	102.3 (3)
C2—B2—B3—B6	33.2 (2)	B11—C10—B15—B20	65.6 (3)
C1—B2—B3—B6	−2.3 (3)	B17—C10—B15—B20	−39.8 (3)
B8—B2—B3—B6	−101.3 (3)	C12—C10—B15—B19	156.3 (3)
B4—B2—B3—B6	−63.6 (3)	C11—C10—B15—B19	−64.1 (3)
C3—C1—B4—B10	−153.7 (3)	B12—C10—B15—B19	39.6 (3)
C2—C1—B4—B10	63.4 (3)	B11—C10—B15—B19	2.9 (4)
B5—C1—B4—B10	−39.5 (3)	B17—C10—B15—B19	−102.4 (3)
B1—C1—B4—B10	−2.2 (4)	C12—C10—B15—B17	−101.3 (3)
B2—C1—B4—B10	102.3 (3)	C11—C10—B15—B17	38.3 (2)
C3—C1—B4—B2	104.1 (3)	B12—C10—B15—B17	142.0 (3)
C2—C1—B4—B2	−38.9 (2)	B11—C10—B15—B17	105.4 (3)
B5—C1—B4—B2	−141.8 (3)	C12—C10—B15—B12	116.6 (4)
B1—C1—B4—B2	−104.5 (3)	C11—C10—B15—B12	−103.8 (3)
C3—C1—B4—B8	143.6 (3)	B11—C10—B15—B12	−36.7 (3)
C2—C1—B4—B8	0.6 (3)	B17—C10—B15—B12	−142.0 (3)
B5—C1—B4—B8	−102.2 (3)	B19—B12—B15—C10	134.4 (3)
B1—C1—B4—B8	−64.9 (3)	B14—B12—B15—C10	96.7 (3)
B2—C1—B4—B8	39.5 (3)	B11—B12—B15—C10	33.5 (3)
C3—C1—B4—B5	−114.2 (3)	C10—B12—B15—B20	−96.6 (3)
C2—C1—B4—B5	102.9 (3)	B19—B12—B15—B20	37.9 (3)
B1—C1—B4—B5	37.3 (3)	B14—B12—B15—B20	0.1 (4)
B2—C1—B4—B5	141.8 (3)	B11—B12—B15—B20	−63.0 (4)
C2—B2—B4—C1	36.8 (2)	C10—B12—B15—B19	−134.4 (3)
B8—B2—B4—C1	134.8 (3)	B14—B12—B15—B19	−37.7 (3)
B3—B2—B4—C1	97.3 (3)	B11—B12—B15—B19	−100.9 (3)
C2—B2—B4—B10	−60.2 (3)	C10—B12—B15—B17	−33.9 (3)
C1—B2—B4—B10	−97.0 (3)	B19—B12—B15—B17	100.5 (3)
B8—B2—B4—B10	37.7 (2)	B14—B12—B15—B17	62.8 (4)
B3—B2—B4—B10	0.2 (3)	B11—B12—B15—B17	−0.4 (4)
C2—B2—B4—B8	−97.9 (3)	C10—C11—B16—B20	0.6 (3)
C1—B2—B4—B8	−134.8 (3)	B13—C11—B16—B20	−102.9 (3)
B3—B2—B4—B8	−37.5 (2)	B11—C11—B16—B20	−66.1 (3)
C2—B2—B4—B5	3.1 (3)	B17—C11—B16—B20	39.9 (3)
C1—B2—B4—B5	−33.8 (2)	SN—C11—B16—B20	140.9 (2)
B8—B2—B4—B5	101.0 (3)	C10—C11—B16—B17	−39.3 (3)
B3—B2—B4—B5	63.5 (3)	B13—C11—B16—B17	−142.7 (3)
C3—C1—B5—B10	154.3 (3)	B11—C11—B16—B17	−105.9 (3)
C2—C1—B5—B10	−62.9 (3)	SN—C11—B16—B17	101.0 (3)
B4—C1—B5—B10	39.3 (2)	C10—C11—B16—B13	103.5 (3)
B1—C1—B5—B10	−102.2 (3)	B11—C11—B16—B13	36.8 (3)
B2—C1—B5—B10	2.6 (3)	B17—C11—B16—B13	142.7 (3)
C3—C1—B5—B7	−142.9 (3)	SN—C11—B16—B13	−116.2 (3)
C2—C1—B5—B7	−0.1 (3)	C10—C11—B16—B18	63.6 (3)
B4—C1—B5—B7	102.2 (3)	B13—C11—B16—B18	−39.9 (3)
B1—C1—B5—B7	−39.3 (2)	B11—C11—B16—B18	−3.1 (4)
B2—C1—B5—B7	65.5 (3)	B17—C11—B16—B18	102.8 (3)
C3—C1—B5—B4	114.9 (3)	SN—C11—B16—B18	−156.2 (2)
C2—C1—B5—B4	−102.3 (3)	B14—B13—B16—C11	−97.1 (3)
B1—C1—B5—B4	−141.5 (3)	B18—B13—B16—C11	−134.2 (3)
B2—C1—B5—B4	−36.7 (3)	B11—B13—B16—C11	−33.9 (3)
C3—C1—B5—B1	−103.6 (3)	C11—B13—B16—B20	96.7 (3)
C2—C1—B5—B1	39.2 (2)	B14—B13—B16—B20	−0.4 (4)
B4—C1—B5—B1	141.5 (3)	B18—B13—B16—B20	−37.6 (3)
B2—C1—B5—B1	104.8 (3)	B11—B13—B16—B20	62.8 (4)
B10—B4—B5—C1	134.7 (3)	C11—B13—B16—B17	33.4 (3)
B2—B4—B5—C1	34.0 (2)	B14—B13—B16—B17	−63.7 (4)
B8—B4—B5—C1	97.3 (3)	B18—B13—B16—B17	−100.9 (3)
C1—B4—B5—B10	−134.7 (3)	B11—B13—B16—B17	−0.5 (4)
B2—B4—B5—B10	−100.7 (3)	C11—B13—B16—B18	134.2 (3)
B8—B4—B5—B10	−37.4 (3)	B14—B13—B16—B18	37.2 (3)
C1—B4—B5—B7	−97.1 (3)	B11—B13—B16—B18	100.3 (3)
B10—B4—B5—B7	37.5 (3)	B13—C11—B17—C10	−101.8 (3)
B2—B4—B5—B7	−63.1 (3)	B16—C11—B17—C10	−137.8 (3)
B8—B4—B5—B7	0.2 (3)	B11—C11—B17—C10	−32.5 (3)
C1—B4—B5—B1	−33.9 (2)	SN—C11—B17—C10	103.3 (2)
B10—B4—B5—B1	100.8 (3)	C10—C11—B17—B20	97.7 (3)
B2—B4—B5—B1	0.1 (4)	B13—C11—B17—B20	−4.0 (4)
B8—B4—B5—B1	63.4 (3)	B16—C11—B17—B20	−40.0 (3)
C2—B1—B5—C1	−37.1 (2)	B11—C11—B17—B20	65.2 (4)
B6—B1—B5—C1	−98.0 (3)	SN—C11—B17—B20	−158.9 (2)
B7—B1—B5—C1	−135.1 (3)	C10—C11—B17—B16	137.8 (3)
C2—B1—B5—B10	59.6 (3)	B13—C11—B17—B16	36.0 (3)
C1—B1—B5—B10	96.7 (3)	B11—C11—B17—B16	105.2 (3)
B6—B1—B5—B10	−1.3 (4)	SN—C11—B17—B16	−118.9 (3)
B7—B1—B5—B10	−38.3 (3)	C10—C11—B17—B15	35.5 (2)
C2—B1—B5—B7	97.9 (3)	B13—C11—B17—B15	−66.3 (3)
C1—B1—B5—B7	135.1 (3)	B16—C11—B17—B15	−102.3 (3)
B6—B1—B5—B7	37.1 (3)	B11—C11—B17—B15	2.9 (4)
C2—B1—B5—B4	−3.3 (3)	SN—C11—B17—B15	138.8 (2)
C1—B1—B5—B4	33.9 (2)	C12—C10—B17—C11	−104.0 (3)
B6—B1—B5—B4	−64.1 (3)	B12—C10—B17—C11	101.8 (3)
B7—B1—B5—B4	−101.2 (3)	B15—C10—B17—C11	138.7 (3)
C1—C2—B6—B7	2.3 (3)	B11—C10—B17—C11	32.5 (3)
B3—C2—B6—B7	−102.1 (3)	C12—C10—B17—B20	157.4 (3)
B1—C2—B6—B7	40.3 (2)	C11—C10—B17—B20	−98.6 (3)
B2—C2—B6—B7	−65.0 (3)	B12—C10—B17—B20	3.2 (4)
SN—C2—B6—B7	138.7 (3)	B15—C10—B17—B20	40.1 (3)
C1—C2—B6—B1	−38.0 (2)	B11—C10—B17—B20	−66.1 (3)
B3—C2—B6—B1	−142.4 (3)	C12—C10—B17—B16	−140.4 (3)
B2—C2—B6—B1	−105.3 (3)	C11—C10—B17—B16	−36.4 (2)
SN—C2—B6—B1	98.4 (3)	B12—C10—B17—B16	65.4 (3)
C1—C2—B6—B9	65.0 (3)	B15—C10—B17—B16	102.3 (3)
B3—C2—B6—B9	−39.5 (3)	B11—C10—B17—B16	−3.9 (4)
B1—C2—B6—B9	103.0 (3)	C12—C10—B17—B15	117.3 (3)
B2—C2—B6—B9	−2.3 (4)	C11—C10—B17—B15	−138.7 (3)
SN—C2—B6—B9	−158.7 (2)	B12—C10—B17—B15	−36.9 (3)
C1—C2—B6—B3	104.5 (3)	B11—C10—B17—B15	−106.2 (3)
B1—C2—B6—B3	142.4 (3)	B20—B16—B17—C11	−134.2 (3)
B2—C2—B6—B3	37.1 (3)	B13—B16—B17—C11	−33.0 (3)
SN—C2—B6—B3	−119.2 (3)	B18—B16—B17—C11	−96.7 (3)
C1—B1—B6—C2	35.5 (2)	C11—B16—B17—C10	35.6 (2)
B7—B1—B6—C2	134.0 (3)	B20—B16—B17—C10	−98.6 (3)
B5—B1—B6—C2	97.0 (3)	B13—B16—B17—C10	2.6 (4)
C2—B1—B6—B7	−134.0 (3)	B18—B16—B17—C10	−61.1 (3)
C1—B1—B6—B7	−98.5 (3)	C11—B16—B17—B20	134.2 (3)
B5—B1—B6—B7	−37.0 (3)	B13—B16—B17—B20	101.1 (3)
C2—B1—B6—B9	−96.0 (3)	B18—B16—B17—B20	37.5 (3)
C1—B1—B6—B9	−60.5 (3)	C11—B16—B17—B15	96.5 (3)
B7—B1—B6—B9	38.0 (3)	B20—B16—B17—B15	−37.7 (3)
B5—B1—B6—B9	1.0 (4)	B13—B16—B17—B15	63.4 (3)
C2—B1—B6—B3	−32.9 (2)	B18—B16—B17—B15	−0.2 (4)
C1—B1—B6—B3	2.6 (3)	C10—B15—B17—C11	−34.9 (2)
B7—B1—B6—B3	101.1 (3)	B20—B15—B17—C11	99.1 (3)
B5—B1—B6—B3	64.1 (3)	B19—B15—B17—C11	61.8 (3)
B8—B3—B6—C2	−97.0 (3)	B12—B15—B17—C11	−1.4 (4)
B9—B3—B6—C2	−134.5 (3)	B20—B15—B17—C10	134.0 (3)
B2—B3—B6—C2	−33.5 (2)	B19—B15—B17—C10	96.6 (3)
C2—B3—B6—B7	96.9 (3)	B12—B15—B17—C10	33.4 (3)
B8—B3—B6—B7	−0.1 (4)	C10—B15—B17—B20	−134.0 (3)
B9—B3—B6—B7	−37.6 (3)	B19—B15—B17—B20	−37.3 (3)
B2—B3—B6—B7	63.4 (3)	B12—B15—B17—B20	−100.5 (3)
C2—B3—B6—B1	33.3 (2)	C10—B15—B17—B16	−96.2 (3)
B8—B3—B6—B1	−63.7 (3)	B20—B15—B17—B16	37.7 (3)
B9—B3—B6—B1	−101.2 (3)	B19—B15—B17—B16	0.4 (4)
B2—B3—B6—B1	−0.2 (3)	B12—B15—B17—B16	−62.8 (3)
C2—B3—B6—B9	134.5 (3)	B11—B14—B18—B19	100.2 (3)
B8—B3—B6—B9	37.6 (3)	B13—B14—B18—B19	137.8 (3)
B2—B3—B6—B9	101.0 (3)	B12—B14—B18—B19	36.7 (3)
C2—B6—B7—B5	−2.3 (3)	B11—B14—B18—B13	−37.6 (3)
B1—B6—B7—B5	37.4 (2)	B12—B14—B18—B13	−101.1 (3)
B9—B6—B7—B5	−100.4 (3)	B19—B14—B18—B13	−137.8 (3)
B3—B6—B7—B5	−63.0 (3)	B11—B14—B18—B16	−0.4 (4)
C2—B6—B7—B1	−39.7 (2)	B13—B14—B18—B16	37.2 (3)
B9—B6—B7—B1	−137.8 (3)	B12—B14—B18—B16	−63.9 (4)
B3—B6—B7—B1	−100.4 (3)	B19—B14—B18—B16	−100.6 (3)
C2—B6—B7—B10	60.7 (3)	B11—B14—B18—B20	62.4 (4)
B1—B6—B7—B10	100.4 (3)	B13—B14—B18—B20	100.0 (3)
B9—B6—B7—B10	−37.3 (3)	B12—B14—B18—B20	−1.1 (4)
B3—B6—B7—B10	0.0 (4)	B19—B14—B18—B20	−37.8 (3)
C2—B6—B7—B9	98.0 (3)	C11—B13—B18—B19	60.8 (4)
B1—B6—B7—B9	137.8 (3)	B14—B13—B18—B19	−38.2 (3)
B3—B6—B7—B9	37.4 (3)	B16—B13—B18—B19	100.2 (3)
C1—B5—B7—B6	1.5 (3)	B11—B13—B18—B19	−1.0 (4)
B10—B5—B7—B6	100.0 (3)	C11—B13—B18—B14	99.0 (3)
B4—B5—B7—B6	62.9 (3)	B16—B13—B18—B14	138.4 (3)
B1—B5—B7—B6	−37.2 (3)	B11—B13—B18—B14	37.2 (3)
C1—B5—B7—B1	38.7 (2)	C11—B13—B18—B16	−39.4 (3)
B10—B5—B7—B1	137.2 (3)	B14—B13—B18—B16	−138.4 (3)
B4—B5—B7—B1	100.1 (3)	B11—B13—B18—B16	−101.2 (3)
C1—B5—B7—B10	−98.5 (3)	C11—B13—B18—B20	−2.7 (4)
B4—B5—B7—B10	−37.2 (3)	B14—B13—B18—B20	−101.7 (3)
B1—B5—B7—B10	−137.2 (3)	B16—B13—B18—B20	36.7 (3)
C1—B5—B7—B9	−62.0 (3)	B11—B13—B18—B20	−64.5 (4)
B10—B5—B7—B9	36.5 (3)	C11—B16—B18—B19	−62.2 (3)
B4—B5—B7—B9	−0.6 (3)	B20—B16—B18—B19	37.0 (3)
B1—B5—B7—B9	−100.7 (3)	B17—B16—B18—B19	−0.1 (4)
C2—B1—B7—B6	39.1 (2)	B13—B16—B18—B19	−101.4 (3)
C1—B1—B7—B6	99.8 (3)	C11—B16—B18—B14	2.0 (4)
B5—B1—B7—B6	138.4 (3)	B20—B16—B18—B14	101.3 (3)
C2—B1—B7—B5	−99.3 (3)	B17—B16—B18—B14	64.2 (3)
C1—B1—B7—B5	−38.7 (2)	B13—B16—B18—B14	−37.2 (3)
B6—B1—B7—B5	−138.4 (3)	C11—B16—B18—B13	39.2 (3)
C2—B1—B7—B10	−61.4 (3)	B20—B16—B18—B13	138.5 (3)
C1—B1—B7—B10	−0.8 (4)	B17—B16—B18—B13	101.4 (3)
B6—B1—B7—B10	−100.5 (3)	C11—B16—B18—B20	−99.3 (3)
B5—B1—B7—B10	37.9 (3)	B17—B16—B18—B20	−37.1 (3)
C2—B1—B7—B9	1.4 (3)	B13—B16—B18—B20	−138.5 (3)
C1—B1—B7—B9	62.1 (3)	B14—B18—B19—B15	−100.9 (3)
B6—B1—B7—B9	−37.7 (3)	B13—B18—B19—B15	−63.1 (4)
B5—B1—B7—B9	100.7 (3)	B16—B18—B19—B15	0.3 (4)
C2—B3—B8—B2	39.5 (2)	B20—B18—B19—B15	37.0 (3)
B9—B3—B8—B2	138.1 (3)	B14—B18—B19—B12	−36.9 (3)
B6—B3—B8—B2	100.7 (3)	B13—B18—B19—B12	0.9 (4)
C2—B3—B8—B4	1.8 (4)	B16—B18—B19—B12	64.3 (3)
B9—B3—B8—B4	100.4 (3)	B20—B18—B19—B12	101.0 (3)
B2—B3—B8—B4	−37.7 (3)	B14—B18—B19—B20	−137.9 (3)
B6—B3—B8—B4	63.0 (3)	B13—B18—B19—B20	−100.1 (3)
C2—B3—B8—B10	−61.1 (3)	B16—B18—B19—B20	−36.7 (3)
B9—B3—B8—B10	37.5 (3)	B13—B18—B19—B14	37.8 (3)
B2—B3—B8—B10	−100.6 (3)	B16—B18—B19—B14	101.2 (3)
B6—B3—B8—B10	0.1 (4)	B20—B18—B19—B14	137.9 (3)
C2—B3—B8—B9	−98.6 (3)	C10—B15—B19—B18	61.7 (3)
B2—B3—B8—B9	−138.1 (3)	B20—B15—B19—B18	−37.3 (3)
B6—B3—B8—B9	−37.4 (3)	B17—B15—B19—B18	−0.5 (4)
C2—B2—B8—B3	−38.5 (2)	B12—B15—B19—B18	100.6 (3)
C1—B2—B8—B3	−99.0 (3)	C10—B15—B19—B12	−38.9 (3)
B4—B2—B8—B3	−137.9 (3)	B20—B15—B19—B12	−137.8 (3)
C2—B2—B8—B4	99.3 (3)	B17—B15—B19—B12	−101.0 (3)
C1—B2—B8—B4	38.9 (2)	C10—B15—B19—B20	98.9 (3)
B3—B2—B8—B4	137.9 (3)	B17—B15—B19—B20	36.8 (3)
C2—B2—B8—B10	62.0 (3)	B12—B15—B19—B20	137.8 (3)
C1—B2—B8—B10	1.6 (3)	C10—B15—B19—B14	−1.6 (4)
B4—B2—B8—B10	−37.3 (2)	B20—B15—B19—B14	−100.5 (3)
B3—B2—B8—B10	100.6 (3)	B17—B15—B19—B14	−63.7 (4)
C2—B2—B8—B9	−1.0 (4)	B12—B15—B19—B14	37.3 (3)
C1—B2—B8—B9	−61.5 (3)	C10—B12—B19—B18	−62.2 (3)
B4—B2—B8—B9	−100.4 (3)	B14—B12—B19—B18	36.9 (3)
B3—B2—B8—B9	37.5 (3)	B11—B12—B19—B18	−0.5 (4)
C1—B4—B8—B3	−1.5 (4)	B15—B12—B19—B18	−101.4 (3)
B10—B4—B8—B3	−100.2 (3)	C10—B12—B19—B15	39.2 (3)
B2—B4—B8—B3	37.8 (3)	B14—B12—B19—B15	138.2 (3)
B5—B4—B8—B3	−63.0 (3)	B11—B12—B19—B15	100.9 (3)
C1—B4—B8—B2	−39.3 (2)	C10—B12—B19—B20	1.6 (4)
B10—B4—B8—B2	−137.9 (3)	B14—B12—B19—B20	100.7 (3)
B5—B4—B8—B2	−100.8 (3)	B11—B12—B19—B20	63.3 (3)
C1—B4—B8—B10	98.7 (3)	B15—B12—B19—B20	−37.6 (3)
B2—B4—B8—B10	137.9 (3)	C10—B12—B19—B14	−99.1 (3)
B5—B4—B8—B10	37.2 (2)	B11—B12—B19—B14	−37.4 (3)
C1—B4—B8—B9	61.9 (3)	B15—B12—B19—B14	−138.2 (3)
B10—B4—B8—B9	−36.8 (2)	B11—B14—B19—B18	−101.1 (3)
B2—B4—B8—B9	101.1 (3)	B13—B14—B19—B18	−37.6 (3)
B5—B4—B8—B9	0.4 (3)	B12—B14—B19—B18	−138.6 (3)
C2—B3—B9—B10	61.8 (3)	B11—B14—B19—B15	−0.1 (4)
B8—B3—B9—B10	−37.7 (3)	B18—B14—B19—B15	101.0 (3)
B2—B3—B9—B10	−0.2 (3)	B13—B14—B19—B15	63.4 (4)
B6—B3—B9—B10	100.6 (3)	B12—B14—B19—B15	−37.6 (3)
C2—B3—B9—B6	−38.9 (2)	B11—B14—B19—B12	37.5 (3)
B8—B3—B9—B6	−138.3 (3)	B18—B14—B19—B12	138.6 (3)
B2—B3—B9—B6	−100.8 (3)	B13—B14—B19—B12	101.0 (3)
C2—B3—B9—B8	99.4 (3)	B11—B14—B19—B20	−63.3 (4)
B2—B3—B9—B8	37.5 (2)	B18—B14—B19—B20	37.8 (3)
B6—B3—B9—B8	138.3 (3)	B13—B14—B19—B20	0.2 (4)
C2—B3—B9—B7	−1.7 (3)	B12—B14—B19—B20	−100.8 (3)
B8—B3—B9—B7	−101.1 (3)	C11—B17—B20—B16	39.4 (3)
B2—B3—B9—B7	−63.6 (3)	C10—B17—B20—B16	98.5 (3)
B6—B3—B9—B7	37.2 (3)	B15—B17—B20—B16	137.7 (3)
C2—B6—B9—B10	−61.9 (3)	C11—B17—B20—B15	−98.3 (3)
B7—B6—B9—B10	37.6 (3)	C10—B17—B20—B15	−39.2 (2)
B1—B6—B9—B10	−0.3 (4)	B16—B17—B20—B15	−137.7 (3)
B3—B6—B9—B10	−100.5 (3)	C11—B17—B20—B19	−61.1 (4)
C2—B6—B9—B3	38.6 (3)	C10—B17—B20—B19	−2.0 (4)
B7—B6—B9—B3	138.1 (3)	B16—B17—B20—B19	−100.5 (3)
B1—B6—B9—B3	100.2 (3)	B15—B17—B20—B19	37.2 (3)
C2—B6—B9—B8	1.5 (4)	C11—B17—B20—B18	2.0 (4)
B7—B6—B9—B8	101.0 (3)	C10—B17—B20—B18	61.1 (4)
B1—B6—B9—B8	63.1 (4)	B16—B17—B20—B18	−37.3 (3)
B3—B6—B9—B8	−37.1 (3)	B15—B17—B20—B18	100.4 (3)
C2—B6—B9—B7	−99.5 (3)	C11—B16—B20—B17	−39.8 (2)
B1—B6—B9—B7	−37.9 (3)	B13—B16—B20—B17	−101.1 (3)
B3—B6—B9—B7	−138.1 (3)	B18—B16—B20—B17	−138.4 (3)
B3—B8—B9—B10	137.8 (3)	C11—B16—B20—B15	−1.5 (4)
B2—B8—B9—B10	100.2 (3)	B17—B16—B20—B15	38.2 (3)
B4—B8—B9—B10	36.7 (3)	B13—B16—B20—B15	−62.9 (4)
B2—B8—B9—B3	−37.6 (3)	B18—B16—B20—B15	−100.2 (3)
B4—B8—B9—B3	−101.1 (3)	C11—B16—B20—B19	61.9 (3)
B10—B8—B9—B3	−137.8 (3)	B17—B16—B20—B19	101.6 (3)
B3—B8—B9—B6	37.3 (3)	B13—B16—B20—B19	0.5 (4)
B2—B8—B9—B6	−0.3 (4)	B18—B16—B20—B19	−36.8 (3)
B4—B8—B9—B6	−63.7 (3)	C11—B16—B20—B18	98.7 (3)
B10—B8—B9—B6	−100.4 (3)	B17—B16—B20—B18	138.4 (3)
B3—B8—B9—B7	100.3 (3)	B13—B16—B20—B18	37.3 (3)
B2—B8—B9—B7	62.7 (3)	C10—B15—B20—B17	39.9 (2)
B4—B8—B9—B7	−0.8 (4)	B19—B15—B20—B17	138.4 (3)
B10—B8—B9—B7	−37.5 (3)	B12—B15—B20—B17	100.8 (3)
B6—B7—B9—B10	−137.9 (3)	C10—B15—B20—B16	1.8 (4)
B5—B7—B9—B10	−36.6 (3)	B19—B15—B20—B16	100.4 (3)
B1—B7—B9—B10	−100.2 (3)	B17—B15—B20—B16	−38.1 (3)
B6—B7—B9—B3	−37.4 (3)	B12—B15—B20—B16	62.8 (3)
B5—B7—B9—B3	63.9 (3)	C10—B15—B20—B19	−98.5 (3)
B1—B7—B9—B3	0.2 (4)	B17—B15—B20—B19	−138.4 (3)
B10—B7—B9—B3	100.4 (3)	B12—B15—B20—B19	−37.6 (3)
B5—B7—B9—B6	101.3 (3)	C10—B15—B20—B18	−61.8 (3)
B1—B7—B9—B6	37.6 (3)	B19—B15—B20—B18	36.8 (3)
B10—B7—B9—B6	137.9 (3)	B17—B15—B20—B18	−101.7 (3)
B6—B7—B9—B8	−100.5 (3)	B12—B15—B20—B18	−0.8 (4)
B5—B7—B9—B8	0.9 (4)	B18—B19—B20—B17	101.0 (3)
B1—B7—B9—B8	−62.8 (3)	B15—B19—B20—B17	−37.6 (3)
B10—B7—B9—B8	37.4 (3)	B12—B19—B20—B17	0.3 (4)
C1—B4—B10—B5	38.9 (2)	B14—B19—B20—B17	63.5 (4)
B2—B4—B10—B5	100.9 (3)	B18—B19—B20—B16	37.1 (3)
B8—B4—B10—B5	138.4 (3)	B15—B19—B20—B16	−101.5 (3)
C1—B4—B10—B9	−62.3 (3)	B12—B19—B20—B16	−63.6 (4)
B2—B4—B10—B9	−0.3 (3)	B14—B19—B20—B16	−0.4 (4)
B8—B4—B10—B9	37.2 (3)	B18—B19—B20—B15	138.6 (3)
B5—B4—B10—B9	−101.2 (3)	B12—B19—B20—B15	37.9 (3)
C1—B4—B10—B8	−99.5 (3)	B14—B19—B20—B15	101.1 (3)
B2—B4—B10—B8	−37.5 (2)	B15—B19—B20—B18	−138.6 (3)
B5—B4—B10—B8	−138.4 (3)	B12—B19—B20—B18	−100.7 (3)
C1—B4—B10—B7	1.6 (4)	B14—B19—B20—B18	−37.5 (3)
B2—B4—B10—B7	63.6 (3)	B19—B18—B20—B17	−101.1 (3)
B8—B4—B10—B7	101.1 (3)	B14—B18—B20—B17	−63.0 (4)
B5—B4—B10—B7	−37.3 (3)	B13—B18—B20—B17	0.4 (4)
C1—B5—B10—B4	−38.9 (2)	B16—B18—B20—B17	37.4 (3)
B7—B5—B10—B4	−138.3 (3)	B19—B18—B20—B16	−138.6 (3)
B1—B5—B10—B4	−100.2 (3)	B14—B18—B20—B16	−100.4 (3)
C1—B5—B10—B9	62.4 (3)	B13—B18—B20—B16	−37.0 (3)
B7—B5—B10—B9	−37.0 (3)	B19—B18—B20—B15	−36.9 (3)
B4—B5—B10—B9	101.3 (3)	B14—B18—B20—B15	1.2 (4)
B1—B5—B10—B9	1.1 (4)	B13—B18—B20—B15	64.7 (4)
C1—B5—B10—B8	−1.5 (3)	B16—B18—B20—B15	101.7 (3)
B7—B5—B10—B8	−100.9 (3)	B14—B18—B20—B19	38.1 (3)
B4—B5—B10—B8	37.4 (3)	B13—B18—B20—B19	101.6 (3)
B1—B5—B10—B8	−62.8 (3)	B16—B18—B20—B19	138.6 (3)
C1—B5—B10—B7	99.4 (3)	N2—C3—N1—C4	11.0 (5)
B4—B5—B10—B7	138.3 (3)	C1—C3—N1—C4	−167.5 (3)
B1—B5—B10—B7	38.1 (3)	N2—C3—N1—SN	−176.0 (2)
B3—B9—B10—B4	0.3 (3)	C1—C3—N1—SN	5.6 (4)
B6—B9—B10—B4	63.7 (3)	C5—C4—N1—C3	−65.8 (4)
B8—B9—B10—B4	−37.2 (3)	C6—C4—N1—C3	62.9 (4)
B7—B9—B10—B4	101.1 (3)	C5—C4—N1—SN	121.0 (3)
B3—B9—B10—B5	−64.0 (3)	C6—C4—N1—SN	−110.3 (3)
B6—B9—B10—B5	−0.5 (4)	N1—C3—N2—C7	−175.9 (3)
B8—B9—B10—B5	−101.4 (3)	C1—C3—N2—C7	2.3 (6)
B7—B9—B10—B5	36.8 (3)	C9—C7—N2—C3	114.4 (4)
B3—B9—B10—B8	37.5 (2)	C8—C7—N2—C3	−125.9 (4)
B6—B9—B10—B8	100.9 (3)	N4—C12—N3—C13	12.1 (5)
B7—B9—B10—B8	138.2 (3)	C10—C12—N3—C13	−166.7 (3)
B3—B9—B10—B7	−100.8 (3)	N4—C12—N3—SN	−172.5 (3)
B6—B9—B10—B7	−37.3 (3)	C10—C12—N3—SN	8.8 (4)
B8—B9—B10—B7	−138.2 (3)	C15—C13—N3—C12	−65.4 (4)
B3—B8—B10—B4	101.0 (3)	C14—C13—N3—C12	64.0 (4)
B2—B8—B10—B4	37.5 (2)	C15—C13—N3—SN	119.0 (3)
B9—B8—B10—B4	138.6 (3)	C14—C13—N3—SN	−111.5 (3)
B3—B8—B10—B5	63.4 (3)	N3—C12—N4—C16	−177.1 (4)
B2—B8—B10—B5	−0.1 (4)	C10—C12—N4—C16	1.6 (7)
B4—B8—B10—B5	−37.6 (3)	C18—C16—N4—C12	−112.1 (5)
B9—B8—B10—B5	101.0 (3)	C17—C16—N4—C12	126.5 (5)

## References

[bb1] Altomare, A., Burla, M. C., Camalli, M., Cascarano, G. L., Giacovazzo, C., Guagliardi, A., Moliterni, A. G. G., Polidori, G. & Spagna, R. (1999). *J. Appl. Cryst.* **32**, 115–119.

[bb2] Belmont, J. A., Soto, J., King, R. E. III, Donaldson, A. J., Hewes, J. D. & Hawthorne, M. F. (1989). *J. Am. Chem. Soc.* **111**, 7475–7486.

[bb3] Brandenburg, K. (1999). *DIAMOND*. Crystal Impact GbR, Bonn, Germany.

[bb4] Brown, A. D., Colquhoun, H. M., Daniels, A. J., MacBride, J. A. H., Stephenson, I. R. & Wade, K. (1992). *J. Mater. Chem.* **2**, 793–804.

[bb5] Collins, S. (2011). *Chem. Rev.* **214**, 91–141.

[bb6] Devi, A. (2013). *Coord. Chem. Rev.* **257**, 3332–3384.

[bb7] Dröse, P., Hrib, C. G. & Edelmann, F. T. (2010). *J. Am. Chem. Soc.* **132**, 15540–15541. 10.1021/ja108051u 20961049

[bb8] Edelmann, F. T. (2008). *Adv. Organomet. Chem.* **57**, 183–352.

[bb9] Edelmann, F. T. (2013*a*). *Adv. Organomet. Chem.* **61**, 55–374.

[bb10] Edelmann, F. T. (2013*b*). *Z. Anorg. Allg. Chem.* **639**, 655–667.

[bb11] Felekidis, A., Goblet-Stachow, M., Liégeois, J. F., Pirotte, B., Delarge, J., Demonceau, A., Fontaine, M., Noels, A. F., Chizhevsky, I. T., Zinevich, T. V., Bregadze, V. I., Dolgushin, F. M., Yanovsky, A. I. & Struchkov, Y. T. (1997). *J. Organomet. Chem.* **536–537**, 405–412.

[bb12] Groom, C. R., Bruno, I. J., Lightfoot, M. P. & Ward, S. C. (2016). *Acta Cryst.* B**72**, 171–179. 10.1107/S2052520616003954 PMC4822653 27048719

[bb13] Harmgarth, N., Gräsing, D., Dröse, P., Hrib, C. G., Jones, P. G., Lorenz, V., Hilfert, L., Busse, S. & Edelmann, F. T. (2014). *Dalton Trans.* **43**, 5001–5013. 10.1039/c3dt52751d 24202239

[bb14] Harmgarth, N., Liebing, P., Förster, A., Hilfert, L., Busse, S. & Edelmann, F. T. (2017). *Eur. J. Inorg. Chem. DOI*, **10**, 1002ejic. 201700500.

[bb15] Hillebrand, P., Hrib, C. G., Harmgarth, N., Jones, P. G., Lorenz, V., Kühling, M. & Edelmann, F. T. (2014). *Inorg. Chem. Commun.* **46**, 127–129.

[bb16] Junk, P. C. & Leary, S. G. (2000). *Z. Anorg. Allg. Chem.* **626**, 2279–2283.

[bb17] Li, Z., Barry, S. T. & Gordon, R. G. (2005). *Inorg. Chem.* **44**, 1728–1735. 10.1021/ic048492u 15762699

[bb18] Lim, B. S., Rahtu, A., Park, J.-S. & Gordon, R. G. (2003). *Inorg. Chem.* **42**, 7951–7958. 10.1021/ic0345424 14632513

[bb19] Murphy, D. M., Mingos, D. M. P., Haggitt, J. L., Powell, H. R., Westcott, S. A., Marder, T. B., Taylor, N. J. & Kanis, D. R. (1993). *J. Mater. Chem.* **3**, 139–148.

[bb20] Sheldrick, G. M. (2015). *Acta Cryst.* C**71**, 3–8.

[bb21] Stoe & Cie (2002). *X-AREA and *X-RED**. Stoe & Cie, Darmstadt, Germany.

[bb22] Teixidor, F., Flores, M. A., Viñas, C., Kivekäs, R. & Sillanpää, R. (1996). *Angew. Chem. Int. Ed. Engl.* **35**, 2251–2253.

[bb23] Valliant, J. F., Guenther, K. J., King, A. S., Morel, P., Schaffer, P., Sogbein, O. O. & Stephenson, K. (2002). *Coord. Chem. Rev.* **232**, 173–230.

[bb24] Westrip, S. P. (2010). *J. Appl. Cryst.* **43**, 920–925.

[bb25] Xu, B., Yao, Z.-J. & Jin, G.-X. (2014). *Russ. Chem. Bull.* **63**, 963–969.

[bb26] Yao, Z.-J. & Jin, G.-X. (2012). *Organometallics*, **31**, 1767–1774.

[bb27] Yao, Z.-J. & Jin, G.-X. (2013). *Coord. Chem. Rev.* **257**, 2522–2535.

[bb28] Yao, Z.-J., Lin, Y.-J., Li, Z.-H. & Jin, G.-X. (2013). *Chem. Eur. J.* **19**, 2611–2614. 10.1002/chem.201203850 23296574

[bb29] Yao, Z.-J., Su, G. & Jin, G.-X. (2011). *Chem. Eur. J.* **17**, 13298–13307. 10.1002/chem.201101638 22009699

[bb30] Yao, Z.-J., Xu, B., Su, G. & Jin, G.-X. (2012). *J. Organomet. Chem.* **721–722**, 31–35.

